# Experimental Models of Hypertrophic Cardiomyopathy: A Systematic Review

**DOI:** 10.1016/j.jacbts.2024.10.017

**Published:** 2025-01-15

**Authors:** Floor W. van den Dolder, Rafeeh Dinani, Vincent A.J. Warnaar, Sofija Vučković, Adriana S. Passadouro, Ali A. Nassar, Azhaar X. Ramsaroep, George B. Burchell, Linda J. Schoonmade, Jolanda van der Velden, Birgit Goversen

**Affiliations:** a Department of Physiology, Amsterdam University Medical Center (UMC), Location VUmc, Amsterdam, the Netherlands; b Amsterdam Cardiovascular Sciences, Heart Failure and Arrhythmias, Amsterdam, the Netherlands; c Vascular Surgery, Department of Molecular Medicine and Surgery, Karolinska University Hospital and Karolinska Institutet, Stockholm, Sweden; d Laboratory Genetic Metabolic Diseases, Amsterdam University Medical Center (UMC), University of Amsterdam, Amsterdam, the Netherlands; e Amsterdam Gastroenterology, Endocrinology and Metabolism, Amsterdam, the Netherlands; f Medical Library, Vrije Universiteit Amsterdam, Amsterdam, the Netherlands

**Keywords:** animal models, human iPSC-derived cardiomyocytes, hypertrophic cardiomyopathy, stem cells, systematic review

## Abstract

To advance research in hypertrophic cardiomyopathy (HCM), and guide researchers in choosing the optimal model to answer their research questions, we performed a systematic review of all models investigating HCM induced by gene variants ranging from animal models to human induced pluripotent stem cell–derived cardiomyocytes (hiPSC-CMs). Our research question entailed: which experimental models of HCM have been created thus far, and which major hallmarks of HCM do they present? Out of the 603 included papers, the majority included animal models, though a clear transition to hiPSC-CM is visible since 2010. Our review showed that only 36 mouse models showed minimal 4 out of 6 HCM disease markers (cell/cardiac hypertrophy, disarray, fibrosis, diastolic dysfunction, and arrhythmias), while only 17 hiPSC-CM models showed 3 out of 4 HCM cell characteristics. Our review emphasizes the need to better report data on sample size, sex, age, and relevant disease-specific characteristics.

## Highlights

• This systematic review provides an overview of all experimental HCM models from 1977 to 2023 and highlights if models show main human disease characteristics.
• Since ∼2010, there has been a shift from studies in animal models to animal-free stem cell–derived heart models to study cellular pathomechanisms and test interventions.
• Our analyses illustrate that 11.5% of all HCM mouse studies report the presence of minimal 4 out of 6 disease characteristics and 17.3% of hiPSC-CM mutant lines report the presence of minimal 3 out of 4 CM disease features.
• Research on HCM can be strengthened by optimal data reporting (sex, age, sample size, genotype), blinding of experiments/analyses, and complete analyses of main disease characteristics at the cell and whole heart levels.

Hypertrophic cardiomyopathy (HCM) is the most common heritable cardiac disease and is defined as the presence of increased left ventricular (LV) wall thickness or mass not solely explained by abnormal loading conditions.^1^^,^^2^ Heterozygous pathogenic variants in genes encoding sarcomere proteins represent the most frequent cause of HCM. Since the discovery of the first pathogenic variant (ie, mutation) in the gene encoding myosin heavy chain,^3^ various in vivo and in vitro models have been generated to gain insight into the pathomechanism of this disease. These models have paved the way for a better understanding of alterations at the cellular and tissue levels during progression of HCM. However, current experimental models do not entirely represent early and advanced disease stages, which may be due to intrinsic differences in cardiac physiology between rodents and human and/or the absence of comorbidities and complex genetic background present in the human patient population, while stem cell–derived heart models are limited to mutation-mediated cellular/tissue changes. Regardless of the limitations of current experimental models, findings from these models have improved our knowledge and helped us to have a clearer picture of changes due to HCM mutations, which has led to the discovery of novel treatments such as myosin inhibitors.^4^ To advance research in HCM, and guide researchers in choosing the optimal model to answer their research questions, we provide an overview of genetic HCM models used in the period of 1977 to 2023, including main HCM hallmarks such as impaired relaxation (ie, diastolic dysfunction), hypertrophy, fibrosis, myocardial or sarcomere disarray, and ventricular/cellular arrhythmias. Thereto, we have performed a systematic review of all studies investigating HCM that have used experimental models ranging from animal models to more recent induced pluripotent stem cell–derived models.

## Methods

We conducted this review based on the PRISMA (Preferred Reporting Items for Systematic Reviews and Meta-Analyses) statement. The extensive protocol for this systematic review is registered at PROSPERO (CRD42022334148).

### Defining the research question

In this systematic review, we aimed to scope the existing literature in which genetic HCM has been studied using preclinical models such as animal or cellular models. Therefore, our research question entailed: which experimental models of HCM have been created thus far, and which major hallmarks of HCM do they present?

### Selecting databases and the search terms

A systematic search was performed in PubMed, Embase, and Clarivate Analytics/Web of Science Core Collection in September 2020. On March 1, 2023, an update on the existing search was performed and only the newly published literature between the first and last searches (September 2020 to March 1, 2023) was considered in the second phase of screening. The search included the keywords and free text terms for (synonyms of) “hypertrophic cardiomyopathy” combined with (synonyms of) “animal models” and “cellular models.” No limitations on date or language were applied in the search. A full overview of the search terms per database can be found in the Supplemental Methods.

### Title and abstract screening

The result of the search was imported into the reference management software (Rayyan). Two independent screeners (R.D. and S.V.) screened all the papers using inclusion/exclusion criteria. The inclusion criteria for this stage were whether the study describes using an animal or cellular model to study genetic HCM.

The exclusion criteria were as follows: 1) if the paper describes only human HCM tissue samples or isolated human cells, or diagnosis/risk prediction in human subjects; 2) if the study described a computational model to study HCM; 3) if the paper describes the symptoms of HCM due to a secondary cause (eg, hypertension, angiotensin II overload, or transverse aortic constriction) in animals; 4) if the paper describes in vitro molecular studies on mutant (purified) proteins; and 5) if the paper is not in English.

After the initial data extraction, we excluded other studies: if the study describes the symptoms of HCM due to the presence of another (metabolic) disease, such as Barth syndrome, Danon disease, Duchenne muscular dystrophy, Friedreich’s ataxia, Leopard syndrome/Noonan syndrome, and Pompe disease.

To resolve areas of disagreement, a third independent reviewer (F.W.v.d.D.) screened the studies with conflict decisions from the primary 2 reviewers. For the updated search, the abstracts were screened by 2 independent reviewers (R.D. and B.G.) according to the same inclusion and exclusion criteria. Areas of disagreement were resolved by a third independent reviewer (J.v.d.V.).

### Full-text screening

An EndNote library with all included studies and their PDFs was generated. Two independent reviewers (R.D. and S.V.) decided on the inclusion and exclusion of the studies. The same inclusion and exclusion criteria as in the abstract screening were applied in this phase. Publication types that did not include peer-reviewed papers, such as conference abstracts or posters, were excluded. Also, publications in which a full-text PDF could not be traced were excluded. The studies with conflict decisions were reviewed by a third reviewer (B.G.). For the updated search, full-text screening was performed by 2 independent reviewers (R.D. and B.G.). Conflict decisions were reviewed by a third reviewer (J.v.d.V.).

### Data extraction

In this phase, an Excel (Microsoft) sheet for data extraction, quality assessment, and risk of bias was generated. Reviewers were asked to extract data, following the guideline described in Supplemental Methods, regarding: 1) the general information of the paper, such as the year of publication and the country of the corresponding author; 2) the gene of interest, the mutation, the percentage of mutant protein, and how the mutation was confirmed; 3) the description of the animal model, such as strain or breed, age, sex, and which control group was used; 4) the description of the cell-based model, such as the cell type, the source of the cells, the sex, and which control group was used; and 5) changes in the major hallmarks of HCM compared with the control group, such as impaired relaxation (ie, diastolic dysfunction), cardiac hypertrophy, cardiomyocyte [CM] hypertrophy, fibrosis, myocardial or sarcomere disarray, and cellular/ventricular arrhythmias. For the cell-based models, we did not include cardiac hypertrophy and fibrosis for the data extraction.

We based the quality assessment on the CAMARADES checklist for study quality and we tailored the SYRCLE risk-of-bias tool for the risk-of-bias assessment. Each study was reviewed by 2 independent reviewers (F.W.v.d.D, R.D., V.A.J.W., S.V., A.S.P., A.A.N., A.X.R., or B.G.). The areas of disagreement were resolved by consensus and in exceptions by a third independent reviewer (B.G. or J.v.d.V.). We aimed to find all relevant studies and would like to apologize in advance for studies that may have not been included in our screening.

## Results

**Figure 1:**
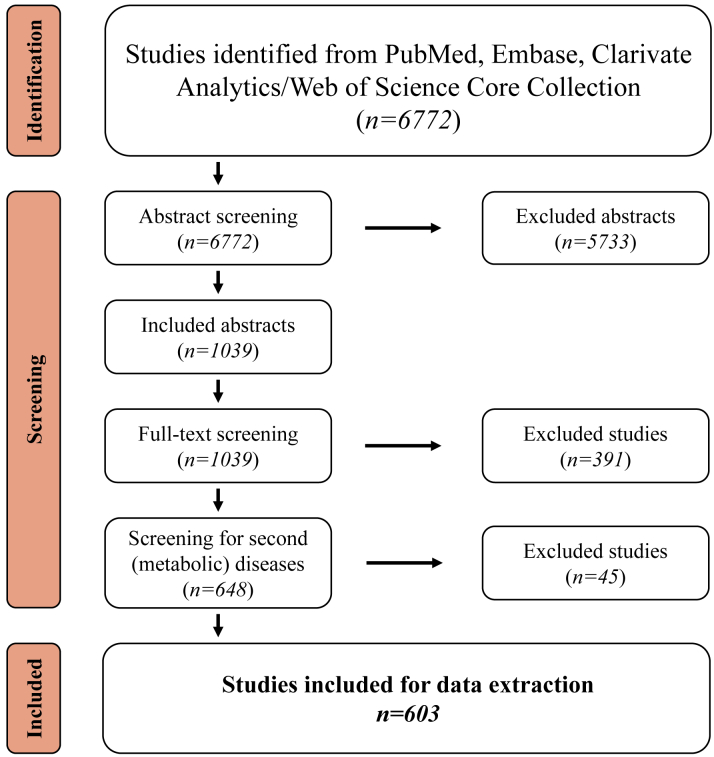
PRISMA Flow Diagram of the Literature Screening Process PRISMA = Preferred Reporting Items for Systematic Reviews and Meta-Analyses.

Figure 1 and the “methods” illustration on the Central Illustration summarizes the search results. Briefly, the database search identified 6,772 studies. By reading the abstracts and full-text, 6,169 studies were excluded because they did not meet our inclusion criteria. Data were extracted from the remaining 603 studies that met the inclusion criteria.

**Central Illustration:**
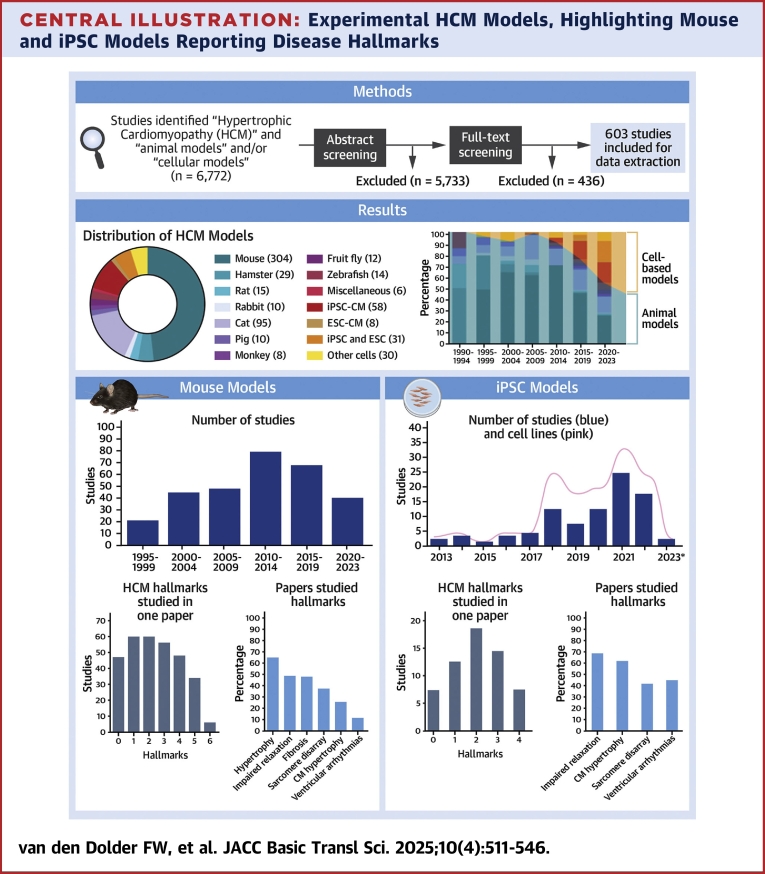
Experimental HCM Models, Highlighting Mouse and iPSC Models Reporting Disease Hallmarks The **methods** illustration shows the PRISMA flow diagram of the literature screening process. The **results** illustrate the distribution of animal and cell-based models in HCM studies, with “n-numbers” indicating the studies that describe the use of specific models. Additionally, the number of studies is normalized to the total number of studies per 5-year period, highlighting the increasing trend of cell-based studies compared to animal models over time. The “Mouse Models” and “iPSC Models” illustrations display the number of publications published over time, the number of hallmarks described per paper, and how many papers studied the same hallmark. ∗Until April. ESC = embryonic stem cell; ESC-CM = embryonic stem cell–derived cardiomyocytes; HCM = hypertrophic cardiomyopathy; iPSC = induced pluripotent stem cell; iPSC-CMs, induced pluripotent stem cell–derived cardiomyocyte.

### Study characteristics

Out the 603 studies, 420 (69.7%) involved animal models, 101 (16.8%) used cell-based models, and 82 (13.6%) studies combined animal models (in vivo and ex vivo) and cell-based models (in vitro). Among the combination studies, 53 used an animal model alongside isolated ventricular cells from the same model, while the remaining 21 combined different animal and cell-based models.

**Figure 2:**
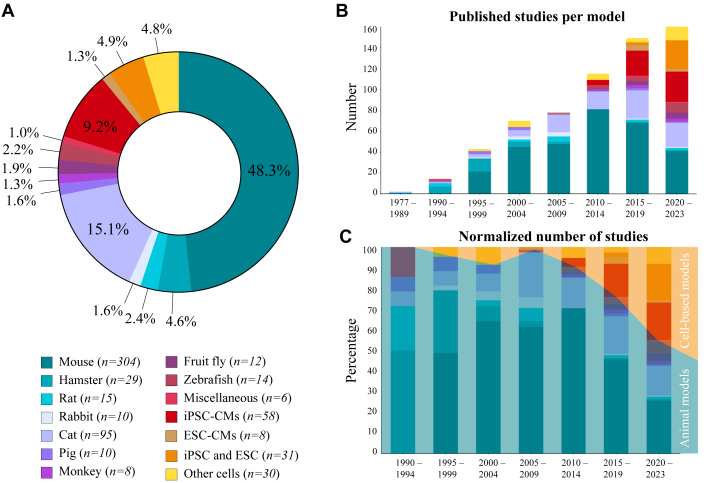
Number of Studies per Model (A) Distribution of animal and cell-based models in hypertrophic cardiomyopathy studies. Numbers indicate the studies that describe the use of the specific model. (B) Hypertrophic cardiomyopathy models over time (per 5-year period) specified by model type. (C) Number of studies normalized to the total number of studies per 5-year period. Increasing trend of cell-based studies compared with animal models over time. ESC = embryonic stem cell; ESC-CM = embryonic stem cell–derived cardiomyocytes; iPSC = induced pluripotent stem cell; iPSC-CMs, induced pluripotent stem cell–derived cardiomyocyte.

The majority of the studies used mice for in vivo, ex vivo, and/or in vitro work (n = 304) (Figure 2A, Central Illustration). Other rodent studies included hamsters (n = 29), rats (n = 15), and rabbits (n = 10). Studies in companion animals involved cats (n = 95), and to a smaller extent dogs (n = 3), while large animal models ranged from pigs (n = 10), monkeys (n = 8), alpaca (n = 1), and kangaroo (n = 1). Non-mammal models involved zebrafish (n = 14), fruit fly (n = 12), and rice fish (n = 1). The dog, alpaca, kangaroo, and rice fish are categorized in the miscellaneous group due to the low number of studies. The cell-based models consisted of isolated cells, not included in the previous animal totals, or cell lines used to transfect (likely) pathogenic mutations for in vitro analyses of HCM (n = 30). Thirty-one studies described the generating of new cell lines with a gene mutation without reporting data related to CM phenotype. Other cell-based studies involved induced pluripotent stem cell–derived cardiomyocytes (iPSC-CMs) (n = 58) or embryonic stem cell–derived cardiomyocytes (ESC-CMs) (n = 8). The total number of experimental models exceeds 603 (Figure 2A) because several studies used multiple animal models,^5^ cell-based models,^6^, ^7^, ^8^, ^9^ or a combination of 2^10^, ^11^, ^12^, ^13^, ^14^, ^15^, ^16^, ^17^, ^18^, ^19^, ^20^, ^21^, ^22^, ^23^, ^24^, ^25^, ^26^, ^27^ or 3^28^, ^29^, ^30^ different models.

The number of HCM studies using experimental models has steadily increased over the years, with nearly half of the papers included in this review published in the last decade: 2015 to 2019 (n = 142) and 2020 until April 2024 (n = 147) (Figure 2B). The earliest HCM models, before 1995, involved hamsters,^31^, ^32^, ^33^, ^34^, ^35^, ^36^, ^37^ rats,^38^, ^39^, ^40^, ^41^ cats,^5^^,^^42^ dogs,^5^ pigs,^43^ and kangaroo.^44^ In subsequent years, additional animal models were published using advancements in gene editing and natural occurrence of HCM: mice in 1995,^45^ rabbits in 1999,^46^ fruit flies in 2005,^47^ alpaca in 2010,^48^ zebrafish in 2011,^49^ monkeys in 2013,^50^ and rice fish in 2019.^51^ The first ESC-CM study appeared in 2005,^52^ the first human induced pluripotent stem cell–derived cardiomyocytes (hiPSC-CMs) in 2013,^53^^,^^54^ and the first paper describing the generation of HCM lines was published in 2017.^55^ The use of in vitro models over animal models (in vivo and ex vivo) has increased in recent years (Figures 2B and 2C, Central Illustration) and is expected to further increase in coming years growing.^56^

### Animal models

As described previously, a wide range of animals, including rodents, companion animals, large animals, and nonmammals, have been used to better understand HCM pathophysiology. While most studies describe animal models with induced HCM, spontaneous occurrence has been observed in mouse (n = 2), hamsters (n = 29), rats (n = 6), rabbits (n = 1), cats (n = 95), dogs (n = 3), kangaroo (n = 1), pig (n = 7), monkeys (n = 8), and alpaca (n = 1).

### Mouse models

**Figure 3:**
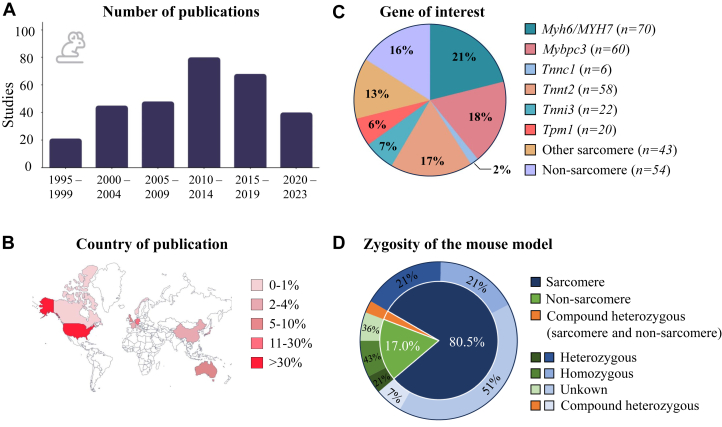
General Information on Mouse Studies (A) The numbers of mouse studies published per 5-year period. (B) Geographical distribution of research on mouse models based on the country of corresponding author. Each country is shaded according to the percentage of total mouse model publications. (C) Distribution of gene mutations in which each segment represents the percentage of studies on a specific gene in their mouse model. All genes that translate to sarcomere proteins are grouped as “other sarcomere” except for *Myh6/MYH7*, *Mybpc3*, *Tnnc1*, *Tnnt2*, *Tnni3*, and *Tpm1*. Genes not translating to sarcomere proteins are grouped as “non-sarcomere.” (D) Overview of zygosity, in which categorization of mouse models is based on their zygosity in relation to sarcomere mutations, non-sarcomere mutations, and compound heterozygous mutations.

The mouse (*Mus musculus*) is the most abundant HCM animal model with 304 papers (Figure 3A, Central Illustration, Supplemental References). The distribution of mouse studies over the world is illustrated in Figure 3B. Diverse gene editing tools were applied ranging from targeted deletion of a gene of interest (knockout [KO]), transgenic overexpression of a mutant protein, or more recently CRISPR/Cas9-induced gene mutations.^56^ More than 80% of the mouse models bear a sarcomere gene mutation. The highest number of mouse model publications (n = 188) relate to the 3 most frequently affected human disease genes: *Myh6/Myh7* (n = 70), *Mybpc3* (n = *60*), and *Tnnt2* (n = 58) encoding for myosin heavy chain, cardiac myosin binding protein C, and cardiac troponin T, respectively. Fewer papers report on mutations in cardiac troponin I (*Tnni3*) (n = 23, 9 mutations), tropomyosin (*Tpm1*) (n = 20, 4 mutations), and cardiac troponin C (*Tnnc1*) (n = 6, 2 mutations), and 43 papers report on mutations in other sarcomere genes (alpha actin [*Actc1*], ventricular essential myosin light chain [*Elc1v*], myosin regulatory light chain 2 [*Myl2*], myosin essential light chain 3 [*Myl3*], and titin [*Ttn*]). We identified 54 papers examining mouse models harboring a mutation in non-sarcomere genes (Figure 3C). Various secondary disease factors are described, with aging being the most prominent (n = 107), followed by exercise (n = 16), and diet (n = 4).

Notably, 66 out of the 70 myosin heavy chain papers included a mouse model with the first identified HCM mutation, R403Q. In addition, 5 other mutations were reported: R453C (n = 4), V606M (n = 2), R719W (n = 4), D778G (n = 1), and G741R (n = 1), which are all reported as pathogenic in human HCM. The R403Q mutation was mostly studied in the *Myh6* (mouse) background, while 3 studies reported data on the effect of R403Q on the human *Myh7* background. Miller et al^57^ studied the effects of D778G and G741R on a *Myh7* background. The R403Q *Myh6* mouse studies included heterozygous (n = 48) and homozygous (n = 8) mice, and in several studies a secondary disease modifier was introduced in addition to R403Q. Two papers generated mice that in addition to a R403Q mutation also overexpressed β2-adrenergic receptor or β-adrenergic receptor kinase carboxyl-terminus to modulate particular aspects of β-adrenergic receptor signaling.^2^^,^^58^ Similarly, Hardt et al^59^ modified β-AR stimulation by overexpressing the G protein α subunit to enhance contractile and relaxation responses in a R403Q mouse model. *Lpar1* (lysophosphatidic acid receptor 1) stimulates fibrosis in multiple organs, including the heart. To attenuate fibrosis in a R403Q HCM mice, *Lpar1* was knocked down.^60^ Tetracycline-controlled transcriptional activation was used to induce expression of R403Q *Myh6*, allowing reversible (de)activation through the presence of doxycycline.^61^

Out of the 60 papers describing *Mybpc3* mouse models, *Mybpc3* KO (n = 27) was reported most frequently, followed by transgenic mutant overexpression (n = 25), followed by truncating mutations at the C-terminus (n = 7) or at the N-terminus (n = 1), or due to the W792fs mutation (n = 1). Nearly all *Mybpc3* KO mouse studies published in Europe refer to Carrier et al (n = *11*)^62^ while papers published in the United States refer to Harris et al (n = *14*).^63^ All 25 papers researching *Mybpc3* transgenic mice refer to the originally generated model by Vignier et al.^64^ This model carries a G>A point mutation on the last nucleotide of exon 6, which is a founder mutation in Tuscany.^64^ In contrast to *Myh6* models, *Mybpc3* mice models are reported more as homozygous (n = 35) than heterozygous (n = 24), and 4 studies included mice with compound heterozygous mutations (phospholamban [*Pln*], *P53*, transforming growth factor beta receptor 2 [*Tgfbr2*], and protein phosphatase I inhibitor [*I-1*]).^65^, ^66^, ^67^, ^68^

Publications on *Tnnt2*-linked HCM included 10 gene variants, though not all variants have been reported as pathogenic in human: I79N (n = 10), R92L (n = 9), R92Q (n = 33), R92W (n = 7), R97L (n = 1) (conflicting pathogenicity data in human; human variant is R94L), F110I (n = 3), Delta160 (n = 6) (conflicting pathogenicity data in human), E163R (n = 3) (pathogenicity unknown), and R278C (n = 5) (variant of unknown significant). While most mouse studies involved the *Myh6* background, the R92L, R92Q, and R97L (R94L) were also studied on a *Myh7* background.

Gene variants in HCM patients are inherited in an autosomal dominant manner; therefore, homozygosity in these patients is uncommon.^69^ In 21% of studies, mice carried either a heterozygous or homozygous sarcomere mutation, while the zygosity is unknown or not mentioned in more than 50% of papers. In 64% of cases, the zygosity of mice with a non-sarcomere mutation is documented, with 21% being heterozygous and 43% homozygous. Compound heterozygous mice are mentioned in 7% of papers exhibiting mutations in sarcomere and/or non-sarcomere genes (Figure 3D). Mice carrying a mutation in *Myh6* are more often studied on a heterozygous (58.5%) than homozygous (9.8%) background, while this is the opposite for *Mybpc3* mice with 27.8% using heterozygous and 48.1% homozygous mice. The zygosity is not reported in 66.7% of the *Tnnt2* mice.

Sex is not consistently documented in papers (n = 120), but there is a preference for studying solely male mice (n = 108), followed by mixed-sex groups (n = 84) and studies exclusively involving female mice (n = 34) (Supplemental Figure 1). More than half (53.8%) of the studies involving *Myh6* mice used only male mice, which is more compared with *Mybpc3* (14.3%) and *Tnnt2* (23.1%). The same holds true for the use of female mice that are used in 15.4% of the *Myh6* studies, 3.8% in *Tnnt2* studies, and only 2.2% in *Mybpc3*. *Tnnt2* mouse studies use a group of mixed sex in 26.9% of the papers, while this is only 17.6% and 11.0% for *Mybpc3* and *Myh6*, respectively.

We categorized the mice in different age groups. Mice 0 to 3, 4 to 6, 7 to 9, and 10 to 12 months of age were used in 28.6%, 28.9%, 15.2%, and 9.6% of the studies, respectively. Mice older than 1 year of age were studied in 8.5% of papers, while age was not reported in 11.2% of papers (Supplemental Figure 1). Focusing on the 3 most commonly studied sarcomere genes reveals that mice with a different genetic background are studied at different ages. The percentage per age group of mice with a *Myh6* mutation, is similar to the overall average across all mouse studies, except for the 10- to 12-month age group (19.1% vs 9.6%). For *Mybpc3* mice, 41% of studies used mice 0 to 3 months of age, compared with 28.6% of the total mouse studies. In contrast, *Tnnt2* mice are most frequently studied in the 4- to 6-month age group, with 51.9% of studies, while the percentage is only 28.9% of all mouse studies.

Wild-type mice (32.4%) are the most utilized control animals in mouse studies, with 10.1% using age-matched wild-type, 4.1% using sex-matched wild-type, and 6.9% using age- and sex-matched wild-type mice. In an additional 36 (11.8%) papers, control animals were described as littermates, and in 31 (10.1%) papers, nontransgenic mice were used as control animals (Supplemental Table 1). The C57BL/6 strain was used in one-fourth (24.5%) of all studies, of which 10.5% specifies the C57BL/6J substrain and 1.9% the C57BL/6N substrain. Other commonly used strains are Black Swiss (9.3%), FVB/N (7.1%), B6SJL (4.3%), and 129SvEv (4%). In more than 28% of the publications, the strain was not reported (Supplemental Table 2).

### HCM characteristics in mouse models

**Figure 4:**
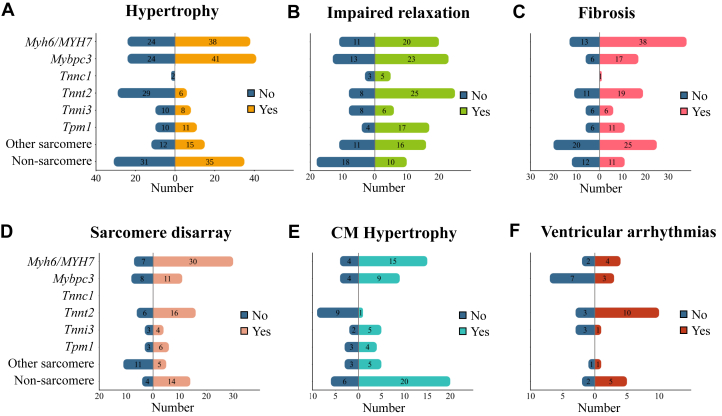
HCM Hallmarks in Mouse Models (A-F) The numbers are based on the extracted data related to hypertrophic cardiomyopathy (HCM) hallmarks including hypertrophy, impaired relaxation, fibrosis, sarcomere disarray, cardiomyocyte (CM) hypertrophy, and ventricular arrhythmias. An HCM hallmark is identified as “yes” when a significant difference between HCM model and control model is observed. If no significant difference is found, the HCM hallmark is marked as “no,” indicating that it was investigated but not identified. A single study may investigate multiple models (eg, strain, sex, or age), allowing it to contribute to both “yes" and “no.” Additionally, different genetic backgrounds studied in 1 paper can result in contributions multiple gene groups.

Hypertrophy (n = 197 of 304 [65%]) is the most extensively studied hallmark of HCM, with 76% (n = 146 of 197) of the papers reporting hypertrophy in at least 1 of the mutant mouse models. Hypertrophy is mainly reported as an elevation in the heart weight-to-body weight ratio compared with control animals, both in heterozygous (n = 46) and homozygous (n = 48) mutant mice. A total of 20 studies compared age-matched male with female mice and reported hypertrophy solely in male (n = 5 of 20), in female (n = 3 of 20), or both sexes (n = 4 of 20). However, in most cases, hypertrophy is not reported in either (n = 8 of 20). Mice harboring mutations in *Mybpc3* (n = 41 [26.6%]) and *Myh6* (n = 38 [24.7%]) exhibit the highest incidence of hypertrophy compared with *Tnnt2* (n = 6 [3.9%]), *Tnni3* (n = 8 [5.2%]), and *Tnnc1* (no report). Furthermore, mice carrying nonsarcomere mutations (n = 35 [22.7%]) demonstrate the next highest occurrence of hypertrophy after *Mybpc3* and *Myh6* (Figure 4A).

Following hypertrophy, impaired relaxation emerges as the second-most-studied HCM hallmark in mice (n = 145 of 304 [48%]). Echocardiography provides a reliable and reproducible method to assess cardiac function in rodents.^70^ Although this method is not yet standardized, it is the most used method for assessing diastolic function in the papers that we included. Impaired relaxation was observed in 120 papers, of which only 9 papers studied mice carrying non-sarcomere mutations. Impaired relaxation was most frequently reported in mice models with *Tnnt2* (n = 25 [20.5%]), *Mybpc3* (n = 23 [18.9%]), and *Myh6* (n = 20 [16.4%]) mutations (Figure 4B). When a paper reports impaired relaxation, it often coincided with reporting of the presence (n = 50) or absence (n = 46) of cardiac hypertrophy. A total of 23 papers reported impaired relaxation in isolated cells, while 21 papers found no impairment. Two papers showed impaired relaxation in the mouse model but not in the isolated CMs.^71^^,^^72^ Rice et al^71^ is the only study in which no impaired relaxation was observed in both mouse and isolated cells. Three papers confirmed impaired relaxation in both in the animal model and isolated cells.^73^, ^74^, ^75^

Fibrosis is studied nearly as extensively (n = 143 of 304 [47%]) as cardiac relaxation with 78% (n = 112 of 143) of the papers reporting the presence of fibrosis, accompanied by either histological images or quantification, or both. Mice with a *Myh6* mutation (n = 38 of 51 [75%]) and *Mybpc3* (n = 17 of 23 [74%]) show fibrosis most often, while fibrosis was observed in only 56% (n = 25 of 45) of papers with other sarcomere mutation mouse models. The same holds true for *Tnni3* (n = 6 of 12 [50%]) and non-sarcomere mutations (n = 11 of 23 [48%]) such as *Actc1*, *Myl2*, and *Myl3* (Figure 4C). When it comes to assessing multiple hallmarks, we found 59 papers that study hypertrophy, impaired relaxation, and fibrosis, but only 25 papers report the presence of all 3 hallmarks in their model. These papers included mice with heterozygous mutations in *Tpm1* (n = 5), *Myh6* (n = 5), and *Tnnt2* (n = 2), described homozygous mutations in *Mybpc3* (n = 5), and included non-sarcomere genes (n = 3) (cysteine and glycine rich protein 3 [*Csrp3*]; synemin [*Synm*]; and obscurin, cytoskeletal calmodulin and titin-interacting RhoGEF [*Obscn*]).^76^, ^77^, ^78^

Sarcomere disarray, a structural characteristic of HCM, was investigated in 109 out of the 304 papers, comprising 36% of the total papers, and reported in 84 papers (77%). Mice with a *Myh6* (n = 30 of 37 [81%]) and *Tnnt2* (16 of 22 [72%]) mutation, together with mice with a non-sarcomere mutation (n = 14 of 18 [78%]) exhibited the highest incidence of disarray among the examined mouse models (Figure 4D). Among the studies included, 33 papers presented findings of sarcomere disarray, hypertrophy and fibrosis, while only 12 papers additionally showed impaired relaxation within their models (Table 1).

**Table 1:** Mouse Models With Main HCM Disease Characteristics

First Author, Year	Gene of Interest	Mutation	Zygosity	Cardiac Hypertrophy	Impaired Relaxation	Fibrosis	Sarcomere Disarray	CM Hypertrophy	Ventricular Arrhythmias
Geisterfer-Lowrance et al, 1996^18^	*Myh6*	R403Q	Heterozygous		x	x	x	x	
Vikstrom et al, 1996^88^	*Myh6*	R403Q		x		x	x	x	
Fatkin et al, 2000^90^	*Myh6*	R403Q	Heterozygous	x		x	x	x	
Freeman et al, 2001^58^	*Myh6*	R403Q	Heterozygous	x	x	x	x		
McConnell et al, 2001^85^	*Myh6*	R403Q	Heterozygous	x	x	x	x	x	x
Semsarian et al, 2001^316^	*Myh6*	R403Q	Heterozygous	x	x	x	x	x	
Wolf et al, 2005^317^	*Myh6*	R403Q	Heterozygous	x		x	x		x
Ma et al, 2021^318^	*Myh6*	R404Q	Heterozygous	x		x	x	x	
Jones et al, 1996^319^	*Myh6*	KO	Heterozygous	x	x	x		x	
McConnell et al, 2001^85^	*Mybpc3*	Neo	Heterozygous	x		x	x		x
Harris et al, 2002^320^	*Mybpc3*	KO	Homozygous	x	x	x	x		
Carrier et al, 2004^321^	*Mybpc3*	KO	Homozygous	x	x	x	x		
Toib et al, 2017^322^	*Mybpc3*	KO	Homozygous	x	x	x	x	x	
Li et al, 2020^241^	*Mybpc3*	KO	Homozygous	x	x	x	x		
Barefield et al, 2014^92^	*Mybpc3*	C'-terminal truncation	Homozygous	x	x	x	x		
Tardiff et al, 1999^75^	*Tnnt2*	R92Q			x	x	x	x	
Coppini et al, 2017^323^	*Tnnt2*	R92Q	Heterozygous	x	x	x	x		
Ferrantini et al, 2017^84^	*Tnnt2*	R92Q	Heterozygous	x	x	x			x
Ferrantini et al, 2017^84^	*Tnnt2*	E163R	Heterozygous	x	x	x			x
Tsoutsman et al, 2006^324^	*Tnni3*	G203S		x		x	x	x	
Tsoutsman et al, 2008^325^	*Tnni3/Myh6*	G203S/R403Q	Compound heterozygous	x		x	x	x	x
Muthuchamy et al, 1999^326^	*Tpm1*	D175N			x	x	x	x	
Prabhakar et al, 2001^327^	*Tpm1*	E180G	Heterozygous	x	x	x	x		
Prabhakar et al, 2003^328^	*Tpm1*	E180G	Heterozygous	x	x	x	x	x	
Schulz et al, 2013^329^	*Tpm1*	E180G			x	x	x	x	
Alves et al, 2014^330^	*Tpm1*	E180G	Heterozygous	x	x	x	x	x	
Song et al, 2011^114^	*Actc1*	E99K		x	x	x	x		
Lee et al, 2009^331^	*Myc*	Overexpression	Compound heterozygous	x	x	x	x	x	
Yuan et al, 2015^332^	*Myl2*	D166V		x	x	x	x		
Kazmierczak et al, 2013^333^	*Myl3*	A57G		x	x	x		x	
Sysa-Shah et al, 2012^334^	*ErbB2*	Overexpression	Heterozygous	x		x	x	x	
Liu et al, 2014^335^	*Plin1*	KO	Homozygous	x	x		x	x	
Xu et al, 2014^263^	*Pten*	KO	Homozygous	x	x	x		x	
Hunter et al, 1995^45^	*Ras*	KI	Homozygous	x	x		x	x	
Zheng et al, 2004^336^	*Ras/Myl2*	Constitutively active	Compound heterozygous	x	x	x		x	
Garcia-Pelagio et al, 2018^77^	*Synm*	KO	Homozygous	x	x	x		x	
Kimura et al, 2017^80^	*Vsp34*	KO	Homozygous	x	x		x	x	x

Selection of 35 publications that include 36 mouse models based on the reported presence of at least 4 hallmarks. An “x” indicates the presence of the hallmark.

CM = cardiomyocyte; HCM = hypertrophic cardiomyopathy.

CM hypertrophy has been studied in 72 out of 304 papers, accounting for 24.3% of the studies. Specifically, it is reported in 59 models independently, and in conjunction with cardiac hypertrophy in 43 models. A total of 33.9% reported CM hypertrophy in mice with a non-sarcomere mutation, while 57.6% focused on main sarcomere genes (*Myh6* [n = 15], *Mybpc3* [n = 9], *Tnni3* [n = 5], *Tpm1* [n = 4], and *Tnnt2* [n = 1]) (Figure 4E). Furthermore, a combination of cardiac hypertrophy, CM hypertrophy, and fibrosis was discussed in 28 papers. Among these papers, 13 included sarcomere disarray and only 6 papers showed all previously discussed HCM hallmarks (Table 1). CM hypertrophy is also studied in isolated ventricular cells and was reported to be present in 5 out of the 9 papers. No discrepancies in the presence or absence of CM hypertrophy were observed between ex vivo measurements and isolated ventricular cells.

Ventricular arrhythmias represent the least investigated hallmark of HCM, with only 23 out of 304 papers studying arrhythmias. In 24 mice models ventricular arrhythmias were reported, most frequently in models with sarcomere mutations: *Tnnt2* (n = 10 [41.7%]), *Myh6* (n = 4 [16.7%]), and *Mybpc3* (n = 3 [12.5%]) (Figure 4F). Ventricular arrhythmias were induced in 17 studies and occurred spontaneously in 7 studies, of which 3 studies reported on sarcomeric mutations (*Tnnt2* [F110I, I79N, R92Q] and *Mybpc3* [KI]) and 4 on nonsarcomeric mutations. When the type of arrhythmic patterns were specified, which was not done in all studies, premature ventricular contractions were reported most frequently. After challenging with a β-adrenergic agonist or pacing the occurrence of arrhythmia increased or sometimes progressed into ventricular tachycardia. Arrhythmias were detected in young animals of 80 days as well as in older animals up to 15 months.^79^^,^^80^ Isolated CMs from transgenic mice showed arrhythmic patterns in 3 studies. CMs with mutations in *Tnnt2* (R92Q) demonstrated spontaneous calcium waves and transients,^81^ burst contractions or extreme relaxation prolongations were found in *Mybpc3* KI CMs,^82^ and CMs showed arrhythmia after isoproterenol administration.^83^

One paper describes 2 experimental models that show impaired relaxation, hypertrophy, fibrosis, and ventricular arrhythmias. Both mouse models are male, are on a C57BL/6N background, and carry a *Tnnt2* mutation (E163R or R92Q).^84^ Out of all the 304 papers, only 1 reports all the HCM hallmarks in a male mouse model, carrying the R403Q mutation in *Myh6* on a 129SvEv background with an age of 30 to 50 weeks. Mice at the age of 10 to 20 weeks with the same genetic background showed all HCM hallmarks except for cardiac hypertrophy and ventricular arrhythmias.^85^

### Effects of zygosity, sex, and aging on HCM characteristics in mouse models

The effects of zygosity, and consequently the altered levels of endogenous or mutant protein, can significantly influence the characteristics of HCM. The impact of protein expression on HCM is evident in studies involving *Myh6* mutations, such as R453C and R719W. In 1-week-old homozygous mice, hypertrophy is observed, whereas in heterozygous mice, hypertrophy is absent at week 8 and only becomes evident at 26 weeks. This suggests that hypertrophy develops more slowly in heterozygous mice compared with homozygous ones.^86^ Similarly, Welikson et al^87^ reported no hypertrophy in ΔLCBD *Myh6* mice at 2 months, and at 10 months, hypertrophy occurred only in homozygous mice expressing 7% mutant protein, while heterozygous mice express 4% mutant protein. Both zygosity and the level of mutant protein expression are crucial factors. For example, Vikstrom et al^88^ demonstrated that mutant protein levels between 0.6% and 2.5% were insufficient to induce hypertrophy in 12-week-old mice, whereas levels of 10% to 12% did lead to hypertrophy. Additionally, homozygous R403Q mice experienced earlydeath, dying within 1 week, while heterozygous mice survived up to 9 months.^11^^,^^89^ In contrast, some studies have shown that neither heterozygous nor homozygous mutations induce HCM hallmarks. For instance, the V606M mutation in *Myh6*, as reported by Blankenburg et al,^86^ did not lead to hypertrophy or fibrosis. Fatkin et al^90^ observed that R403Q mice 0 to 6 days of age did not develop hypertrophy.^91^

Similar findings have been observed in *Mybpc3*-mutated mice. Barefield et al^92^ reported that 10- to 12-week-old homozygous C-terminal truncated *Mybpc3* mice, expressing 16% of the endogenous *Mybpc3* protein levels, exhibit hypertrophy and impaired relaxation, whereas heterozygous mice, expressing 64% of the endogenous *Mybpc3* protein, did not show these traits. The C-terminal truncated *Mybpc3* mice also developed fibrosis by 3 months and myocardial disarray and ventricular arrhythmias at 30 to 55 weeks, conditions that were absent in the heterozygous mice.^64^^,^^93^ Hypertrophy has been consistently reported in homozygous *Mybpc3 KO* mice but not in heterozygous mice.^62^, ^63^, ^64^^,^^82^^,^^94^, ^95^, ^96^ Impaired relaxation was similarly only observed in the homozygous mice.^13^^,^^62^^,^^63^ Additionally, solely homozygous KI mice were reported to exhibit ventricular arrythmias.^12^

No differences in zygosity, in combination with the presence of any HCM hallmarks, have been reported in the *Tnnt2* mutant mice, despite 5 studies reporting variations in *Tnnt2* protein and mutant expression. The R92Q mutant *Tnnt2* protein was expressed at levels ranging from 30% to 92% of total protein, with no differences shown between the lines.^75^^,^^97^ The latter is in line with the observation in human CMs, which showed increased myofilament Ca^2+^ sensitivity independent of mutant protein expression.^98^ However, zygosity-based differences were reported in other models, including mice with P1124L mutation in ryanodine receptor (*Ryr2*), miR-17-92 overexpression, the C58G mutation in Csrp3, PVEK KO in *Ttn*, and A8V in *Tnnc1.*^10^^,^^73^^,^^76^^,^^99^^,^^100^

Sex differences were studied in 20 studies. In R403Q *Myh6* mice, males showed hypertrophy at 2 months, while females did not, and this difference persisted at 7 to 40 weeks.^101^^,^^102^ However, Vikstrom et al^88^ found hypertrophy in female R403Q *Myh6* mice at 8 months but not in males. In the tTa x R403Q *Myh6* model, hypertrophy was present in males but absent in females.^61^ Impaired relaxation was noted in male R403Q mice at 4 and 10 to 12 months but not in females.^103^^,^^104^ Male R403Q mice developed hypertrophy and fibrosis at 15 weeks, while female exhibited these traits only at 30 to 31 weeks.^61^^,^^105^ Similarly, fibrosis was seen in male R403Q *Myh6* mice at 10 months but not in female mice.^106^ Interestingly, Bevilacqua et al^107^ found no sex differences in the R403Q model; however, the mice’s age was not reported, and a diet-inducing R403Q model showed no additional sex-related differences.^108^

Female mice with KI G>A in exon 6 of *Mybpc3* combined with exercise showed hypertrophy and impaired relaxation, unlike males.^109^ In the A8V *TnnC1* model, 3-month-old female mice showed impaired relaxation, while males did not.^110^ Male R58Q MYL2 mice had impaired relaxation at 7 months, but females did not.^111^ Tieg^–/–^ mice of both sexes did not show fibrosis at 4 months, but only in male mice hypertrophy and fibrosis was reported at 16 months.^112^

No sex differences were observed in several other models, including *Mybpc3* KO (heterozygous and homozygous), R146G *Tnni3*, E99K *Actc1*, N47K *Myl2*, S9A *Gsk3β*, and R4344Q *Obscn.*^62^^,^^78^^,^^106^^,^^111^^,^^113^^,^^114^

As mentioned previously, the distribution of age groups in studies involving *Myh6* mutations is consistent with the overall average across mouse studies, with more than half of the studies reporting ages between 0 and 6 months. The heterozygous R403Q mutation does not typically show hypertrophy before 8 weeks but does from 12 weeks onward.^11^^,^^85^^,^^103^^,^^115^, ^116^, ^117^ However, Georgakopoulos et al^118^ reported hypertrophy as early as 6 weeks, while Freeman et al^58^ found no hypertrophy at 9.65 months but did observe it at 10.55 and 10.65 months. Olsson et al^103^ reported hypertrophy at 4 months based on increased heart weight-to-body weight ratio, but this was not significant at 10 months, although differences in LV weight remained. Fibrosis and myocardial disarray were noted in males at 15 weeks and in females at 30 to 31 weeks.^105^ Other *Myh6* mutations, such as R453C and R719W, showed fibrosis at 26 weeks, which was not present yet 1 week after birth.^86^ Male mice with tTAxR403Q mutation exhibit hypertrophy from 20 weeks onward, though it was absent at 6 and 12 weeks.^61^ Additionally, homozygous ΔLCBD *Myh6* mice showed hypertrophy at 10 months but not at 2 months.^87^

On average, *Mybpc3* mice were younger (41% in the 0-3 months category compared with 28.6% in total mouse studies). A truncating mutation in *Mybpc3* led to hypertrophy at 14 to 16 months but not at 6 to 8 months.^119^ KO of *Mybpc3* resulted in hypertrophy at 10 to 11 months in heterozygous mice, with fibrosis also occurring at 10 to 11 months but not at 9 months.^62^ Friedrich et al^68^ reported hypertrophy at 13 weeks but its absence at 7 weeks, while Schlossarek et al^120^ observed hypertrophy between 2 and 13 weeks but not at birth. Homozygous mice did not show hypertrophy at 1 day old but did from 2 days onward.^96^ CM hypertrophy was present at 9 days and not at 2 days for KO mice (both heterozygous and homozygous).^96^ Homozygous KI *Mybpc3* exhibited hypertrophy from 3 days onward.^85^^,^^121^ Flenner et al^83^ reported impaired relaxation at 6 to 7 weeks and 22 to 25 weeks but not impaired relaxation at 14 to 17 weeks and 32 to 34 weeks in the G>A on the last nucleotide of exon 6 of *Mybcp3*, with hypertrophy also observed. Gedicke-Hornung et al^122^ found hypertrophy from 4 to 14 days but not at 55 to 57 days.^1^ McConnell et al^85^ noted that hypertrophy developed in mice 10 to 20 weeks of age and persisted through 30 to 50 weeks of age.

Guinto et al^123^ researched isolated CMs from *Tnnt2* mice with R92L and R92W mutations at 2 and 6 months of age. In both cases, impaired relaxation was reported. Mice with a R92L mutation showed hypertrophy but no impaired relaxation at 2 months, however both were observed in 6-month-old mice. R92W mice showed impaired relaxation and hypertrophy in both age groups.^124^ Mice with other mutations (R92Q, Δ160E, and truncating mutation) showed no hypertrophy.^75^^,^^125^^,^^126^ Impaired relaxation was only reported in 4- to 6-month-old mice with a R92Q or truncating mutation and in isolated CMs of Δ160E mice.

### Hamster models

The Syrian hamster, or *Mesocricetus auratus*, is another rodent model used to study HCM (n = 29). The Syrian hamster is a hereditary model of cardiomyopathy, although the genetic origin remains elusive. This model has been reported worldwide, including in Japan (n = 11), France (n = 7), Canada (n = 5), Italy (n = 3), the United States (n = 2), and Germany (n = 1). Various strains of the Syrian hamster were examined: BIO14.6 (n = 18),^33^^,^^34^^,^^36^^,^^37^^,^^127^, ^128^, ^129^, ^130^, ^131^, ^132^, ^133^, ^134^, ^135^, ^136^, ^137^, ^138^, ^139^, ^140^ UM-X7.1 (n = 7),^31^^,^^135^^,^^141^, ^142^, ^143^, ^144^, ^145^ CHF-146 (n = 3),^32^^,^^35^^,^^146^ BIO 8262 (n = 1),^147^ and J2N-k (n = 1).^147^ Male and female animals were studied in 5 publications,^133^^,^^134^^,^^140^, ^141^, ^142^ solely male animals in 10 studies,^32^^,^^33^^,^^35^^,^^127^, ^128^, ^129^, ^130^, ^131^^,^^135^^,^^136^^,^^146^ and sex was not specified in 14 out of the 29 studies.^31^^,^^34^^,^^36^^,^^37^^,^^130^^,^^132^^,^^137^^,^^139^^,^^143^, ^144^, ^145^^,^^147^^,^^148^ The ages studied varied from 2 weeks to 1 year, with 80% of the studies focusing on hamsters 0 to 6 months of age (n = 13, 0-3 months of age; n = 19, 4-6 months of age) and only a few older than 6 months (n = 6, 7-9 months of age; n = 2, 10-12 months of age).

The HCM hallmarks—hypertrophy (n = 15), impaired relaxation (n = 6), fibrosis (n = 6), CM hypertrophy (n = 5), and myocardial or sarcomeric disarray (n = 2)—were studied in the hamster models. In the BIO14.6 strain, hypertrophy was reported once in 6-week-old hamsters^128^ and once at 90 and 160 days.^33^ Overall, cardiac hypertrophy was most often detected at 6 months of age in the BIO14.6 studies. D’hahan et al^127^ showed no hypertrophy in BIO14.6 hamsters at 30, 65, and 140 days, but hypertrophy was present at 220 and 350 days. At 220 days, in addition to hypertrophy, increased levels of cytosolic calcium associated with impaired relaxation was found in isolated CMs.^127^ Impaired relaxation and hypertrophy were also present at 6 months of age in papillary muscles of hamsters from the BIO14.6 strain when compared with the healthy F1B strain.^138^, ^139^, ^140^ In line with these findings, no impaired relaxation was found in BIO14.6 hamsters 14 to 20 weeks of age.^37^ Impaired relaxation and hypertrophy were not present at 50 days of age in the UM-X7.1 strain^31^ but were demonstrated after 80 days.^31^^,^^142^ In 30-day-old UM-X7.1, sarcomere disarray was found, but fibrosis was only reported after 90 days.^144^ In the BIO8262 strain, no CM hypertrophy was demonstrated after 2 weeks, but it was present at 6 weeks of age.^147^ In this model, disarray was already present after 42 days.^147^

### Rat models

We identified 25 studies with a rat (*Rattus norvegicus*) HCM model, which were either induced or spontaneous. Models carried a sarcomere gene mutation *Myh7* (KO, n = 1),^149^ *Mybpc3* (W1098X, n = 1),^150^ *Tnnt2* (F72L, Del ex16, and del; n = 4),^151^, ^152^, ^153^, ^154^ and *Tpm1* (D175N, E180G, n = 2).^155^^,^^156^ Two studies examined the “hypertrophic heart rat,” which is an inherited model of HCM of which the genetic origin remains elusive,^157^^,^^158^ and 5 studies used a spontaneous HCM model.^38^, ^39^, ^40^, ^41^^,^^159^ In hypertrophic heart rat, hypertrophy could not be demonstrated at 2 weeks but was visible at the age of 4 weeks.^160^ Sprague Dawley rats were used in 6 studies,^20^^,^^149^^,^^151^, ^152^, ^153^, ^154^, ^155^ the WKYxNCRj strain was used in 4 studies,^38^, ^39^, ^40^, ^41^ and SS/Mcwi was used in 1 study.^159^ The strain was not described in 4 studies.^150^^,^^156^, ^157^, ^158^ Female rats were used in 1 study,^155^ male rats were used in 4 studies,^40^^,^^150^, ^151^, ^152^ and male and female rats were used in 3 studies,^38^^,^^39^^,^^157^ while sex was not specified in 7 studies.^41^^,^^150^^,^^153^^,^^154^^,^^156^^,^^158^^,^^159^ Similar to the hamsters, the ages studied varied from 2 weeks to more than 1 year, with 68% of the studies focusing on rats 0 to 6 months of age (n = 6, 0-3 months of age; n = 7, 4-6 months of age) and only a few older than 6 months (n = 2, 7-9 months of age; n = 1, 10-12 months of age; n = 1, 12+ months of age).

The thick filament models involved KO models on a Sprague Dawley background. In the *Myh7* KO model, no fibrosis or disarray was visible after 12 weeks but was observed at 30 weeks.^149^ Cardiac hypertrophy, however, was present at both 12 and 30 weeks. In addition, isolated ventricular CMs showed impaired relaxation. These findings were similar for heterozygous and homozygous KO animals.^149^ *Mybpc3* W1098X did not cause hypertrophy in heterozygous animals at 6 months of age but did result in hypertrophy, impaired relaxation, and fibrosis in homozygous animals.^150^

The thin filament models involved transgenic overexpression of mutant genes. In animals 12 to 14 weeks of age with pathogenic D175N and E180G *Tpm1* mutations, no diastolic dysfunction, hypertrophy, or fibrosis was detected. However, disarray was demonstrated at this age.^155^ Arrhythmias were not detected in D175N, even after exercise by running,^156^ while arrhythmias were present in E180G mice at baseline and after exercise.^156^ Exercise as a second disease hit also triggered arrhythmias and disarray in a rat model with *Tnnt2* deletion of exon 16, a model based on an intron 15 splice donor site mutation in human HCM patients.^152^

### Rabbit models

HCM in rabbits was studied in 10 publications, in which transgenic rabbits were used in 9 papers. Seven out of 10 studies were from the same research group with the same rabbit model.^46^^,^^160^, ^161^, ^162^, ^163^, ^164^, ^165^ Breed was only specified in 1 study, in which they used New Zealand White rabbits.^165^ The most commonly studied mutation is R403Q *MYH7* (n = 8)^166^^,^^167^ and 1 study examined the R146G *TNNI3* mutation.^168^ A study in 59 rabbits with cardiac disease found 2 cases of HCM but did not analyze the genetic cause.^169^ Transgenic rabbits 1 to 6 months of age with the R403Q *MYH7* mutation showed impaired relaxation and disarray, but did not demonstrate hypertrophy, CM enlargement or fibrosis.^162^ Rabbits 7 to 30 months of age did show hypertrophy, enlarged CMs, and fibrosis.^162^ Hypertrophy of the heart and CM hypertrophy were also demonstrated in other studies, and was found in animals up to 3 years of age.^160^^,^^161^^,^^163^, ^164^, ^165^^,^^167^ In addition, fibrosis was observed in other long-term studies.^46^^,^^160^^,^^163^^,^^165^ Increased vulnerability to ventricular arrhythmias was demonstrated in R403Q rabbits at 2 and 3 years of age.^160^^,^^164^

Rabbits with a R146G *TNNI3* mutation were also studied at different time points; the animals did not show hypertrophy at 6 months of age, but did demonstrate fibrosis at 2 years of age, and presented with impaired relaxation without arrhythmias at 21 months of age.^168^ While repolarization was prolonged, calcium kinetics in isolated CMs were not different from nontransgenic CMs.^168^

### Cat models

**Figure 5:**
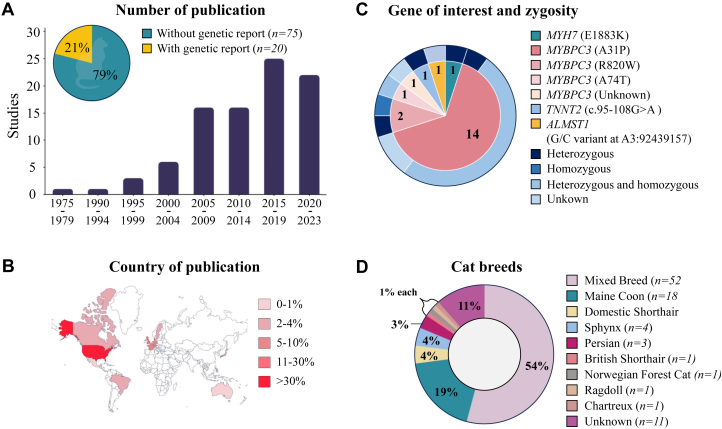
General Information on Cat Studies (A) The numbers of cat studies published per 5-year period. The genetic background was either reported (yellow) or not reported (green). (B) Geographical distribution of research on cat models based on the country of corresponding author. Each country is shaded according to the percentage of total cat model publications. (C) Overview of the cat breeds, the number of studies using specific breeds. (D) Distribution of gene mutations, each segment of the inner circle represents the percentage of studies on a specific gene. The outer circle categorizes these specific mutation based on zygosity.

HCM is the most common form of feline cardiomyopathy and is estimated to affect up to 15% of domestic cats (*Felis catus*).^170^^,^^171^ We identified 95 studies investigating HCM in cats (Figure 5A; Supplemental References). The majority of studies comes from the United States (Figure 5B). Retrospective studies, prospective studies, and case reports describing HCM in domestic cats mostly involved client-owned, breeder-owned, or shelter cats (66% [n = 63 of 95]). Only 7 (7.4%) studies used cats based in a research colony. In 60% of the studies, HCM parameters were investigated in a mixed pool of breeds that we refer to as mixed breed (Figure 5D). The most frequent investigated feline breeds involve Maine Coon (n = 19) and Domestic Shorthair (DSH) (n = 5). Most studies included both male and female cats (n = 79). Out of 95 papers, only 11 conducted age-matched studies. Ten papers reported on kittens or junior cats (10.5%, average age between 0 and 2 years), 38 papers used adult cats (40.0%, average age between 3 and 6 years), 23 papers focused on mature cats (24.2%, average age 7+ years), and in 23 papers, only a range of ages was provided.

We found that 21% of all studies documented the genetic cause in their feline patients. An overview of the HCM-associated gene variants is shown in Figure 5C. The A31P mutation in *MYBPC3* is the first discovered and most commonly investigated gene variant in Maine Coon cats, with 14 publications evaluating the cardiac phenotype in heterozygous and homozygous carriers (Figure 5D). It is estimated that the prevalence of this variant in Maine Coon cats is between 35% and 42%, of which 90% are heterozygotes.^171^, ^172^, ^173^ While the A31P mutation is not reported in humans, the clinical phenotype observed in the Maine Coon cats resembles the human situation, with a more severe phenotype (characterized by hypertrophy and impaired relaxation) in homozygotes, and a less severe, later onset of disease in the heterozygotes.^174^, ^175^, ^176^ R820W is another *MYBPC3* variant that has been associated with HCM in Ragdoll cats.^177^^,^^178^ Homozygous cats developed a HCM phenotype 18 months earlier than the heterozygous carriers.^177^ Notably, this gene variant was later documented in humans in which a homozygous patient developed HCM, while the heterozygotes had no to mild clinical expression.^179^ As the homozygous Maine Coon and Ragdoll cats are at a higher risk of developing HCM, the American College of Veterinary Internal Medicine has recommended genetic testing for A31P and R820W *MYBPC3* variants in these breeds and emphasized that homozygous cats should not be used for breeding purposes.^171^ Similar to humans, the epigenetic factors and the genotype-phenotype heterogeneity in heterozygotes carrying these 2 mutations are still unclear and need to be further studied. Recently, variants in *MYH7* and *TNNT2* have also been associated with HCM in DSH and Maine Coon cats, respectively. DSH cats carrying the heterozygous E1883K variant in *MYH7*, a variant present in humans, showed impaired relaxation, cardiac hypertrophy, fibrosis, and myocardial disarray.^180^ The reported human case involved a homozygous carrier experiencing HCM and myosin storage myopathy.^181^ Because of the limited number of reported humans carrying this variant, the causality of this variant in humans is of uncertain significance (ClinVar). Maine Coon cats with the homozygous *TNNT2* variant c.95-108G>A showed cardiac hypertrophy, while the heterozygous parental cats were unaffected.^182^ There is no report of this *TNNT2* variant in humans. Variants in *ALMST1*, which encodes a centrosome and basal body-associated protein, have been related to HCM in Sphynx cats. Sphynx cats with a heterozygous or homozygous variant in *ALMST1* showed intraventricular septal hypertrophy, fibrosis, and myofiber disarray. Affected cats were between 1 and 14 years of age.^183^ *ALMS1* mutations can lead to Alström syndrome in humans, in which dilated cardiomyopathy is reported in infants and restrictive cardiomyopathy is reported in adults. There are no reports of HCM associated with mutations in this gene.^183^^,^^184^

**Figure 6:**
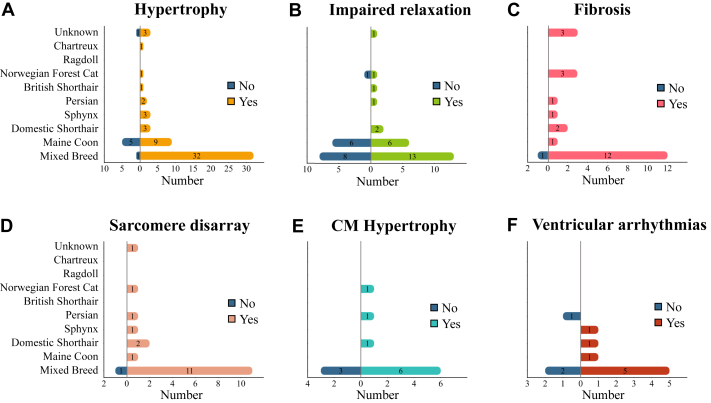
HCM Hallmarks in Cat Models (A-F) The numbers are based on the extracted data related to HCM hallmarks including hypertrophy, impaired relaxation, fibrosis, sarcomere disarray, CM hypertrophy, and ventricular arrhythmias. An HCM hallmark is identified as “yes” when a significant difference between HCM model and control model is observed. If no significant difference is found, the HCM hallmark is marked as “no,” indicating that it was investigated but not identified. A single study may investigate multiple breeds allowing it to contribute to multiple breeds. Abbreviations as in Figure 4.

Echocardiography and tissue Doppler measurements are the gold standards in measuring impaired relaxation and hypertrophy in cats, while cardiac magnetic resonance measurements to detect fibrosis have been challenging, which explains the limited data for this parameter.^170^ Hypertrophy (n = 56 of 95 [58.9%]) is the most extensively studied HCM hallmark, with 51 of those papers reporting hypertrophy in at least 1 of the cat models (Figure 6A). Impaired relaxation emerges as the second most studied HCM hallmark in cats (n = 37 of 95 [38.9%]) and was observed in 23 (62.2%) papers. The reported differences in relaxation are driven by the size of the left atrium (LA) or left atrial-to-aortic ratio (LA:Ao), rather than by Doppler measurements (Figure 6B). Out of the 56 papers that investigate hypertrophy, 29 also examine relaxation. When fibrosis is studied (n = 22 of 95 [23.2%]), the presence of fibrosis in HCM cats is reported except for 1 paper. The presence of fibrosis was reported in 6 papers (only 1 with statistical testing), 10 provided histological images, and 5 included both images and quantification (Figure 6C). Sarcomere disarray is studied in 19 papers, showing a trend similar to that of fibrosis across cat models. This is largely due to the significant overlap, with 17 papers reporting results on both fibrosis and disarray, indicating a 100% correlation. Only 5 studies focus exclusively on the presence of fibrosis, and 2 studies report solely on disarray (Figure 6D). CM hypertrophy was explored in 12 (12.6%) papers, with 9 of these reporting its occurrence (Figure 6E). Two papers observed no increase in cell surface area but did report ventricular hypertrophy in the tissue.^185^^,^^186^ Research on ventricular arrhythmias was covered in 9 studies (Figure 6F). Trehiou-Sechi et al^187^ examined arrhythmias across different cat breeds and found them only in Persian cats, with no arrhythmias detected in the Maine Coon, Sphynx, and DSH.

### Pig models

HCM has also been studied in large animal models such as *Sus scrofa domesticus*, or pigs. Corresponding authors of 7 publications originated from Taiwan, 1 from China, 1 from the United States, and 1 from Germany. The spontaneous occurrence of HCM was studied in 7 out of 10 publications,^43^^,^^188^, ^189^, ^190^, ^191^, ^192^, ^193^ and 3 studies used transgenic pigs.^27^^,^^194^^,^^195^ Landrace pigs were studied most often (n = 6),^188^, ^189^, ^190^, ^191^, ^192^^,^^194^ while 2 other studies used mixed breeds.^43^^,^^193^ One study used Yucatan miniature pigs^194^ and 1 study did not specify the breed.^27^ Hypertrophy and fibrosis were demonstrated in animals with spontaneous HCM in 6 and 5 studies, respectively, in which various ages between 5 and 21 months were investigated.^43^^,^^188^, ^189^, ^190^, ^191^, ^192^ Disarray was observed in the hearts of animals 5 to 16 months of age.^34^^,^^43^ In the transgenic animals, the pathogenic R723G and R403Q mutations in *MYH7* and R495Q in *MYBPC3* were studied.^27^^,^^194^^,^^195^ Pigs with a mutation in *MYBPC3* showed fibrosis after 10 days.^27^ Yucatan miniature pigs with the R403Q *MYH7* mutation did not show hypertrophy, but did demonstrate fibrosis and disarray at 3 months of age.^195^ Relaxation deficits, CM hypertrophy, and arrhythmia incidence were not studied in the pig models.

### Monkey models

Nonhuman primates have been used because of their genetic and physiological similarities to human. We included 8 publications where HCM was the topic of research in nonhuman primates, sometimes called “occult” or general left ventricular hypertrophy (LVH). All studies were published between 2013 and 2020, all corresponding authors from the included publications were from the United States, and almost all articles were from the same research institute (n = 7 of 8 [87%]). Rhesus macaques (*Macaca mulatta*) were studied most frequently (n = 7 of 8 [87%]),^196^, ^197^, ^198^, ^199^, ^200^, ^201^, ^202^ and 1 study (n = 1 of 8 [13%]) investigated night monkeys (*Aotus nancymaae*, *A vociferans*, and *A lemurinus griseimembra*).^50^ One study reported the genotype of the animals and found an *MYBPC3* mutation on chromosome 14, of which the haplotype significantly associated with LVH,^198^ and the other studies reported on spontaneous occurrence of HCM. The age of the animals was reported in 6 (75%) out of 8 studies, with ages ranging from 0 to 30 years of age. Sex was reported in all but 1 study. Six publications reported the use of control animals, the other studies only studied HCM animals.^197^^,^^198^ For the studies that did include control animals, various terms were used to indicate the control groups, such as “normal,” “clinically healthy,” or “unaffected.” Most studies classified control animals as healthy and/or free of cardiac disease based on echocardiographic data (n = 4 of 6 [67%]). In 4 studies, LVH and impaired relaxation based on echocardiographic data were part of the inclusion criteria for the HCM animals, though was not explicitly measured. Hypertrophy was investigated in 4 of 8 studies,^50^^,^^199^, ^200^, ^201^ cardiac relaxation was investigated in 3 of 8 studies,^50^^,^^200^^,^^201^ only 1 study performed studies at microscopic/cellular level to examine CM hypertrophy, disarray, and fibrosis,^199^ and 1 study investigated ventricular arrhythmia via electrocardiography analysis.^200^ Definitive claims on hypertrophy or diastolic dysfunction were complicated by the frequent occurrence of hypertrophy in clinically normal night monkeys^50^ and the age-related diastolic dysfunction in geriatric (>16 years of age) rhesus macaques,^202^ respectively. None of the included studies demonstrated fibrosis, ventricular arrhythmia, or disarray in nonhuman primates with HCM.

### Fruit fly models

The fruit fly, or *Drosophila melanogaster*, has the potential to serve as a genetic HCM model. The majority of the papers originate from the United States, while Germany, China, and France each contributed 1 paper. We identified 12 studies, of which 1 investigated the role of receptor tyrosine kinase signaling in development of cardiac hypertrophy,^203^ and 11 reported on mutations in fly equivalent sarcomere genes: myomesin (*MYOM2*) (n = 2),^204^^,^^205^ *MYH7* (n = 4),^206^, ^207^, ^208^, ^209^ *MYL2* (n = 1),^18^ *MYBPC3* (n = 2),^47^^,^^210^ and *ACTC1* (n = 2).^211^^,^^212^ The ages studied ranged from 2 hours to 7 weeks. Male and female flies were examined in 3 studies, while solely female flies were investigated in 8 studies. Sex was not specified in 1 study.^18^

Impaired relaxation was found in flies with a heterozygous K146N *MYH7* mutation,^208^ A295S *ACTC1* mutation,^212^ and W1118 *MYOM2* mutation in the fly equivalent of myomesin,^205^ at 1, 1, and 3 weeks, respectively. Flies with a heterozygous K146N *MYH7* mutation showed no eccentric hypertrophy and no CM enlargement but did display disarray after 1 and 3 weeks of age.^208^ Flies with the homozygous K146N *MYH7* did not show disarray after 2 hours, but disarray was already visible after 2 days.^208^ Furthermore, disarray was demonstrated in flies with C-terminus truncating *MYBPC3* after 15 days,^47^ and a *MYOM2* mutation after 21 days.^205^ Moreover, flies with a W1118 *MYOM2* mutation presented with arrhythmias, which could be ameliorated by exercise.^205^ Fibrosis was not studied in any of the fly models.

### Zebrafish models

The zebrafish, or *Danio rerio*, has proven to be a suitable model of human disease because of the high homology of genes compared with humans.^213^ Here, we included a total of 14 studies that reported genetic models of HCM. Of these 14 studies, 6 looked into sarcomere mutations,^49^^,^^214^, ^215^, ^216^, ^217^, ^218^ while 8 studies investigated nonsarcomere mutations.^19^^,^^21^^,^^26^^,^^28^^,^^219^, ^220^, ^221^ The ages of animals varied from 24 hours postfertilization to 1 year, while sex was not mentioned in any of the studies. The strain was specified in 5 out of 14 studies and levels of the mutant messenger RNA or protein were reported in 3 papers. The sarcomere gene *Mybpc3* was most often the gene of interest (n = 3 of 14 [21%]),^214^^,^^215^^,^^217^ other sarcomere genes included *Tnnt2*,^49^ *Myh7*,^216^ and *Myl3.*^218^ Non-sarcomere genes that were studied included several metabolic genes such as *Gtpbp3* and *Mto1*, both involved in mitochondrial transfer RNA modification,^220^^,^^221^ *Fastkd2*, a mitochondrial RNA-binding protein,^26^ and *Ndufa7*, part of complex I of the mitochondrial respiratory chain.^21^ One study investigated *Wtip*, involved in non-canonical Wnt signaling.^28^

Fish with *Mybpc3* mutations (c.772+1G>A, c.654+4dupTGG, R820Q, and heterozygous KO) show hypertrophy at 72 hours postfertilization, while hypertrophy could not be demonstrated for the V762D *Mybpc3* mutation at the same age.^214^^,^^215^^,^^217^ Disarray also occurs at this age in fish with *Mybpc3* point mutations.^214^ Fish at 3 or 6 months with heterozygous or homozygous KO of *Mybpc3* showed hypertrophy, CM hypertrophy, fibrosis, and disarray, although it is not clear whether the characteristics are attributed to the 3- or 6-month-old fish.^215^ The human *Tnnt2* gene variant with a mutation in the splice donor site of exon 15, causing a truncated cardiac troponin T protein, did not cause CM hypertrophy but did cause disarray at 4 days postfertilization in the zebrafish heart. However, this disarray disappeared after 21 days postfertilization.^49^ Fish with a *Myh7* (L655M) mutation showed no hypertrophy at day 3 and 4 postfertilization, but it was observed after 5 days.^216^ A 1-year-old fish with a mutation in the non-sarcomere GTP-binding protein 3 displayed CM hypertrophy and fiber disarray.^220^ CM hypertrophy was also observed in fish of 6 months with heterozygous and homozygous KO of the mitochondrial *Mto1*.^221^ Impaired relaxation was observed in Y233F *Wtip* fish 48 hours postfertilization.^28^ Arrhythmias were not described in any of the included studies.

### Miscellaneous animal models

Three studies were published on the spontaneous occurrence of HCM in dogs. In 1 study, the hearts of dogs of mixed breeds showed hypertrophy, disarray, and signs of fibrosis.^5^ Age was not defined in this cohort. In a second study, dogs of different breeds and ages were described to have septal hypertrophy, though this was not shown in the publication.^222^ As the study searched for the occurrence of HCM across a larger population, no control group was defined either. In a more recent study, dogs of mixed breeds ∼5.5 years of age showed LVH and reported impaired relaxation.^223^

The Japanese rice fish, *Oryzias latipes*, also known as the medaka, was used to study the effects of a missense mutation in an immunoglobulin domain located in the M-line–A-band transition zone of titin.^51^ Hypertrophy was not evident in heterozygous fish at 3 days postfertilization, but was present in homozygous fish. Homozygous fish are also described to present diastolic dysfunction. Both heterozygous and homozygous fish further demonstrated disarray.^51^

The Australian red kangaroo, *Osphranter rufus* (formerly known as *Macropus rufus* as mentioned in the paper), was subjected to echo- and electrocardiographic measurements in a small study with 7 animals. Animals of 1.5 to 5 years of age showed cardiac hypertrophy and impaired relaxation, comparable to human patients with nonobstructive HCM.^44^ No control kangaroo values were used in this study. Although the animals showed symptoms of HCM, no obvious etiology could be attributed to the findings in the red kangaroo.^44^

The *Llama pacos*, or alpaca, was investigated in a case study. The animal of interest was 3 years of age and died unexpectedly. Histological analysis demonstrated hypertrophy of the heart, with enlarged CMs and disarray, but with minimal fibrosis.^48^

### Fibrosis type

We did not specify the exact type of fibrosis due to inconsistent reporting across studies, we believe that it is important to highlight both the presence of fibrosis and the possibility that different types of fibrosis may arise from distinct pathomechanisms. This distinction could be significant for understanding the variability in fibrosis presentation across models and its implications for disease progression. For example, in a study by Biasato et al,^224^ histopathological analysis of 21 cat hearts diagnosed or suspected of HCM revealed that 67% (n = 14 of 21) exhibited fibrosis, with 24% (n = 5 of 21) showing replacement fibrosis, 24% (n = 5 of 21) displaying myocardial interstitial fibrosis, and 18% (n = 4 of 21) showing endocardial fibrosis. Variability in fibrosis types is also observed in mice. For example, in *Myh6* (R403Q) mice, interstitial fibrosis is reported in young mice, while replacement fibrosis appears in older mice.^105^ However, even within the same study, the presence of fibrosis is not always consistent. In 1 study, only 3 out of 5 *Mybpc3* KO mice exhibited foci of interstitial fibrosis.^63^ Additionally, in 1 study the degree of fibrosis correlated with the expression levels of the mutant protein.^75^

### Cell-based models

Various cell-based models have been used as an alternative to animal models to study HCM. The relative ease of cell culturing compared with animal housing, have made these models a popular choice among researchers. Moreover, the recent advances in human-based cell models (hiPSC technology) have grown much interest as they could potentially fill the gap between the cardiac physiological differences of animals and humans. In this review, we focused on information regarding the origin of the cells (species), whether they are engineered or patient-derived, the platform used for structural and functional analyses (2-dimensional, 3-dimensional), and the age of the cells in which experiments were performed. We report the registered data related to the HCM characteristics referring to impaired relaxation, CM hypertrophy, proarrhythmic behavior, and sarcomere disarray.

### Miscellaneous cell types

We included 30 studies in which isolated cells or cell lines were used to transfect (likely) pathogenic mutations to analyze HCM in vitro. Of these studies, 17 analyzed sarcomere mutations^12^^,^^18^^,^^20^^,^^25^^,^^27^^,^^57^^,^^167^^,^^225^, ^226^, ^227^, ^228^, ^229^, ^230^, ^231^, ^232^, ^233^, ^234^ and 12 focused on non-sarcomere mutations.^9^^,^^10^^,^^14^, ^15^, ^16^, ^17^^,^^21^^,^^22^^,^^26^^,^^28^^,^^29^^,^^235^ The cells used in these studies originated from rat (n = 18),^9^^,^^12^^,^^14^, ^15^, ^16^^,^^18^^,^^20^, ^21^, ^22^^,^^25^^,^^28^, ^29^, ^30^^,^^225^^,^^226^^,^^228^^,^^229^^,^^234^ mouse (n = 6),^9^^,^^17^^,^^27^^,^^57^^,^^227^^,^^232^ guinea pig (n = 1),^231^ chicken (n = 1),^233^ and cat (n = 1),^166^ and were either isolated or commonly used cell lines. We also included studies on human cell lines and fibroblasts (HEK and AC-16) (n = 5).^9^^,^^10^^,^^26^^,^^230^^,^^235^ General information on isolated cells from animals was inconsistent reported. Strain or breed was not mentioned in 6 papers (35%), and sex was not described in 12 (71%) papers. Three papers used only females^12^^,^^226^^,^^229^ and 2 papers used only males.^22^^,^^231^ Seven papers described the age in days specific, while 11 papers described the age by “adult” (n = 7) or “neonatal”/”embryo” (n = 4).

Relaxation was not slowed in embryonic stage 26 to 30 chicken CMs with R403Q, R453C, and G584R *MYH7* mutations, although these cells did show sarcomere disarray.^233^ The D778G and G741R *MYH7* mutations expressed in myotubes did not result in prolonged relaxation.^57^ Sarcomere disarray for the R403Q *MYH7* mutation was also demonstrated in transfected adult cat CMs after 120 hours.^167^

The pathogenic *TNNT2* R92Q and *TNNI3* R145G mutations prolonged relaxation in transfected adult LV guinea pig CMs,^231^ as did the *TPM1* A63V mutation induced in female adult rat CMs.^12^^,^^226^^,^^229^ The *TNNI3* R146G mutation, also in combination with *TPM1* A63V mutation, showed prolonged relaxation time in adult female rat CMs.^226^^,^^234^ The *TNNI3* R193H mutation, alone or in combination with *TNNC1* G159D, showed prolonged relaxation time compared with controls and the *TNNC1* G159D mutation.^226^ The calcium transient amplitude was decreased in HL-1 cells at 48 and 72 hours of transfection with E62Q and M281T *TPM1* mutations.^227^ Mutations in ankyrin repeat domain 1 (*ANKRD1*) (P52A, T123M, I280V) transduced in neonatal rat CMs did not show evidence for impaired relaxation,^225^ and myopalladin (*MYPN*) Y20C mutation in rat CMs did not result in sarcomere disarray.^20^

The P1124L mutation in the gene encoding *RYR2* caused calcium oscillations in transfected HEK cells.^10^ CM hypertrophy was also observed in neonatal rat ventricular CMs with a Y233F mutation in the non-sarcomere *WTIP*, involved in Wnt signaling.^28^ Lu et al^17^ showed that overexpression of *MEOX1* in HL-1 cells induced hypertrophy, whereas knockdown of *MEOX1* showed no differences compared with control. Similarly, overexpression of *YAP2* in H9c2 cells induced hypertrophy, while knockdown of *YAP2* in neonatal rat CMs showed no increase in cell size.^29^

### ESC*-*derived models

We identified 10 studies, of which 6 included sarcomere mutations and 4 included non-sarcomere mutations. Homozygous mutations were most often studied, with only 3 studies including heterozygous mutations.^6^^,^^7^^,^^236^ Two studies created ESCs, and did not make ESC-CM lines, with either a *MYH7* KO^237^ or junctophilin-2 (*JPH2*) KO.^238^ Nine out of 10 studies used human cells, while 1 studied mouse ESCs.^52^ Isogenic controls were created in 3 studies^6^, ^7^, ^8^ and the sex of the cells was defined in only 1 study.^238^

Impaired relaxation and cellular hypertrophy was observed in cells with R92Q *TNNT2*^236^ and c.2905+1G>A *MYBPC3* mutations,^239^ while ESC-CMs with the R453C *MYH7* mutation did not show impaired relaxation but did demonstrate hypertrophy.^7^ Cellular arrhythmia, as evident from delayed afterdepolarizations, early afterdepolarizations, triggered beats, increased beat-rate variability, and aberrant calcium transients was present in ESC-CM lines with mutations in *TNNT2* R92Q, *MYBPC3* c.2905+1G>A, and *MYH7* R453C^7^^,^^236^^,^^239^ but not in the *MYL2* R58Q cell line.^52^

Impaired relaxation and cellular hypertrophy was reported in lines with KO of the non-sarcomere genes *MLP*^240^ and *RAD*.^241^ Proarrhythmic behavior of cells was demonstrated in lines with KO of *RAD*^241^ and *ALPK3* W1264X mutation.^8^ The ages of the cells in all studies varied, and age-dependent changes were observed in several studies. ESC-CMs with *MLP* KO showed no impaired relaxation or hypertrophy at 15 days post-differentiation but did show both characteristics after 22 and 30 days.^240^ Age-dependent effects were also found in the ESC-CM line with a *RAD* KO. After 20 days no arrhythmic events were detected, but these were present at 30 and 40 days post-differentiation.^241^ Sarcomere disarray was demonstrated in 6 studies.^7^^,^^8^^,^^52^^,^^239^, ^240^, ^241^

### hiPSC-derived models

**Figure 7:**
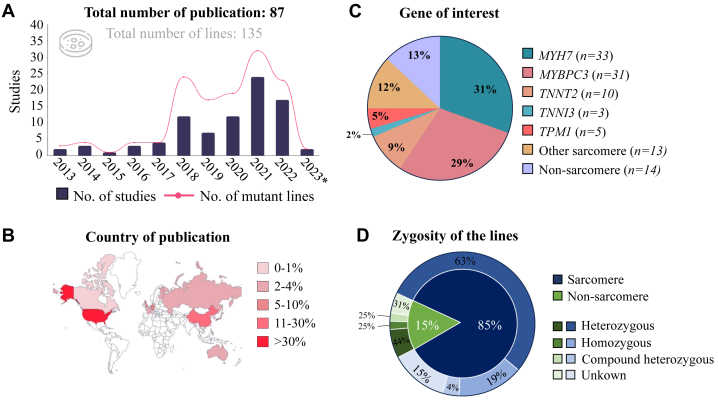
General Information on hiPSC-CM Studies (A) The numbers of human induced pluripotent stem cell (hiPSC)/human induced pluripotent stem cell–derived cardiomyocyte (hiPSC-CM) studies, including reports on the generation of hiPSCs, and generated mutant lines published. ∗Until April. (B) Geographical distribution of research on hiPSC/hiPSC-CM studies based on the country of corresponding author. Each country is shaded according to the percentage of total cat model publications. (C) Distribution of gene mutations, each segment represents percentage of studies investigating a specific gene. All genes that translate to sarcomere proteins are grouped as “other-sarcomere” except for *MYH7*, *MYBPC3*, *TNNT2*, *TNNI3*, and *TPM1*. Genes not translating to sarcomere proteins are grouped as “non-sarcomere.” (D) Overview of zygosity, categorization of hiPSC models based on their zygosity in relation to sarcomere mutations, non-sarcomere mutations, and compound heterozygous mutations.

Soon after the successful differentiation of hiPSCs to CMs,^242^^,^^243^ the first studies investigating the effect of HCM-causing mutations on hiPSC-CMs phenotype and evaluating the effect of different compounds on hiPSC-CMs carrying HCM mutations emerged.^53^^,^^54^ As this model recapitulates the genetics of the individual, patient-derived lines have been generated to study the genotype-phenotype association.^244^ Furthermore, with the invention of gene editing tools such as CRISPR/Cas9, engineered lines have been created to study mutation-specific effects on a similar genetic background. A total of 87 HCM hiPSC/hiPSC-CM studies, including 29 reports on the generation of hiPSC, were published worldwide between 2013 and 2023 (Figures 7A and 7B, Central Illustration, Supplemental References). The publications examined various HCM mutations, with 26 papers focusing on multiple hiPSC lines carrying different HCM mutations. A total of 132 hiPSC lines were identified; however, it is unclear whether the same cell lines were used across multiple publications. Out of 132 hiPSC lines, 88 (66.7%) are identified as pathogenic or likely pathogenic, or conflicting data are reported (according to ClinVar). In ∼70% of genotype-positive HCM patients, a pathogenic gene variant is identified in *MYH7* and *MYBPC3*,^244^ which corresponds with the relatively high percentage (62.9%) of hiPSC lines carrying a *MYH7* or *MYBPC3* mutation (43 and 40 lines in 33 and 31 papers, respectively) (Figure 7C). In accordance with the mouse models, the *MYH7* R403Q is the most studied mutation (n = 9). Twenty-one other MYH7 mutations were reported across multiple papers including R663H (n = 5), R723C (n = 4), E848G (n = 3), R453C (n = 3), R719W (n = 2), and V606M (n = 2). The most frequently described *MYBPC3* mutations were W792Vfs (n = 4), KO (n = 3), L460Wfs (n = 3), R502W (n = 3), Q1061X (n = 3), R943X (n = 2), and V321M (n = 2). Within the *TNNT2* gene, R92Q (n = 3) and I79N (n = 2) mutations were described in multiple papers.

**Figure 8:**
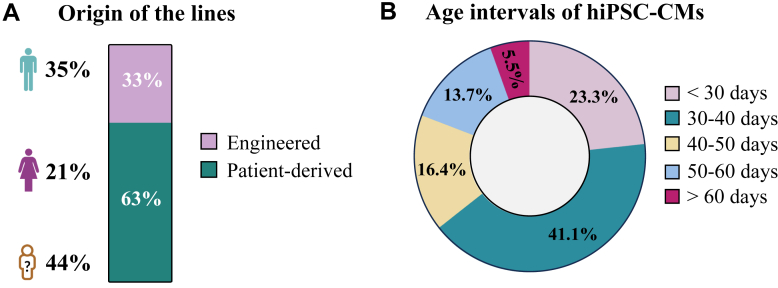
Origin of Cell Lines and Age of hiPSC-CMs (A) The percentage of hiPSC lines categorized by their origin, specifically by sex and method used generate them. (B) The percentage of studies that conducted experiments with hiPSC-CMs at different age ranges. Abbreviations as in Figure 7.

The majority of the hiPSC lines (93 of 132) are heterozygous, whereas 18 were homozygous, and 6 lines carried double (compound heterozygous) mutations (Figure 7D). While the majority of HCM mutations are known to be missense mutations, mutations leading to gain or loss of the stop codon or frameshift mutations could lead to haploinsuffiency.^245^ In particular, heterozygous *MYBPC3* truncating mutations underlie haploinsufficiency of wild-type cMyBP-C in human surgical specimens, and reduced expression of truncating mutant messenger RNA.^246^^,^^247^ Out of reported hiPSC-CMs carrying heterozygous *MYBPC3* gene variants, 4 lines (KO, R943X, R502W, and a line carrying trans-splicing mutation c.1358-1359insC) reported lower messenger RNA or protein levels, while cMyBP-C protein was absent in the homozygous R943X line.^248^ A total of 31.2% of the lines were generated from a male donor (n = 34), while only 15.9% were registered as female donor (n = 20), and for the remaining lines, the sex was not specified. A total of 63.1% of cell lines were patient derived (n = 64), in 33.3% mutations were introduced in control hiPSCs by gene editing (n = 31), and from 3.5% it is unknown (n = 3) (Figure 8A).

We extracted data from experiments including cell hypertrophy, sarcomere disarray, electrophysiology, calcium handling, and contractility of hiPSC-CMs, which could indicate variant-mediated arrhythmic behavior or impaired relaxation. To establish the presence of cellular hypertrophy, cell size was analyzed in 35 studies. Of 52 examined lines, 37 mutant lines demonstrated cellular hypertrophy compared with the control line. The majority of the tested lines were heterozygous, and of 6 homozygous mutant lines, 3 lines showed hypertrophy. KO of *TNNT2* in hiPSC-CMs did not result in hypertrophy.^249^ The presence of cellular hypertrophy in these mutant lines may depend on the age of the cell lines. One study observed increased cell size in a 60-days old mutant line, though no hypertrophy was observed at 39 days compared with the control line.^250^ Ojala et al^251^ demonstrated an increase in cell size from 1 to 3 weeks and up to 6 weeks for the *MYBPC3* mutant cell lines, while for the *TPM1* mutant cell lines, a significant increase was observed only from 1 to 3 weeks.

In 39 studies action potentials, calcium transients, and contractility were evaluated in a total of 65 hiPSC-CMs lines, of which 42 lines (13 *MYBPC3*, 12 *MYH7*, 4 *TNNT2*, 3 *ACTC1*, 1 *ACTN2*, 1 *KCNQ1*, 1 *MLP/MYH7*, 1 *MT-RNR2*, 1 *MYL2*, 1 *MYL3*, 1 *TNNI3*, 1 *TPM1*, and 1 *WTIP*) showed impaired relaxation. We found reports of impaired relaxation in hiPSC-CMs that were 11 days old up to cells that were 74 days old. In 11 studies, engineered heart tissues (3-dimensional) were used to assess the contractility of 17 hiPSC-CMs lines, of which 11 out of 17 lines showed impaired relaxation. The type of mutation is important, as 4 papers showed that impaired relaxation depends on the specific mutation, given that the method and age were consistent across these studies.^54^^,^^252^, ^253^, ^254^ Zygosity can make a difference, Strimaityte et al^255^ showed that the homozygous *MYBPC3* KO line exhibited impaired relaxation, while this is absented in the heterozygous line. However, the levels of MYBPC3 were not confirmed. One study showed no prolonged relaxation at 35 and 40 days but observed impaired relaxation at 45 days.^256^ Some studies elegantly combined measurements of action potentials, calcium handling, and contractility and were able to distinguish mutation-specific sarcomere changes from secondary, likely adaptive changes in calcium handling.^257^

Sarcomere disarray was studied in 22 papers with 29 cell lines, in which 17 cell lines reported sarcomeric disarray. Liang et al^54^ reported no disarray in the *KCNQ1* (G259S) cell line but did report on disarray in the *MYH7* (R663H) line. Tanaka et al^23^ showed that prolonged culture of 60 and 90 days over 30-day culture can induce disarray. All the *MYH7* mutant lines (E848G, R403Q, R442G, R663H, R453C, and P710R) that were checked for disarray showed sarcomeric disarray in the hiPSCs. All *ACTN2* lines carrying different gene variants (T247M, Q860X, and indel) demonstrated sarcomere disarray, whereas most of the reports on *MYBPC3* show no disarray, except for c.1358-1359insC (4 out 5 papers, with 5 out 6 cell lines). Even hetero- and homozygous KO *MYBPC3* lines in 2 different studies showed no disarray.

Proarrhythmic behavior was assessed in 36 lines in 24 studies, where 30 lines (9 *MYBPC3*, 8 *MYH7*, 4 *TNNT2*, 3 *ACTC1*, 3 *MYL3*, 1 *ALPK3*, 1 *KCNQ1*, 1 *MT-RNR2*, 1 *MYH6/MYH7*, 1 *MYL2*, 1 *PRKAG2*, 1 *TPM1*) showed proarrhythmic behavior. Of those lines, 8 lines have shown proarrhythmic behavior after stimulation with angiotensin ΙΙ, isoprenaline, or by increasing extracellular calcium concentration. Interestingly, Hsieh et al^258^ showed increased beating irregularity at 30 and 45 days, which was absent at days 15 and 60.

Overall, among all studies, only 2 patient-derived hiPSC-CMs lines carrying a heterozygous R442G *MYH7* mutation, and the R58Q variant (zygosity not mentioned) in *MYL2* showed all 4 HCM hallmarks mentioned previously (ie, cellular hypertrophy, sarcomere disarray, impaired relaxation, and proarrhythmic behavior) (Table 2). *MYL2* R58Q is registered as a pathogenic mutation, while the pathogenicity of *MYH7* R442G is unknown (ClinVar). Ma et al^259^ could not find any HCM hallmarks in gene-edited and patient-derived hiPSC-CMs carrying the heterozygous and homozygous A57D *MYL3* variant, on which conflicting pathogenicity results have been reported in ClinVar. The above reported measurements evaluating the cellular disease phenotype in hiPSC-CMs have been performed on different platforms or at different cell ages (Figure 8B). Therefore, it is important to consider the potential effect of these conditions on the reported cellular phenotypes. Out of 61 hiPSC-CM publications, 14 studies used engineered heart tissues of which 6 have conducted measurements in both 3 and 2 dimensions. We checked the age of the cells at which HCM hallmarks were measured and found that measurements were performed between 7 and 85 days, with 41.1% of studies being performed between day 30 and 40 (Figure 8B). To further understand the effect of hiPSC-CM age on the results, we refer to 8 studies in which measurements were performed at different time points.^13^^,^^23^^,^^250^^,^^251^^,^^255^, ^256^, ^257^, ^258^

**Table 2:** iPSC-CM Models With Main HCM Disease Characteristics

First Author, Year	Gene of Interest	Mutation	Zygosity	Impaired Relaxation	CM Hypertrophy	Proarrhythimia	Sarcomere Disarray
Ramachandra et al, 2021^337^	*MYH7*	R243C	Heterozygous	x	x	x	
Cohn et al, 2019^338^	*MYH7*	R403Q	Heterozygous	x	x		x
Han et al, 2014^339^	*MYH7*	R442G	Heterozygous	x	x	x	x
Mosqueira et al, 2018^7^	*MYH7*	R453C	Heterozygous		x	x	x
Mosqueira et al, 2018^7^	*MYH7*	R453C	Homozygous		x	x	x
Bhagwan et al, 2020^340^	*MYH7*	R453C	Heterozygous	x	x	x	
Liang et al, 2013^54^	*MYH7*	R663H			x	x	x
Riaz et al, 2022^253^	*MYH7/MLP*	R723C/W4R	Compound heterozygous	x	x		x
Pioner et al, 2016^341^	*MYH7*	R848G		x	x		x
Ramachandra et al, 2021^337^	*MYBPC3*	D389V	Heterozygous	x	x	x	
Ojala et al, 2016^251^	*MYBPC3*	Q1061X		x	x	x	
Kondo et al, 2022^342^	*TNNT2*	Δ160E	Heterozygous	x	x	x	
Wang et al, 2018^343^	*TNNT2*	I79N	Heterozygous	x		x	x
Bhagwan et al, 2020^340^	*ACTC1*	E99K	Heterozygous	x	x	x	
Prondzynski et al, 2019^344^	*ACTN2*	T247M	Heterozygous	x	x		x
Zhou et al, 2019^345^	*MYL2*	R58Q		x	x	x	x
Li et al, 2018^346^	*MT-RNR2*	m.2336T>C		x	x	x	

Selection of 14 publications that include 17 iPSC-CMs based on the reported presence of at least 3 hallmarks. An “x” indicates the presence of the hallmark.

iPSC-CM = induced pluripotent stem cell–derived cardiomyocyte; other abbreviations as in Table 1.

### Study quality

#### Animal models (n = 508)

**Figure 9:**
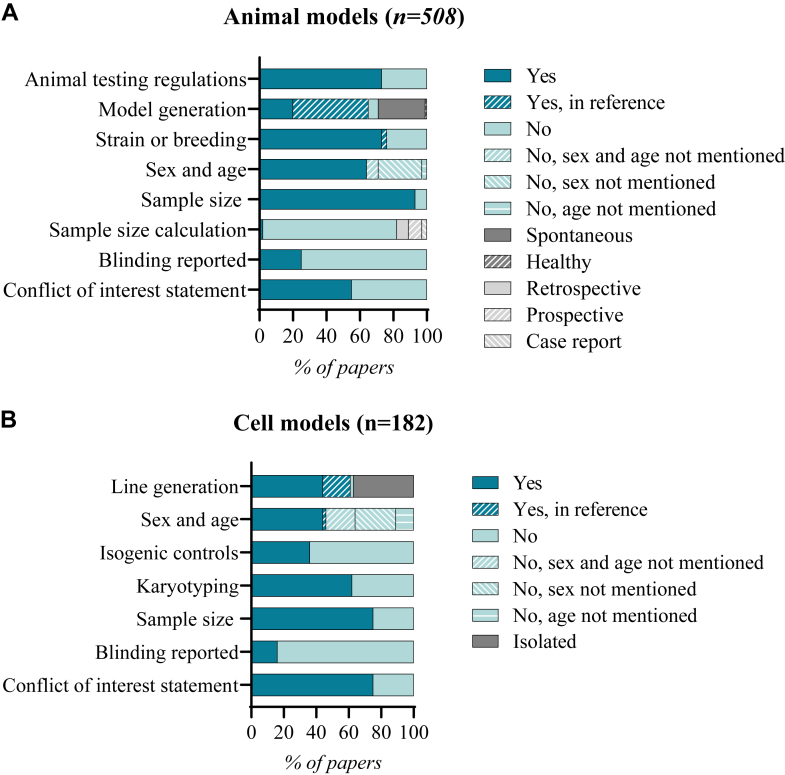
Quality Assessment (A, B) Assessment of study quality of all studies reporting on findings in animals and in cell models, based on the CAMARADES checklist and the RIVER recommendations. Questions could be answered with “yes;” “yes in reference,” when descriptive data could be found in referenced publications; and “no.” For the animal models, models could be generated via transgene approach, spontaneous, or healthy. For some studies, the sample size could not be calculated, as they were retrospective studies, prospective studies, or case reports.

We assessed the quality of our studies based on the CAMARADES checklist for study quality.^260^ For the studies that report on animal models, 73% mention they comply with the regulations and legislation associated with animal testing (Figure 9A, Supplemental Figure 2A). The generation of the animal model was mentioned in 20% of the studies, while 45% refer to other papers for the methods of model generation. A spontaneous HCM model was reported in 28% of the included studies. The strain, breeding, or husbandry of the animals was reported in the majority (73%) of studies, as well as the sex and age (64%). In the case of missing data, the most frequently omitted factor was the sex of the animals (26%), followed by both the sex and age of the animals (7%). The least frequent missing factor was the age of the animals (3%). Sample size was reported in 93% of the studies, but only 2% reported the justification or calculation of the sample size. The blinding of experiments and analyses were mentioned in 25% of the studies. A conflict of interest statement was found in 55% of studies.

#### Cell models (n = 182)

Here, we included the studies that performed experiments on cells (eg, studies on stem cell–derived models, transgenic human cell lines, or isolated cells from animals). To our best knowledge, there has not been any publication regarding the assessment of study quality of studies that report on cell models. Therefore, we designed a custom assessment tool, based on the CAMARADES checklist, the RIVER recommendations developed by the working group of the UK’s National Organization for the 3Rs,^261^ and recommendations from a systematic review on stem cell–derived models of HCM.^261^ Almost half of the studies (44%) reported how the cell lines were generated, and 17% contained a reference to other studies in which the lines were generated (Figure 9B, Supplemental Figure 2B). The sex and age of the donors of cell lines were reported in 44% of the studies. The sex was not mentioned in 25% of the studies, sex and age were not mentioned in 18% of studies, and age was not mentioned in 11% of papers. The stem cell–specific parameters on isogenic line generation and karyotyping were performed in 36% and 62% of the stem cell studies, respectively. The sample size was mentioned in 75% of the papers. The blinding of experiments and analyses was only reported in 16% of included studies. A conflict of interest statement was given in 75% of studies.

### Risk of bias

#### Animal models (n = 508)

**Figure 10:**
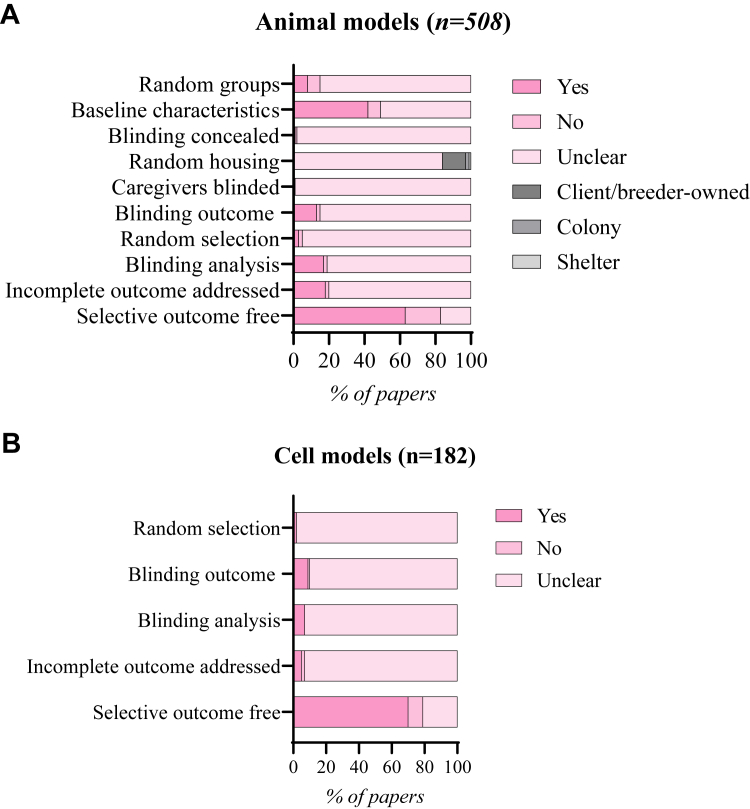
Risk-of-Bias Assessment (A, B) Assessment of the risk of bias in all studies reporting on findings in animals and in cell models, based on SYRCLE’s tool for risk-of-bias assessment. Questions could be answered with “yes,” “no,” or “unclear.” For some of the studies, the animals were not housed in-house, so additional options “client/breeder-owned,” “colony,” or “shelter” were available.

The risk on bias of studies reporting on animal models was based on SYRCLE’s tool for risk-of-bias assessment with signaling questions relating to potential sources of bias.^262^ For many studies, it was difficult to assess the risk of bias as much information was missing (Figure 10A, Supplemental Figure 3A). For studies that used multiple groups and tested the effects of treatment or diet, 8% of studies reported the random assignment of animals to groups. Out of these studies, the code or sequence procedure for the randomization was described in 4 papers. Random housing of animals was described in <1% of papers. In general, it was mostly unclear whether there were any statistical differences in age and/or sex between diseased and healthy animals (51%). Significant differences in age and/or sex were found in 7% of studies. For blinding, the most reported type was the analysis (17%), followed by blinding of the outcome assessor (13%). The least frequently reported blinding type was blinding of the caregivers (<1%). Animals that were randomly selected for outcome measurements were described in 3% of papers. For 80% of the papers, it could not be determined whether the outcome data resulted from the sample size of animals at the beginning of the study. We also searched for selective outcome reporting, such as exaggerating effects with “almost significant” values, conclusions drawn from data that were not shown or large reported effect sizes based on a small sample size. Most of the studies were free from selective outcome reporting (63%).

#### Cell models (n = 182)

The risk on bias of studies reporting on cell models was also based on SYRCLE’s tool for risk-of-bias assessment, as there is currently—to our best knowledge—no tool available for determining the potential sources of bias for cellular studies. For many studies, it was therefore difficult to assess the risk of bias (Figure 10B, Supplemental Figure 3B). We could assess potential sources of bias from the reporting of blinding. The most frequently reported type of blinding was the outcome assessor (9%), followed by blinding of the analysis (7%). Cells that were randomly selected for outcome measurements were described in 2% of the papers. Most of the studies were free from selective outcome reporting (70%).

## Discussion

To our knowledge, this is the first publication that provides a complete overview of all the HCM models studied thus far. This review, with all its compiled extracted data, could serve as a logbook in time to evaluate the use of these in vivo*/*in vitro models. Additionally, this overview could enable fellow researchers to find the model that is the most fit-for-purpose for answering their research questions, thereby preventing unnecessary repetitive studies and also promoting more conscious use of experimental animals, and optimal design of studies in hiPSC-derived heart models. In the following, we discuss several items which triggered our attention based on collided data. We hope that our review will initiate further discussions to maximize quality of studies using HCM models.

### HCM models used for (non)therapeutic strategies

Our primary focus was to provide an overview of the different models and how well they represent the HCM phenotype. In studies investigating therapeutic or nontherapeutic strategies, we did not include the treatment groups. However, the reported models have contributed to the development of new treatment strategies and their translation to clinical practice. As highlighted in Table 1, among the 35 studies that presented at least 4 hallmarks, only 2 tested therapies within mouse models.^81^^,^^264^ These publications focused on characterizing the mouse model, which provides a crucial foundation for subsequent research. Mouse models with the most frequently studied *Myh6* mutation R403Q have been used to evaluate new therapeutic strategies, including in vivo gene editing,^11^^,^^265^ Fropofol,^266^ a small-molecule derivative of propofol, and the myosin inhibitors mavacamten and aficamten.^11^^,^^267^ In Mybpc3 KI mice treatment with irbesartan,^268^ epoxomicin,^269^ nebivolol in skinned ventricular trabeculae,^270^ and diltiazem in isolated CMs have been investigated.^83^ While mouse models are commonly used to evaluate therapies, they are not the only animal model used for testing novel therapies. Feline models have also been used to study similar drugs, including antiplatelet medicine clopidogrel,^271^^,^^272^ cardiac myosin inhibitor CK-586,^273^^,^^274^ and rivaroxaban, both alone and in combination with enoxaparin and clopidogrel.^275^ Based on the beneficial effect of myosin inhibitors in patients, it can be concluded that HCM models that recapitulate relevant HCM features are of great value to translate basic science to development and clinical implementation of new drugs.

### Transition from animal models to stem cell–derived heart models

The translation of basic science results to clinical practice is a challenging task and warrants experimental models that reflect main human disease characteristics. As highlighted in the position paper from working groups of the European Society of Cardiology,^56^ choosing the most optimal experimental model is key and depends on the research questions. As such, the current overview highlights strengths and limitations of current experimental HCM models, which we will briefly discuss subsequently.

While it is often stated that there is no reduction in animal experimentation, our review provides data that show that within HCM research there is a clear shift from studies in animals to studies using stem cell–derived models (Figure 2C). Our review illustrates that advances have been made in animal models that evolved from protein KO to targeted gene-edited models, though only 1 mouse model ticked all boxes that define the human HCM phenotype (Table 1). Nonetheless, KO models provided essential information about the role of a specific (sarcomere) protein in cardiac development, structure and function of the heart, and in particular transgenic mouse models with thin filament mutations do show major human HCM disease characteristics. These models provided essential new knowledge on cellular pathomechanisms caused by sarcomere mutant proteins, and identified changes related to secondary disease remodeling of the heart.

While most patients are heterozygous, several heterozygous mouse models do not show a clear cardiac phenotype. In particular, heterozygous *Mybpc3* mutant mice do not show a phenotype unless severely stressed, while homozygous mutant animals show an extremely severe cardiac phenotype.^276^ LVH, a defining aspect of human HCM, is rarely present in heterozygous mutant *Mybpc3* mice. Recent studies highlight the need for a second disease hit (eg, aging, diet, exercise or TAC),^62^^,^^85^^,^^92^^,^^277^^,^^278^ which is also based on the human HCM population in which disease expression is heterogeneous, and may be explained by comorbidities and genetic modifiers. Mouse models that mimic heart failure with preserved ejection fraction have been designed by combining several comorbidities, and successfully recapitulate human cardiac disease features.^56^ A similar approach may prove effective in currently available heterozygous mouse models, and warrants further research. Several mouse models show specific cellular changes that are observed in human pathology, and therefore may be used for proof-of-concept studies to show effectiveness of therapeutic interventions. Recent examples are interventions targeting microtubule detyrosination, which is significantly increased in human HCM and may underlie diastolic dysfunction.^276^ Studies in 2 HCM mouse models, with a homozygous Dutch *Mybpc3* founder mutation and a transgenic knock-in of an Italian *Mybpc3* founder mutation, provided evidence that acute and chronic reduction of microtubule detyrosynation by tubulin tyrosine ligase improves CM and cardiac systolic and diastolic performance.^276^^,^^279^

The shift toward the use of human stem cell–derived cell models (Figure 2C) illustrates that several research questions can be answered with well-validated human heart models. Noteworthy, similar to mouse models, only a limited number of reported hiPSC-CM lines show all relevant HCM characteristics (Table 2), and also here, homozygous mutant lines more often show a clear cellular disease phenotype compared with heterozygous mutant lines. The limited number of hiPSC-CM lines that recapitulate all HCM features may be partly explained by the fact that not all parameters were studied, and emphasizes the need for in depth and complete analyses of cellular characteristics that define human cardiac disease.^262^ While we here focused on 4 aspects (cell hypertrophy, impaired relaxation, arrhythmic behavior, and sarcomere disarray), recent HCM studies highlight the relevance of energetic and metabolic cellular changes.^280^^,^^281^ Thus, studies on metabolism and mitochondrial function in hiPSC-derived heart models represent an essential aspect of future HCM stem cell research.^282^

### Research in domestic animals with naturally occurring HCM

An attractive alternative for gene-edited experimental animal models is the animal population with naturally occurring HCM, which range from domestic animals, such as cats and dogs, to more exotic species including kangaroo and even alpaca. The largest population studied thus far are cats (Figure 5). Most cats are domestic and have different living conditions, which makes genotype-phenotype studies challenging, though it does reflect the situation present in human. Therefore, studies in cats could help expand our knowledge about HCM disease development and genotype-phenotype heterogeneity. Reported studies in domestic animals illustrate the importance of genetic analyses and cardiac phenotyping, which thus far has not been done consistently (Figure 5A). Investment in cohorts of domestic animals from different breeds that show relevant HCM characteristics (Figure 6), including biobanking, is needed and offers the opportunity to expand HCM research which will ultimately benefit both animals and human with HCM.

### Study design taking into account zygosity and sex- and age-related differences

In past years it has become increasingly clear that scientists should carefully rethink experimental design of their studies to increase quality and, importantly, reproducibility of data. Inclusion of sufficient experimental and biologic replicates is essential,^56^ and also full reporting of study design is crucial to appreciate the true value of a publication. Our systematic review, including analyses of study quality (Figure 9) and risk of bias (Figure 10), illustrates that quality of research can be increased relatively easily by reporting data on sex, age, zygosity, and genotype, and bias may be prevented by increasing the number of blinding steps in experimental study designs.

The inconsistent reporting of key parameters such as zygosity, sex, and age in studies complicates future research, making it difficult to pinpoint when specific hallmarks of HCM appear, and to determine the best models or optimal timing for a treatment. In the section “Effects in Zygosity, Sex, and Aging on HCM Characteristics in Mouse Models”, we highlighted the reported differences across these variables. The takeaway is clear: altering these parameters can significantly affect the data, emphasizing 2 critical points. First, it is essential to report all relevant data, and second, a thorough characterization of your model is necessary to understand how it behaves. Two studies that stand out in this regard are Carrier et al,^62^ who examined heterozygous and homozygous *Mybpc3* KO mice across different ages and sexes, and Vikstrom et al,^88^ who studied 2 different mice lines with a different level of mutant protein (0.6%-2.5% and 10%-12%) at various ages and in both sexes.

Part of the heterogeneity in HCM clinical phenotype can be attributed to differences in sex and age.^283^^,^^284^ Our review illustrates that data on sex of the cells of origin, and age of hiPSC-derived cells/tissues at the time of functional/structural data collection, have not been reported consistently, and emphasizes the need for reporting such data to better understand mutation-specific changes, and (mal)adaptive changes that occur secondary to mutation-induced defects. In addition, data collection at different time points (age) will be a helpful strategy to avoid missing relevant HCM disease characteristics.

### LV outflow tract obstruction and microvascular perturbations

In our data extraction, we did not include left ventricular outflow tract obstruction (LVOTO) and microvascular abnormalities, though these are relevant disease characteristics of human HCM. The main reason for this was that these parameters were only scarcely present and /or reported.

LVOTO, defined by LVOT peak pressure gradient of ≥30 mm Hg, is present in approximately 25% to 33% of the patients with HCM at rest, increasing to 70% when provoked by exercise or physiologic maneuvers such as the Valsalva maneuver.^285^, ^286^, ^287^, ^288^ The presence of LVOTO increases the risk of progression to NYHA functional class III/IV symptoms or death.^289^ LVOTO is observed in 33% to 63% of cats with HCM. However, unlike humans, the presence of LVOTO in cats has not been associated with increased morbidity.^290^

Because cats closely mimic the human HCM phenotype, they serve as a valuable model for developing novel treatments aimed to treat features of HCM, including LVOTO. Rivas et al^273^ showed that a single oral dose of cardiac myosin inhibitor CK-586 improved or resolved LVOTO. In contrast, mouse models with common mutations in *Myh6*, *Mybpc3*, *Tnnt2*, and other HCM-related genes typically present a mild phenotype, and none of these models develop LV obstruction. Sørensen et al^291^ developed a mouse model with overexpression of ErbB2tg, which demonstrated LVOTO on Doppler imaging and elevated LV gradients compared with wild-type mice.

Microvascular and capillary abnormalities along with small vessel disease are found in many patients.^292^ A reduced number of capillaries, and/or a thickening of the medial and intimal layer of the intramural coronary, often resulting in a narrowed lumen, significantly contribute to disease progression.^293^, ^294^, ^295^, ^296^ Studies suggest that reduced capillary density, combined with increased oxygen demand due to hypertrophied CMs, leads to myocardial ischemia, which can trigger fibrosis and CM necrosis.^297^, ^298^, ^299^, ^300^, ^301^ This ischemia arises from inadequate coronary perfusion, driven by small vessel disease and impaired coronary flow dynamics.^302^, ^303^, ^304^ Additionally, remodeling of the extracellular matrix, influenced by hypoxia and inflammatory processes, exacerbates myocardial damage. These microvascular dysfunctions promote fibrosis.^305^

Microvascular problems are also observed in HCM animal models, although not many studies report on the vasculature in these models. The statements regarding microvascular change in the papers included in this review are as follows: In feline, canine, and pigs, abnormal intramural coronary arteries characterized by thickening of the intima and media of arterial wall, particularly in the ventricular septum, were identified.^5^^,^^43^^,^^187^^,^^188^^,^^190^, ^191^, ^192^^,^^223^^,^^224^^,^^306^, ^307^, ^308^, ^309^, ^310^ Moreover, capillary density was found to be reduced within the myocardium of cats.^185^^,^^311^^,^^312^ In hamsters at 80 days of age, an increased number of capillary vessels was observed, whereas a reduced number of capillaries was found at 150 days of age compared with control.^142^ Vikstrom et al^88^ demonstrated that abnormal coronary vessels phenotypes, exhibiting vessel-to-vessel heterogeneity, were found in the transgenic mouse carrying a R403Q + deletion of amino acids 468 to 527 mutation in *Myh6*. Using cardiac magnetic resonance, differences in myocardial blood flow between C57BL/6J and DBA/2J mice were studied.^313^ The DBA/2J mice strain contains 6 different sequence variants in *Myh6* and *Mybpc3* compared with C57BL/6J strain.^314^ Revealing reduced myocardial blood flow in DBA/2J mice, particularly in the septal and inferior axes. Additionally, staining confirmed reduced capillary density and increased interstitial and perivascular fibrosis in the DBA/2J mice.^313^ Healthy and mild medial hypertrophy of intramural coronary arteries with luminal narrowing was noted in the case report of an alpaca.^48^ Three studied reported the absence of thickening of the media of intramural coronary arteries in R403Q *Myh6* mutant mice, D175N and E180G *Tpm1* mutant mice, and *M mulatta*.^154^^,^^299^^,^^315^

## Conclusions

Despite the fact that it has been only a decade since the first hiPSC-CMs have been used to model HCM, and it is a relatively new model compared with conventional animal models, many studies investigated HCM pathomechanism in hiPSC-CMs that show 1 or more human HCM disease characteristics. As the technology to culture and measure different characteristics of these cells is constantly evolving, standardization of the measurement conditions and emphasis on reproducibility remains a key point for future advancement and studies using hiPSC-CM. In addition, animal models of HCM have proven clear benefits, and are still essential in testing interventions that cannot be done in cell systems yet. Our review emphasizes the need for consistent and full reporting of data, both in animal and human cell studies, and robust and unbiased design of experiments and data analyses, which will increase reproducibility in HCM research. Moreover, this review can serve as a guide for researchers in selecting the optimal model to address their research questions by referencing the data overview file, which details the specific hallmarks reported for each model.

## Funding Support and Author Disclosures

Ms van den Dolder and Dr van der Velden were supported by the Dutch Cardiovascular Alliance grant Double Dose 2021 funded by the Netherlands Heart Foundation. Ms Dinani, Mr Warnaar, and Dr van der Velden were supported by NWO-ZonMW (91818602 VICI grant). Ms Passadouro, Mr Nassar, and Dr van der Velden were supported by the Proper Therapy project funded by the Dutch Research Council, domain Applied and Engineering Sciences. Dr van der Velden was supported by the Association of Collaborating Health Foundations, ZonMW within the Human models 2.0 call, and fondation Leducq research grant 20CVD01. The authors have reported that they have no relationships relevant to the contents of this paper to disclose.
