# Supplementary material for: Experimental Models of Hypertrophic Cardiomyopathy: A Systematic Review
Source: JACC Basic Transl Sci. 2025 Jan 15;10(4):511–46. doi: 10.1016/j.jacbts.2024.10.017 (PMC12134605; doi:10.1016/j.jacbts.2024.10.017)
Supplement: Supplemental Material [file mmc1.pdf]

Supplemental appendix

Supplemental Table 1. Control groups mouse 2
Supplemental Table 2. Strain of mouse models 3
Supplemental Table 2 (continued). Strain of mouse models 4
Supplemental Table 3. Signaling questions for the quality and risk of bias assessment 5
Supplemental Figure legends 6
Supplemental Figure 1 7
Supplemental Figure 2 8
Supplemental Figure 3 9
Supplemental Methods 10
Supplemental Methods: Data extraction guidelines 11
Supplemental References Mouse models 34
Supplemental References Cat models 70
Supplemental References iPSC models 82

Supplemental Table 1. Control groups mouse

Control	Number of papers	Percentage
Wild-type	47	14.7
Wild-type age-matched	31	9.7
Wild-type sex-matched	13	4.1
Wild-type age- and sex-matched	21	6.6
Littermates	36	11.3
Littermates age-matched	19	5.9
Littermates sex-matched	10	3.1
Littermates age- and sex-matched	23	7.2
Non-transgenic	31	9.7
Non-transgenic age-matched	14	4.4
Non-transgenic sex-matched	8	2.5
Non-transgenic age- and sex-matched	18	5.6
Non-transgenic with secondary disease hit	1	0.3
Transgenic WT protein	4	1.3
Transgenic WT human protein	12	3.8
Transgenic WT human protein sex and age-matched	1	0.3
Diseased male	4	1.3
Diseased male age matched	1	0.3
Diseased female age matched	1	0.3
Diseased older	1	0.3
Diseased older sexs matched	1	0.3
Diseased without secondary disease hit	1	0.3
Diseased without secondary disease hit age- and sex-matched	1	0.3
Healthy	4	1.3
Healthy with secondary disease hit	3	0.9
Blank	14	4.4

Supplemental Table 2. Strain of mouse models

	Strain	Number of papers	Percentage
	Blank/ not reported	93	28.8
**Black Swiss**			
Main strain	Black Swiss	30	9.3
**C57/Bl6**			
Main strain	C57/Bl6*	39	12.1
	C57BL/6J	34	10.5
	C57BL/6N	6	1.9
Mixed strains	B6SJL	14	4.3
	B6/SJL	1	0.3
	B6SJL/F1	3	0.9
	B6SJLF1/J	1	0.3
	B6129 S/J	1	0.3
	BL6SJF1/J	6	1.9
	C57Bl6/C3H	1	0.3
Crossbreeding desribed	C57/Bl6 x 129S6/SvEvTac x 129x1/SvJ	1	0.3
	C57Bl/6 x 129S6	1	0.3
	C57Bl/6 x 129sv	2	0.6
	C57BL/6J x 129/Ola	1	0.3
	C57/Bl6 x CBA/Ca	1	0.3
	C57BL6 x CBA/Ca	2	0.6
	C57BL/6J x FVB	1	0.3
	C57B6 x FVB/N	3	0.9
	C57BL/6 x SJL	2	0.6
**FVB**			
Main strain	FVB/N	23	7.1
	FVB	2	0.6
Crossbreeding desribed	FVB/CF1 x C57BL/6J	1	0.3
	FVB/N x C57/B6	1	0.3

Supplemental Table 2 (continued). Strain of mouse models

**129 mice**			
Main strain	129SvEv	13	4.0
	SV/129	8	2.5
	129SvE	1	0.3
	129/SvJ	2	0.6
	E129X1	1	0.3
Mixed strains	129S/C57BL6	1	0.3
	129/BS	4	1.2
	129/Black Swiss	2	0.6
	129SvEv/S4	1	0.3
Crossbreeding desribed	129/SvJ x 129SvEv	1	0.3
	129S1 x Sv	1	0.3
	129SvEv x B6SJL	1	0.3
	E129X1 x FVB/N	1	0.3
	E129X1 x SvJ	3	0.9
**Other**			
	DBA/2J	2	0.6
	Mixed	2	0.6
	C3H x He	1	0.3
	CBA/B16	1	0.3
	CBA/Ca	1	0.3
	CD1	1	0.3
	ICR	1	0.3
	ICR/B6C3F1	1	0.3

Number of papers that (do not) describe the strain of the mouse model. Mouse strains can be divided in sup-families (color blocks) and we further categorized them in main strain, mix strains and crossbred strains. Crossbred strains are often described as F1 hybrid mice.

Supplemental Table 3. Signaling questions for the quality and risk of bias assessment

Animal models

Quality assessment	Risk of bias assessment
Do the authors declare compliance with animal testing regulations and legislation?	Were groups of animals randomly assigned to diseased/healthy groups?
Do the authors describe how the model was generated (e.g. transgene approach)?	Were the groups similar in age AND/OR sex at the start of the measurements/adjusted for confounders in the analysis?
Are the strain, breeding OR husbandry conditions reported?	Was the code/sequence for the random assignment of animals adequately concealed?
Are the sex AND the age of the animals mentioned in the paper?	Were the animals housed randomly during the experiment?
Is the sample size mentioned in the paper?	Were any of the caregivers blinded?
Did the authors describe how the sample size was calculated?	Was the outcome assessor blinded?
Was any blinding reported? (e.g. outcome assessment, allocation of animals, data analysis)	Were animals selected at random for outcome assessment?
Is there a statement of a potential conflict of interest?	Was the analysis blinded?
	Are incomplete outcome data addressed?
	Is the study free of selective outcome reporting?

Cell models

Quality assessment	Risk of bias assessment
Do the authors describe how lines were generated?	Were cells selected at random for outcome assessment?
Are the sex and the age of the donor(s) mentioned?	Was the outcome assessor blinded?
Are isogenic controls used?	Are incomplete outcome data addressed?
Are cell lines karyotyped?	Is the study free of selective outcome reporting?
Is the sample size mentioned in the paper?	
Is there a statement of a potential conflict of interest?	

Supplemental Figure 1. Sex and age of the mouse model.

The percentage of studies using mouse models, categorized by different sexes and age ranges.

Supplemental Figure 2. Quality assessment

Assessment of study quality separated for each animal model and type of cell model in order of the number of papers included in this study. Questions could be answered with 'yes', 'yes in reference', when descriptive data could be found in referenced publications, and 'no'. For the animal models, models could be generated via transgene approach, spontaneous or healthy. For some studies, the sample size could not be calculated as they were retrospective, prospective studies or case reports. Cell models could be generated or contain isolated cells from transgenic animals.

Supplemental Figure 3. Risk of bias assessment

Assessment of the risk on bias separated for each animal model and type of cell model in order of the number of papers included in this study**.** Questions could be answered with 'yes', 'no' or 'unclear'. For some of the studies, the animals were not housed in-house, so additional options 'client/breeder-owned', 'colony' or 'shelter' were available.

Supplemental Figure 1.

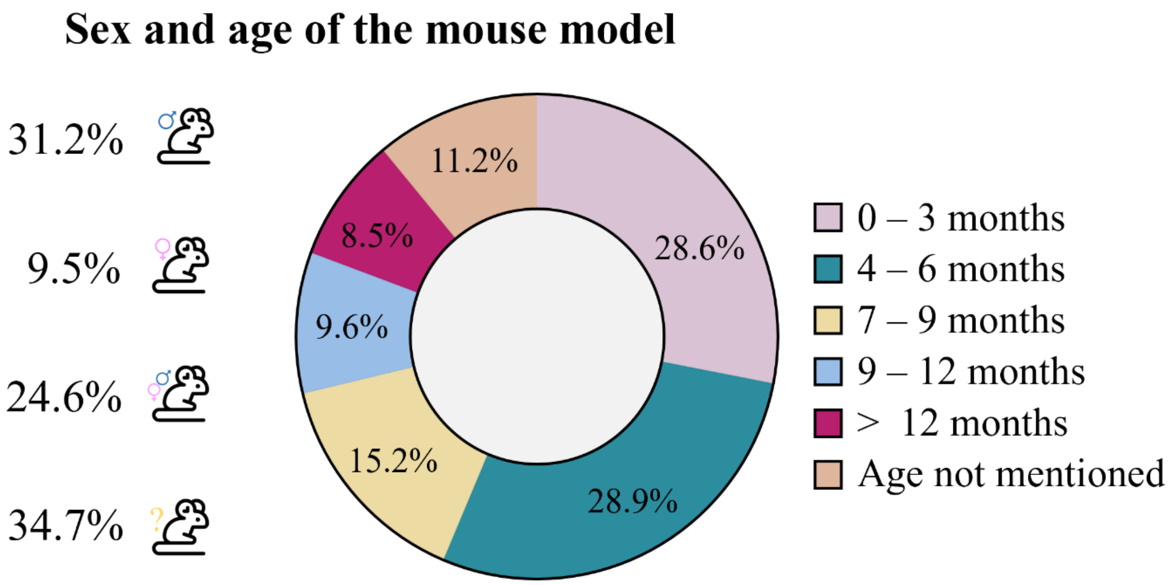

Supplemental Figure 2.

A

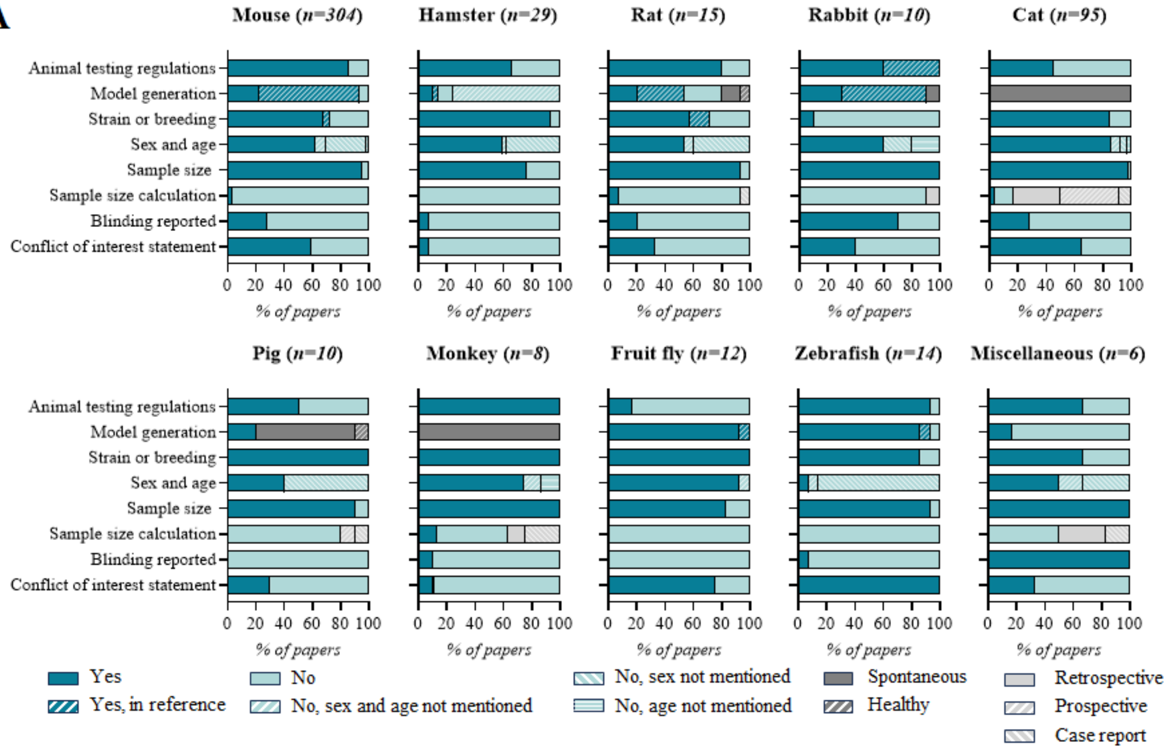

B

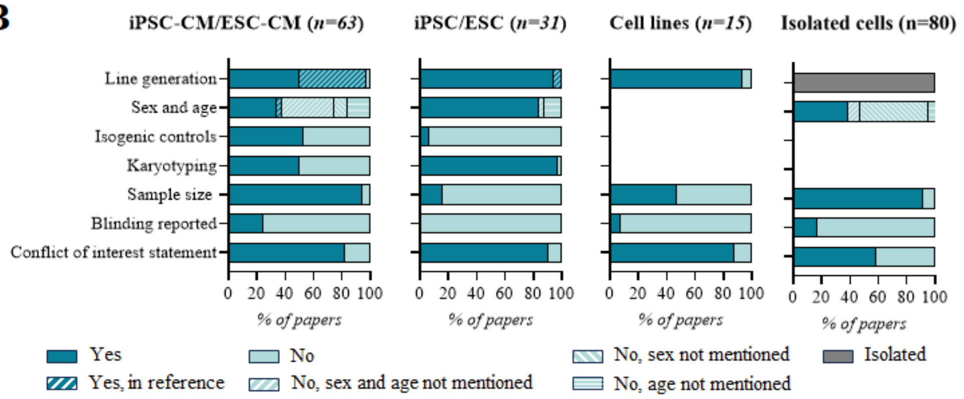

Supplemental Figure 3.

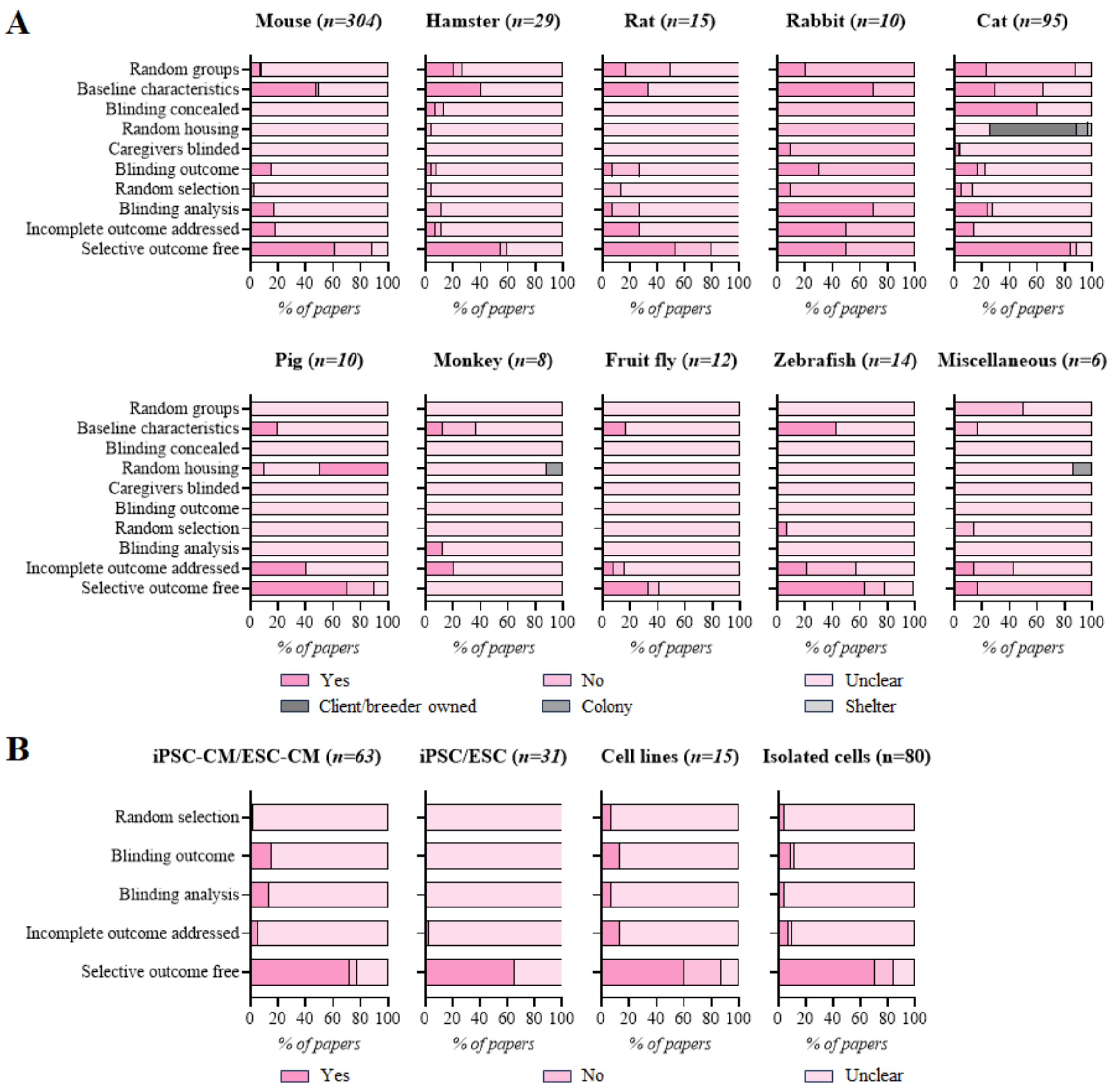

Supplemental Methods

Below are examples of search strategies used for two of the used databases:

Final Search: Embase (4,390)

'hypertrophic cardiomyopathy'/de OR 'familial hypertrophic cardiomyopathy'/exp OR 'hypertrophic obstructive cardiomyopathy'/exp OR (‘HCM’ OR ‘hypertrophic cardiomyopath*’ OR ‘hypertrophic obstructive cardiomyopath*’):ti,ab,kw

AND

'animal model'/exp OR 'isolated heart'/exp OR 'biological model'/de OR 'disease model'/de OR 'disease simulation'/exp OR 'model'/de OR 'animal testing alternative'/exp OR 'multicellular spheroid'/exp OR 'zebra fish'/exp OR 'experimental mouse'/de OR 'mouse mutant'/exp OR 'mouse strain'/exp OR

'induced pluripotent stem cell'/exp OR (‘model*’ OR ‘in vivo*’ OR ‘in vitro*’ OR ‘invivo*’ OR ‘invitro*’ OR ‘Stem Cell*’ OR ‘IPSC-CM*’ OR ‘HPSC-CM*’ OR ‘HIPSC*’ OR ‘cardiomyocyte*’ OR ‘cardio myocyte*’ OR ‘Isolated Heart Preparation*’ OR ‘Cellular Spheroid*’ OR ‘Zebrafish*’ OR ‘Zebra fish*’ OR ‘C57BL’ OR ‘Biological Assay*’):ti,ab,kw

Final search Web of Science:

TS=(“HCM” OR “hypertrophic cardiomyopath*” OR “hypertrophic obstructive cardiomyopath*”)

AND

TS=(“model*” OR “in vivo*” OR “in vitro*” OR “invivo*” OR “invitro*” OR “Stem Cell*” OR “IPSC-CM*” OR “HPSC-CM*” OR “HIPSC*” OR “cardiomyocyte*” OR “cardio myocyte*” OR “Isolated Heart Preparation*” OR “Cellular Spheroid*” OR “Zebrafish*” OR “Zebra fish*” OR “C57BL” OR “Biological Assay*”)

Animal model – general

- When to fill this out?
  - Anything from an animal that is more than 1 cell
    - In vivo measurements
    - Ex vivo measurements (e.g. perfused hearts)
    - Whole hearts
    - Ventricular wedges
    - Papillary muscles
    - Intact muscles, muscle strips, (skinned) myofibers or –fibrils
  - Exception: CM hypertrophy can only be measured in 1 cell, but this single-cell measurement is included in the animal model part of the Excel sheet

Year of publication (column D)

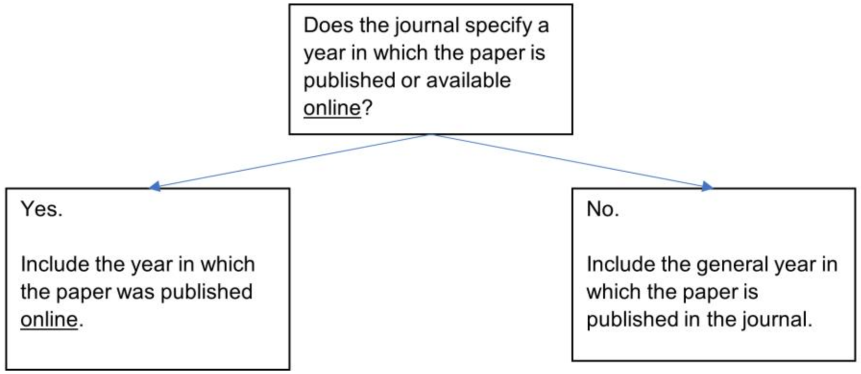

Gene (column E) and Mutation name (column F)

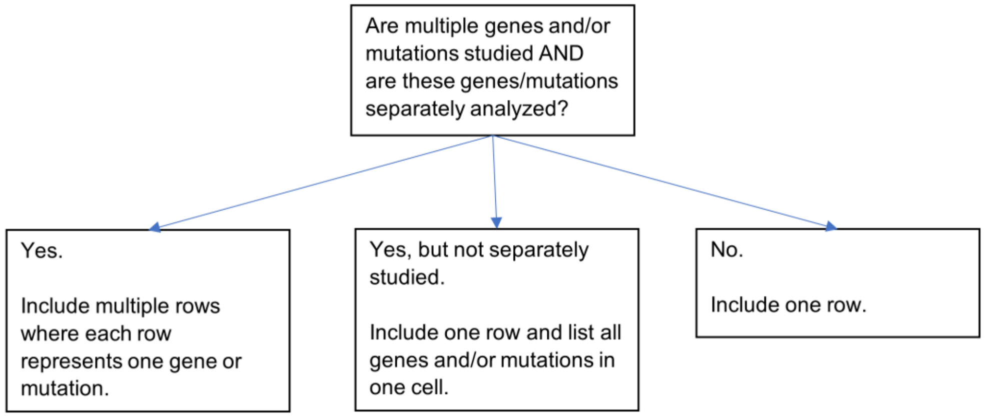

Pathogenicity (column G)

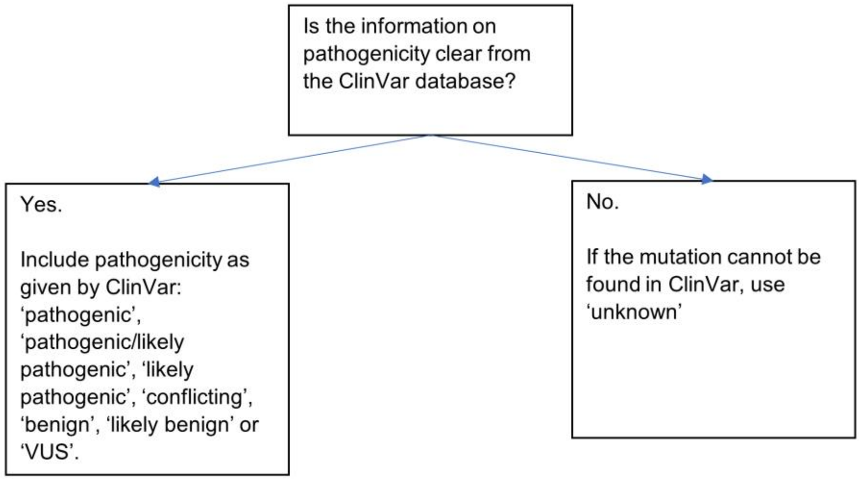

Zygosity (Column J)

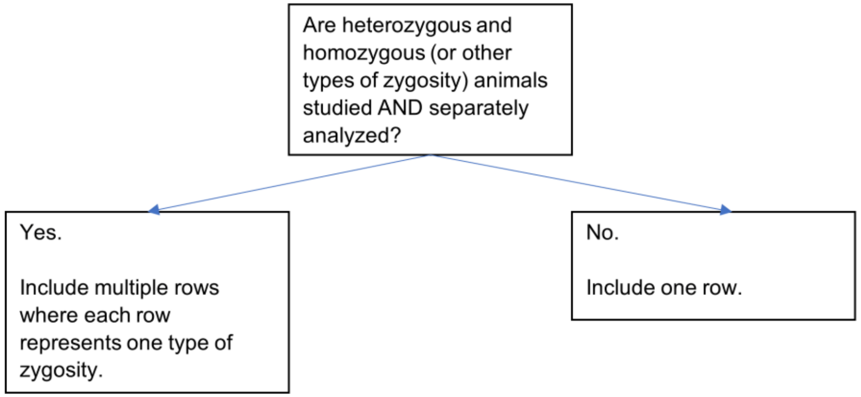

% mutant protein expression (Column K)
New name: mRNA/protein levels

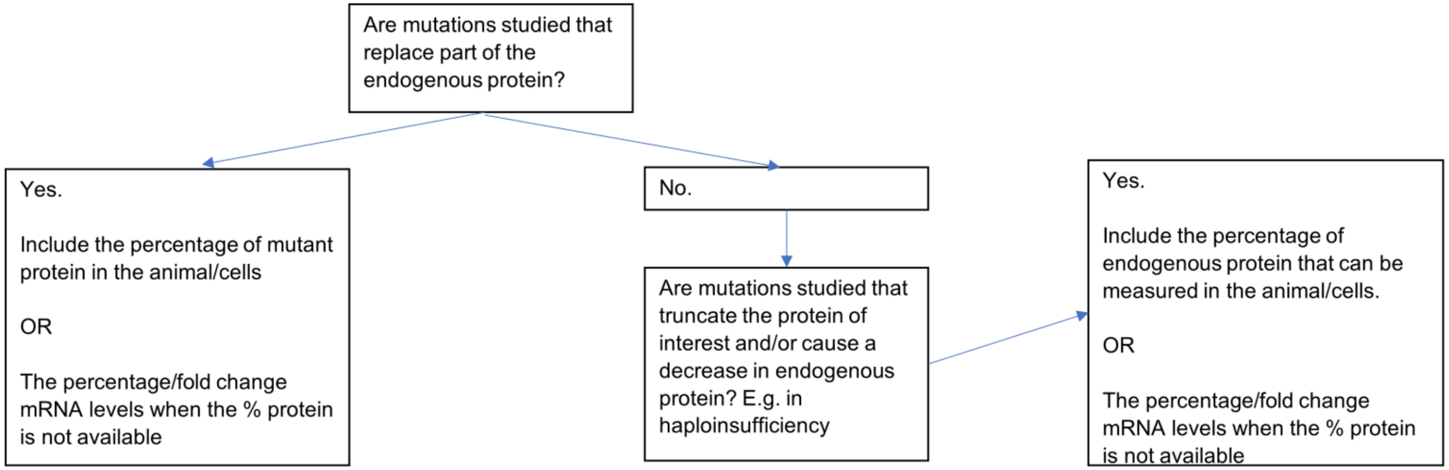

When both mutated protein and endogenous protein levels are found, note the % of mutant protein, not the % of endogenous protein

→ Added additional column (L) with the following answering options:
- Protein, mutant
- Protein, endogenous
- mRNA, mutant
- mRNA, endogenous

Confirmation of mRNA/protein levels (column M) - NEW

- Answering options:
  - Western blot
    - Quantified
  - Northern blot
    - Quantified
  - SDS Page
    - Quantified
  - Immunoblot
    - Quantified
  - Slot blot
    - Quantified

Confirmation of mutation (column N)

- Only concerns the confirmation of the mutation on the gene level. Answering options:
  - PCR
  - Genotyping
  - Sequencing
  - STR analysis
  - Southern blot

Breeds (column P)

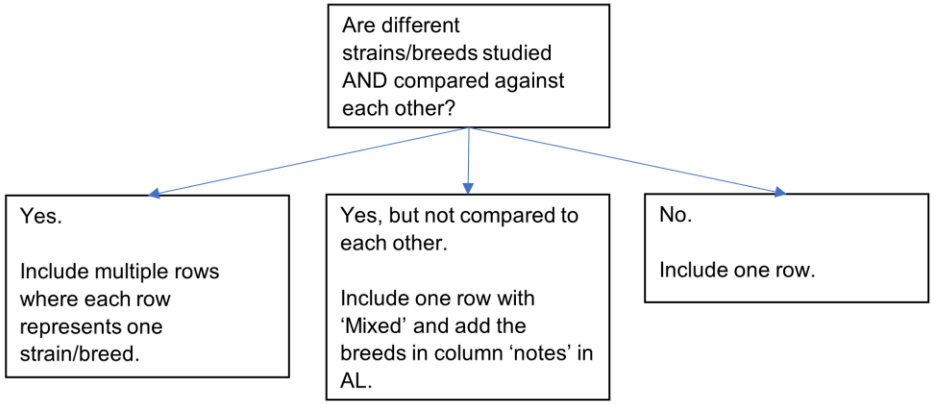

Sex (column Q)

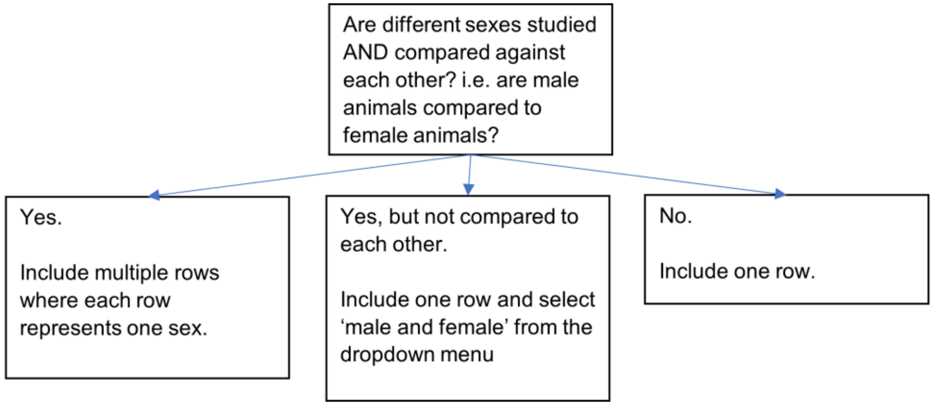
Are different sexes studied AND compared against each other? i.e. are male animals compared to female animals?
Yes.
Include multiple rows where each row represents one sex.
Yes, but not compared to each other.
Include one row and select 'male and female' from the dropdown menu
No.
Include one row.

Age (column R)

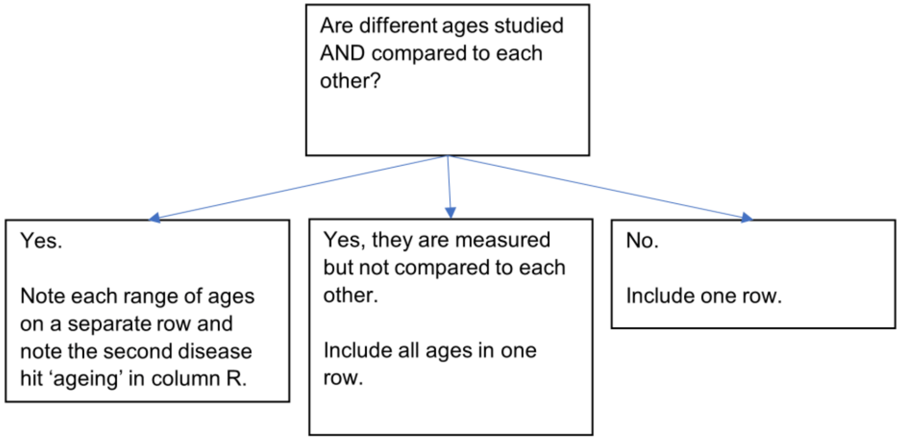

Changes: multiple ages that are not compared? Combine in one row and do not create multiple rows

Survival (column T)

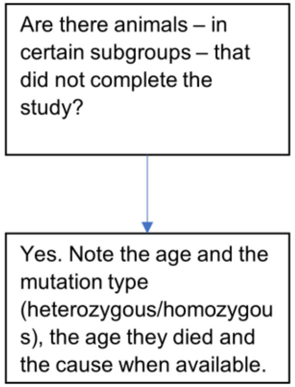

Changes: also list cause of death when available

Control (column W)

Which type of animals serve as a control?

- **Non-transgenic** → When the authors explicitly mention that the animals were non-transgenic
- **Transgenic WT (human) protein** → When they create transgenic animals with the wild-type human protein
- **Littermates** → When the authors mention that control animals were wild-type littermates
- **Wild-type** → When the authors mention that the animals were wild-type or do not specify whether the animals were non-transgenic, expressed the human wt protein or that they were littermates
- **Vector, morpholino, scrambled control** → When they genetically engineer the animals and the control consists of the animal with a vector/transfection reagent/control genetic mix
- **Diseased animals, e.g. double transgenic to single transgenic animals** → Do not include a row for a comparison to a diseased animal

When age-matched? → When:
- It is explicitly mentioned
- There are no statistically significant differences between the ages

When sex-matched? → When:
- It is explicitly mentioned
- There are no statistically significant differences between the sexes
- When they only measure one sex

Diastolic dysfunction (column X & Ak)
New name: ‘impaired relaxation’

Impaired relaxation

For hearts and (skinned) fibers/fibrils/muscle strips

Indication: described ‘diastolic dysfunction’ or ‘impaired relaxation’

Statistically significant:
- E/A (increase)
- E/e’ (increase)
- Deceleration time (increase)
- IVRT (increase)
- LVEDP (increase)
- QT (prolonged)
- Left atrium dilation
- Tau (increase)
- Relaxation time (increase)
- Relaxation rate (Krel) (decrease)
- Relaxation velocity (decrease)
- Calcium sensitivity (increase)

For cardiomyocytes

Indication: described ‘impaired relaxation’

Statistically significant:
- Impaired cardiomyocyte relaxation
    - Relaxation time (increase)
    - APD (prolonged)
- Calcium overload in diastole
- Calcium transient duration (prolonged)
- Relaxation rate (Krel) (decrease)
- Calcium sensitivity (increase)

Hypertrophy (column Y) & CM hypertrophy (column Z & AL)

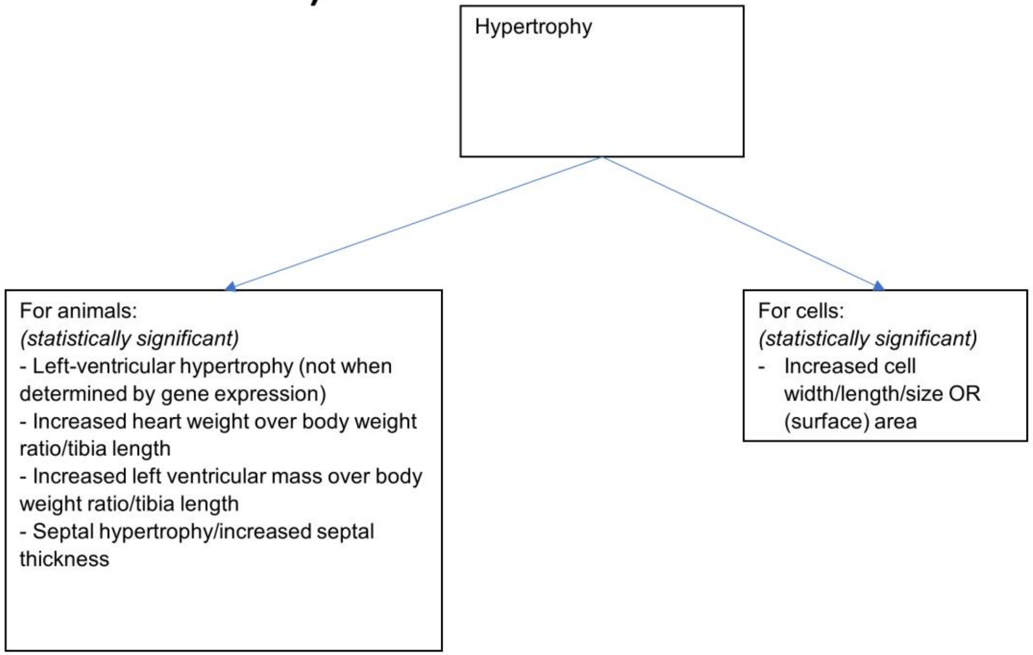

Fibrosis (column AA)

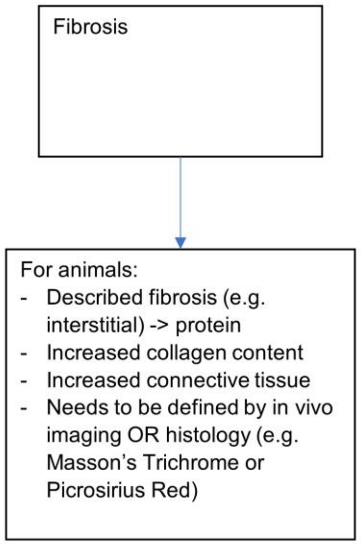

Answering options:
- Yes (described, shown and quantified)
- Yes; described, not shown (when fibrosis is described, but there is no histology shown)
- Yes; shown, not quantified (when fibrosis is described, shown by e.g. histology, but there is no quantification)
- No

Ventricular arrhythmia (column AB & AM)
New name for cell-based models: ‘proarrhythmia’

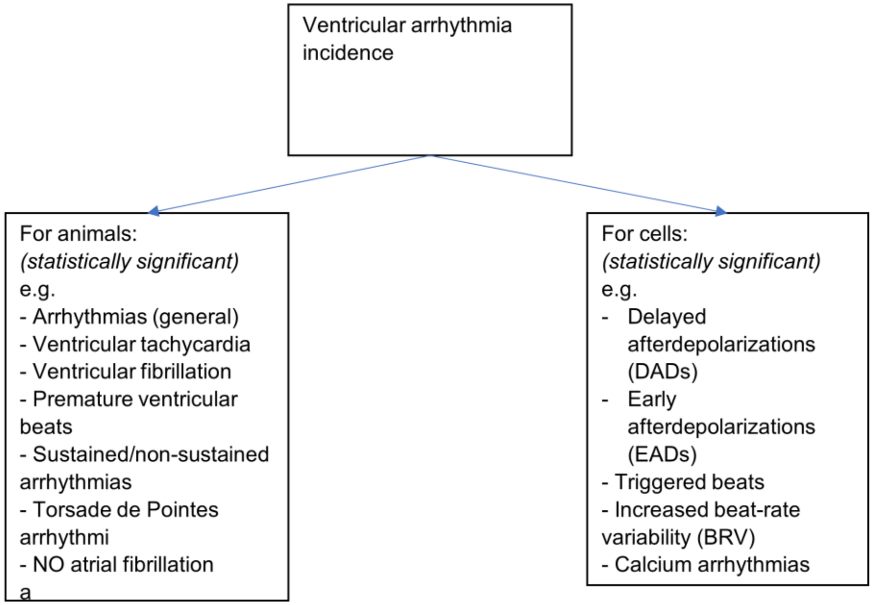

Answering options:
- Yes
- Yes, after stimulation
- No
- No, after stimulation

Change: added ‘NO atrial fibrillation’ to animal part

Myocardial/sarcomeric disarray (column AC & AN)

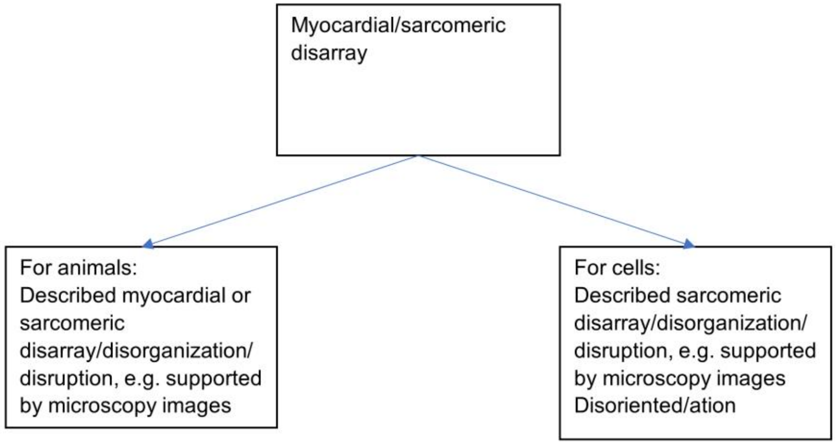

Changes: no statistical significance necessary, added word ‘disruption’ to both and ‘sarcomeric’ to animals

Answering options:

- Yes (described, shown and quantified)
- Yes; described, not shown (when disarray is described, but there are no images (e.g. EM) shown)
- Yes; shown, not quantified (when disarray is described, shown by images (e.g. EM), but there is no quantification)
- No

Cell-based model – general

- When to fill this out?
  - Any single cells used for cell-based experiments: calcium overload, relaxation, cellular (pro)arrhythmia etc.
    - Cardiomyocytes isolated from an animal
    - Digested cardiomyocytes
    - Stem-cell derived models (iPSC/ESC-based)
    - Cell lines (e.g. HEK293)
  - Do not fill out this category when cells are isolated but NOT measured
  - Category is not to be used as a default option for CM hypertrophy, since this category is already included in the animal model part of the Excel

Species (column AF)

- Not to fill out when cells are isolated from animals

Sex (column AG)

- For cells isolated from animals: only fill out when the sex is specified for the isolated cells, especially when this is different from the sex of the animals (e.g. male and female animals are included, but cells were only isolated from male animals)

Origin (column AI)

- Not to fill out when cells are isolated from animals

Age (column AH) – NEW

- Specify the age of the cells for functional measurements (e.g. cardiomyocyte hypertrophy, impaired relaxation, proarrhythmia and disarray)
  - List different ages for different experiments together within one cell
- If functional measurements are performed at different ages and there are no differences between the ages → combine ages in one row
- If functional measurements are performed at different ages and there are differences between the ages→ create multiple rows for the ages that are different

Supplemental References Mouse models

1. Abraham TP, Jones M, Kazmierczak K et al. Diastolic dysfunction in familial hypertrophic cardiomyopathy transgenic model mice. Cardiovasc Res 2009;82:84-92.
2. Agnew T, Goldsworthy M, Aguilar C et al. A Wars2 Mutant Mouse Model Displays OXPHOS Deficiencies and Activation of Tissue-Specific Stress Response Pathways. Cell Rep 2018;25:3315-3328.e6.
3. Ahola S, Rivera Mejias P, Hermans S et al. OMA1-mediated integrated stress response protects against ferroptosis in mitochondrial cardiomyopathy. Cell Metab 2022;34:1875-1891 e7.
4. Al Moamen NJ, Prasad V, Bodi I et al. Loss of the AE3 anion exchanger in a hypertrophic cardiomyopathy model causes rapid decompensation and heart failure. J Mol Cell Cardiol 2011;50:137-46.
5. Alvarado FJ, Bos JM, Yuchi Z et al. Cardiac hypertrophy and arrhythmia in mice induced by a mutation in ryanodine receptor 2. JCI Insight 2019;5.
6. Alves ML, Dias FAL, Gaffin RD et al. Desensitization of myofilaments to Ca2+ as a therapeutic target for hypertrophic cardiomyopathy with mutations in thin filament proteins. Circ Cardiovasc Genet 2014;7:132-143.
7. Awinda PO, Watanabe M, Bishaw Y et al. Mavacamten decreases maximal force and Ca(2+) sensitivity in the N47K-myosin regulatory light chain mouse model of hypertrophic cardiomyopathy. Am J Physiol Heart Circ Physiol 2021;320:H881-H890.
8. Bagnall RD, Tsoutsman T, Shephard RE, Ritchie W, Semsarian C. Global microRNA profiling of the mouse ventricles during development of severe hypertrophic cardiomyopathy and heart failure. PLoS One 2012;7:e44744.

9. Barefield D, Kumar M, de Tombe PP, Sadayappan S. Contractile dysfunction in a mouse model expressing a heterozygous MYBPC3 mutation associated with hypertrophic cardiomyopathy. Am J Physiol Heart Circ Physiol 2014;306:H807-15.

10. Barefield D, Kumar M, Gorham J et al. Haploinsufficiency of MYBPC3 exacerbates the development of hypertrophic cardiomyopathy in heterozygous mice. J Mol Cell Cardiol 2015;79:234-43.

11. Bartoli F, Evans EL, Blythe NM et al. Global PIEZO1 Gain-of-Function Mutation Causes Cardiac Hypertrophy and Fibrosis in Mice. Cells 2022;11.

12. Baudenbacher F, Schober T, Pinto JR et al. Myofilament Ca2+ sensitization causes susceptibility to cardiac arrhythmia in mice. J Clin Invest 2008;118:3893-903.

13. Becker L, Kling E, Schiller E et al. MTO1-deficient mouse model mirrors the human phenotype showing complex I defect and cardiomyopathy. PLoS One 2014;9:e114918.

14. Berul CI, Christe ME, Aronovitz MJ, Seidman CE, Seidman JG, Mendelsohn ME. Electrophysiological abnormalities and arrhythmias in alpha MHC mutant familial hypertrophic cardiomyopathy mice. J Clin Invest 1997;99:570-6.

15. Berul CI, McConnell BK, Wakimoto H et al. Ventricular arrhythmia vulnerability in cardiomyopathic mice with homozygous mutant Myosin-binding protein C gene. Circulation 2001;104:2734-9.

16. Bevilacqua LM, Maguire CT, Seidman JG, Seidman CE, Berul CI. QT dispersion in alpha-myosin heavy-chain familial hypertrophic cardiomyopathy mice. Pediatr Res 1999;45:643-7.

17. Birch CL, Behunin SM, Lopez-Pier MA et al. Sex dimorphisms of crossbridge cycling kinetics in transgenic hypertrophic cardiomyopathy mice. Am J Physiol Heart Circ Physiol 2016;311:H125-36.

18. Blanchard E, Seidman C, Seidman JG, LeWinter M, Maughan D. Altered crossbridge kinetics in the alphaMHC403/+ mouse model of familial hypertrophic cardiomyopathy. Circ Res 1999;84:475-83.

19. Blankenburg R, Hackert K, Wurster S et al. β-Myosin heavy chain variant Val606Met causes very mild hypertrophic cardiomyopathy in mice, but exacerbates HCM phenotypes in mice carrying other HCM mutations. Circ Res 2014;115:227-37.

20. Borejdo J, Szczesna-Cordary D, Muthu P, Calander N. Familial hypertrophic cardiomyopathy can be characterized by a specific pattern of orientation fluctuations of actin molecules. Biochemistry 2010;49:5269-5277.

21. Braumann S, Thottakara T, Stücker S et al. S100A4 as a Target of the E3-Ligase Asb2β and Its Effect on Engineered Heart Tissue. Front Physiol 2018;9:1292.

22. Burkart EM, Arteaga GM, Sumandea MP, Prabhakar R, Wieczorek DF, Solaro RJ. Altered signaling surrounding the C-lobe of cardiac troponin C in myofilaments containing an α-tropomyosin mutation linked to familial hypertrophic cardiomyopathy. Journal of Molecular and Cellular Cardiology 2003;35:1285-1293.

23. Cannon L, Yu ZY, Marciniec T et al. Irreversible triggers for hypertrophic cardiomyopathy are established in the early postnatal period. J Am Coll Cardiol 2015;65:560-9.

24. Captur G, Ho CY, Schlossarek S et al. The embryological basis of subclinical hypertrophic cardiomyopathy. Sci Rep 2016;6:27714.

25. Carrier L, Knöll R, Vignier N et al. Asymmetric septal hypertrophy in heterozygous cMyBP-C null mice. Cardiovasc Res 2004;63:293-304.

26. Chai AC, Cui M, Chemello F et al. Base editing correction of hypertrophic cardiomyopathy in human cardiomyocytes and humanized mice. Nat Med 2023;29:401-411.

27. Chandra M, Rundell VL, Tardiff JC, Leinwand LA, De Tombe PP, Solaro RJ. Ca(2+) activation of myofilaments from transgenic mouse hearts expressing R92Q mutant cardiac troponin T. Am J Physiol Heart Circ Physiol 2001;280:H705-13.
28. Chandra M, Tschirgi ML, Tardiff JC. Increase in tension-dependent ATP consumption induced by cardiac troponin T mutation. American Journal of Physiology - Heart and Circulatory Physiology 2005;289:H2112-H2119.
29. Chen H, Untiveros GM, McKee LA et al. Micro-RNA-195 and -451 regulate the LKB1/AMPK signaling axis by targeting MO25. PLoS One 2012;7:e41574.
30. Chen H, Hwang H, McKee LAK et al. Temporal and morphological impact of pressure overload in transgenic FHC mice. Frontiers in Physiology 2013;4.
31. Chen Y, Zhang Z, Hu F et al. 17β-estradiol prevents cardiac diastolic dysfunction by stimulating mitochondrial function: a preclinical study in a mouse model of a human hypertrophic cardiomyopathy mutation. J Steroid Biochem Mol Biol 2015;147:92-102.
32. Cheng Y, Wan X, McElfresh TA et al. Impaired contractile function due to decreased cardiac myosin binding protein C content in the sarcomere. Am J Physiol Heart Circ Physiol 2013;305:H52-65.
33. Chouchani ET, Methner C, Buonincontri G et al. Complex I deficiency due to selective loss of Ndufs4 in the mouse heart results in severe hypertrophic cardiomyopathy. PLoS One 2014;9:e94157.
34. Chowdhury SAK, Warren CM, Simon JN et al. Modifications of Sarcoplasmic Reticulum Function Prevent Progression of Sarcomere-Linked Hypertrophic Cardiomyopathy Despite a Persistent Increase in Myofilament Calcium Response. Front Physiol 2020;11:107.
35. Christodoulou DC, Wakimoto H, Onoue K et al. 5'RNA-Seq identifies Fhl1 as a genetic modifier in cardiomyopathy. J Clin Invest 2014;124:1364-70.

36. Chuan P, Sivaramakrishnan S, Ashley EA, Spudich JA. Cell-intrinsic functional effects of the α-cardiac myosin Arg-403-Gln mutation in familial hypertrophic cardiomyopathy. Biophys J 2012;102:2782-90.

37. Cibi DM, Bi-Lin KW, Shekeran SG et al. Prdm16 Deficiency Leads to Age-Dependent Cardiac Hypertrophy, Adverse Remodeling, Mitochondrial Dysfunction, and Heart Failure. Cell Rep 2020;33:108288.

38. Coppini R, Mazzoni L, Ferrantini C et al. Ranolazine Prevents Phenotype Development in a Mouse Model of Hypertrophic Cardiomyopathy. Circ Heart Fail 2017;10.

39. Coutu P, Bennett CN, Favre EG, Day SM, Metzger JM. Parvalbumin corrects slowed relaxation in adult cardiac myocytes expressing hypertrophic cardiomyopathy-linked α-tropomyosin mutations. Circulation Research 2004;94:1235-1241.

40. Crocini C, Ferrantini C, Scardigli M et al. Novel insights on the relationship between T-tubular defects and contractile dysfunction in a mouse model of hypertrophic cardiomyopathy. J Mol Cell Cardiol 2016;91:42-51.

41. Danielson LS, Park DS, Rotllan N et al. Cardiovascular dysregulation of miR-17-92 causes a lethal hypertrophic cardiomyopathy and arrhythmogenesis. Faseb j 2013;27:1460-7.

42. de Lange WJ, Hegge LF, Grimes AC et al. Neonatal mouse-derived engineered cardiac tissue: a novel model system for studying genetic heart disease. Circ Res 2011;109:8-19.

43. Debold EP, Schmitt JP, Patlak JB et al. Hypertrophic and dilated cardiomyopathy mutations differentially affect the molecular force generation of mouse alpha-cardiac myosin in the laser trap assay. Am J Physiol Heart Circ Physiol 2007;293:H284-91.

44. Desjardins CL, Chen Y, Coulton AT, Hoit BD, Yu X, Stelzer JE. Cardiac myosin binding protein C insufficiency leads to early onset of mechanical dysfunction. Circ Cardiovasc Imaging 2012;5:127-36.

45. Dhandapany PS, Kang S, Kashyap DP et al. Adiponectin receptor 1 variants contribute to hypertrophic cardiomyopathy that can be reversed by rapamycin. Science advances 2021;7.

46. Dieseldorff Jones KM, Koh Y, Weller RS et al. Pathogenic troponin T mutants with opposing effects on myofilament Ca(2+) sensitivity attenuate cardiomyopathy phenotypes in mice. Arch Biochem Biophys 2019;661:125-131.

47. Dieseldorff Jones KM, Vied C, Valera IC, Chase PB, Parvatiyar MS, Pinto JR. Sexual dimorphism in cardiac transcriptome associated with a troponin C murine model of hypertrophic cardiomyopathy. Physiol Rep 2020;8:e14396.

48. Dossat AM, Sanchez-Gonzalez MA, Koutnik AP et al. Pathogenesis of depression- and anxiety-like behavior in an animal model of hypertrophic cardiomyopathy. Faseb j 2017;31:2492-2506.

49. Dumka D, Talent J, Akopova I, Guzman G, Szczesna-Cordary D, Borejdo J. E22K mutation of RLC that causes familial hypertrophic cardiomyopathy in heterozygous mouse myocardium: effect on cross-bridge kinetics. Am J Physiol Heart Circ Physiol 2006;291:H2098-106.

50. Dusener S, Flenner F, Maack C et al. Ouabain worsens diastolic sarcomere length in myocytes from a cardiomyopathy mouse model. Eur J Pharmacol 2021;904:174170.

51. Dutsch A, Wijnker PJM, Schlossarek S et al. Phosphomimetic cardiac myosin-binding protein C partially rescues a cardiomyopathy phenotype in murine engineered heart tissue. Sci Rep 2019;9:18152.

52. Dweck D, Sanchez-Gonzalez MA, Chang AN et al. Long term ablation of protein kinase A (PKA)-mediated cardiac troponin I phosphorylation leads to excitation-contraction uncoupling and diastolic dysfunction in a knock-in mouse model of hypertrophic cardiomyopathy. J Biol Chem 2014;289:23097-111.

53. Ehsan M, Kelly M, Hooper C et al. Mutant Muscle LIM Protein C58G causes cardiomyopathy through protein depletion. J Mol Cell Cardiol 2018;121:287-296.

54. Eijssen LMT, van den Bosch BJC, Vignier N et al. Altered myocardial gene expression reveals possible maladaptive processes in heterozygous and homozygous cardiac myosin-binding protein C knockout mice. Genomics 2008;91:52-60.

55. Ertz-Berger BR, He H, Dowell C et al. Changes in the chemical and dynamic properties of cardiac troponin T cause discrete cardiomyopathies in transgenic mice. Proc Natl Acad Sci U S A 2005;102:18219-24.

56. Evans CC, Pena JR, Phillips RM et al. Altered hemodynamics in transgenic mice harboring mutant tropomyosin linked to hypertrophic cardiomyopathy. American Journal of Physiology - Heart and Circulatory Physiology 2000;279:H2414-H2423.

57. Farrell ET, Grimes AC, de Lange WJ, Armstrong AE, Ralphe JC. Increased Postnatal Cardiac Hyperplasia Precedes Cardiomyocyte Hypertrophy in a Model of Hypertrophic Cardiomyopathy. Front Physiol 2017;8:414.

58. Farrell E, Armstrong AE, Grimes AC, Naya FJ, de Lange WJ, Ralphe JC. Transcriptome Analysis of Cardiac Hypertrophic Growth in MYBPC3-Null Mice Suggests Early Responders in Hypertrophic Remodeling. Front Physiol 2018;9:1442.

59. Fatkin D, Christe ME, Aristizabal O et al. Neonatal cardiomyopathy in mice homozygous for the Arg403Gln mutation in the alpha cardiac myosin heavy chain gene. J Clin Invest 1999;103:147-53.

60. Fatkin D, McConnell BK, Mudd JO et al. An abnormal Ca(2+) response in mutant sarcomere protein-mediated familial hypertrophic cardiomyopathy. J Clin Invest 2000;106:1351-9.

61. Ferrantini C, Coppini R, Pioner JM et al. Pathogenesis of Hypertrophic Cardiomyopathy is Mutation Rather Than Disease Specific: A Comparison of the Cardiac Troponin T E163R and R92Q Mouse Models. J Am Heart Assoc 2017;6.

62. Fielitz J, Kim MS, Shelton JM et al. Myosin accumulation and striated muscle myopathy result from the loss of muscle RING finger 1 and 3. J Clin Invest 2007;117:2486-95.

63. Flenner F, Friedrich FW, Ungeheuer N et al. Ranolazine antagonizes catecholamine-induced dysfunction in isolated cardiomyocytes, but lacks long-term therapeutic effects in vivo in a mouse model of hypertrophic cardiomyopathy. Cardiovasc Res 2016;109:90-102.

64. Flenner F, Geertz B, Reischmann-Düsener S et al. Diltiazem prevents stress-induced contractile deficits in cardiomyocytes, but does not reverse the cardiomyopathy phenotype in Mybpc3-knock-in mice. J Physiol 2017;595:3987-3999.

65. Flenner F, Jungen C, Kupker N et al. Translational investigation of electrophysiology in hypertrophic cardiomyopathy. J Mol Cell Cardiol 2021;157:77-89.

66. Ford SJ, Mamidi R, Jimenez J, Tardiff JC, Chandra M. Effects of R92 mutations in mouse cardiac troponin T are influenced by changes in myosin heavy chain isoform. Journal of Molecular and Cellular Cardiology 2012;53:542-551.

67. Fraysse B, Weinberger F, Bardswell SC et al. Increased myofilament Ca2+ sensitivity and diastolic dysfunction as early consequences of Mybpc3 mutation in heterozygous knock-in mice. J Mol Cell Cardiol 2012;52:1299-307.

68. Freeman K, Colon-Rivera C, Olsson MC et al. Progression from hypertrophic to dilated cardiomyopathy in mice that express a mutant myosin transgene. Am J Physiol Heart Circ Physiol 2001;280:H151-9.

69. Freeman K, Lerman I, Kranias EG et al. Alterations in cardiac adrenergic signaling and calcium cycling differentially affect the progression of cardiomyopathy. J Clin Invest 2001;107:967-74.

70. Friedrich FW, Reischmann S, Schwalm A et al. FHL2 expression and variants in hypertrophic cardiomyopathy. Basic Research in Cardiology 2014;109.

71. Friedrich FW, Sotoud H, Geertz B et al. I-1-deficiency negatively impacts survival in a cardiomyopathy mouse model. Int J Cardiol Heart Vasc 2015;8:87-94.

72. Friedrich FW, Flenner F, Nasib M, Eschenhagen T, Carrier L. Epigallocatechin-3-Gallate Accelerates Relaxation and Ca(2+) Transient Decay and Desensitizes Myofilaments in Healthy and Mybpc3-Targeted Knock-in Cardiomyopathic Mice. Front Physiol 2016;7:607.

73. Gaffin RD, Chowdhury SA, Alves MS et al. Effects of nicotine administration in a mouse model of familial hypertrophic cardiomyopathy, α-tropomyosin D175N. Am J Physiol Heart Circ Physiol 2011;301:H1646-55.

74. Gaffin RD, Peña JR, Alves MS et al. Long-term rescue of a familial hypertrophic cardiomyopathy caused by a mutation in the thin filament protein, tropomyosin, via modulation of a calcium cycling protein. J Mol Cell Cardiol 2011;51:812-20.

75. Gao WD, Pérez NG, Seidman CE, Seidman JG, Marbán E. Altered cardiac excitation-contraction coupling in mutant mice with familial hypertrophic cardiomyopathy. J Clin Invest 1999;103:661-6.

76. Garcia-Canadilla P, Cook AC, Mohun TJ et al. Myoarchitectural disarray of hypertrophic cardiomyopathy begins pre-birth. J Anat 2019;235:962-976.

77. Garcia-Pelagio KP, Chen L, Joca HC, Ward C, Lederer WJ, Bloch RJ. Absence of synemin in mice causes structural and functional abnormalities in heart. Journal of Molecular and Cellular Cardiology 2018;114:354-363.

78. Gedicke-Hornung C, Behrens-Gawlik V, Reischmann S et al. Rescue of cardiomyopathy through U7snRNA-mediated exon skipping in Mybpc3-targeted knock-in mice. EMBO Mol Med 2013;5:1128-45.

79. Gehmlich K, Dodd MS, Allwood JW et al. Changes in the cardiac metabolome caused by perhexiline treatment in a mouse model of hypertrophic cardiomyopathy. Mol Biosyst 2015;11:564-73.

80. Geisterfer-Lowrance AA, Christe M, Conner DA et al. A mouse model of familial hypertrophic cardiomyopathy. Science 1996;272:731-4.

81. Georgakopoulos D, Christe ME, Giewat M, Seidman CM, Seidman JG, Kass DA. The pathogenesis of familial hypertrophic cardiomyopathy: early and evolving effects from an alpha-cardiac myosin heavy chain missense mutation. Nat Med 1999;5:327-30.

82. Gilda JE, Lai X, Witzmann FA, Gomes AV. Delineation of Molecular Pathways Involved in Cardiomyopathies Caused by Troponin T Mutations. Mol Cell Proteomics 2016;15:1962-81.

83. Giles J, Patel JR, Miller A, Iverson E, Fitzsimons D, Moss RL. Recovery of left ventricular function following in vivo reexpression of cardiac myosin binding protein C. J Gen Physiol 2019;151:77-89.

84. Gollapudi SK, Chandra M. The effect of cardiomyopathy mutation (R97L) in mouse cardiac troponin T on the muscle length-mediated recruitment of crossbridges is modified divergently by α- and β-myosin heavy chain. Arch Biochem Biophys 2016;601:105-12.

85. Gomes AV, Kazmierczak K, Cheah JX et al. Proteomic analysis of physiological versus pathological cardiac remodeling in animal models expressing mutations in myosin essential light chains. Journal of Muscle Research and Cell Motility 2015;36:447-461.

86. Gonzalez-Martinez D, Johnston JR, Landim-Vieira M et al. Structural and functional impact of troponin C-mediated Ca(2+) sensitization on myofilament lattice spacing and cross-bridge mechanics in mouse cardiac muscle. J Mol Cell Cardiol 2018;123:26-37.

87. Granzier HL, Radke MH, Peng J et al. Truncation of titin's elastic PEVK region leads to cardiomyopathy with diastolic dysfunction. Circ Res 2009;105:557-64.

88. Green EM, Wakimoto H, Anderson RL et al. A small-molecule inhibitor of sarcomere contractility suppresses hypertrophic cardiomyopathy in mice. Science 2016;351:617-21.

89. Greenberg MJ, Watt JD, Jones M, Kazmierczak K, Szczesna-Cordary D, Moore JR. Regulatory light chain mutations associated with cardiomyopathy affect myosin mechanics and kinetics. J Mol Cell Cardiol 2009;46:108-15.

90. Guinto PJ, Haim TE, Dowell-Martino CC, Sibinga N, Tardiff JC. Temporal and mutation-specific alterations in Ca2+ homeostasis differentially determine the progression of cTnT-related cardiomyopathies in murine models. Am J Physiol Heart Circ Physiol 2009;297:H614-26.

91. Haim TE, Dowell C, Diamanti T, Scheuer J, Tardiff JC. Independent FHC-related cardiac troponin T mutations exhibit specific alterations in myocellular contractility and calcium kinetics. J Mol Cell Cardiol 2007;42:1098-110.

92. Haines CD, Harvey PA, Luczak ED et al. Estrogenic compounds are not always cardioprotective and can be lethal in males with genetic heart disease. Endocrinology 2012;153:4470-4479.

93. Halas M, Langa P, Warren CM, Goldspink PH, Wolska BM, Solaro RJ. Effects of Sarcomere Activators and Inhibitors Targeting Myosin Cross-Bridges on Ca(2+)-Activation of Mature and Immature Mouse Cardiac Myofilaments. Mol Pharmacol 2022;101:286-299.

94. Hardt SE, Geng YJ, Montagne O et al. Accelerated cardiomyopathy in mice with overexpression of cardiac G(s)alpha and a missense mutation in the alpha-myosin heavy chain. Circulation 2002;105:614-20.

95. Harris SP, Bartley CR, Hacker TA et al. Hypertrophic cardiomyopathy in cardiac myosin binding protein-C knockout mice. Circulation Research 2002;90:594-601.

96. He H, Hoyer K, Tao H et al. Myosin-driven rescue of contractile reserve and energetics in mouse hearts bearing familial hypertrophic cardiomyopathy-associated mutant troponin T is mutation-specific. J Physiol 2012;590:5371-88.

97. Hernandez OM, Szczesna-Cordary D, Knollmann BC et al. F110I and R278C troponin T mutations that cause familial hypertrophic cardiomyopathy affect muscle contraction in transgenic mice and reconstituted human cardiac fibers. J Biol Chem 2005;280:37183-94.

98. Herwig M, Kolijn D, Lódi M et al. Modulation of Titin-Based Stiffness in Hypertrophic Cardiomyopathy via Protein Kinase D. Front Physiol 2020;11:240.

99. Hu LR, Ackermann MA, Hecker PA et al. Deregulated Ca(2+) cycling underlies the development of arrhythmia and heart disease due to mutant obscurin. Sci Adv 2017;3:e1603081.

100. Hu LYR, Kontrogianni-Konstantopoulos A. Proteomic Analysis of Myocardia Containing the Obscurin R4344Q Mutation Linked to Hypertrophic Cardiomyopathy. Frontiers in Physiology 2020;11.

101. Huang W, Liang J, Kazmierczak K et al. Hypertrophic cardiomyopathy associated Lys104Glu mutation in the myosin regulatory light chain causes diastolic disturbance in mice. J Mol Cell Cardiol 2014;74:318-29.

102. Huang W, Kazmierczak K, Zhou Z, Aguiar-Pulido V, Narasimhan G, Szczesna-Cordary D. Gene expression patterns in transgenic mouse models of hypertrophic cardiomyopathy caused by mutations in myosin regulatory light chain. Arch Biochem Biophys 2016;601:121-32.

103. Huang Y, Lu H, Ren X et al. Fropofol prevents disease progression in mice with hypertrophic cardiomyopathy. Cardiovascular research 2020;116:1175-1185.

104. Huang F, Na N, Ijichi T et al. Exosomally derived Y RNA fragment alleviates hypertrophic cardiomyopathy in transgenic mice. Mol Ther Nucleic Acids 2021;24:951-960.

105. Hueneke R, Adenwala A, Mellor RL, Seidman JG, Seidman CE, Nerbonne JM. Early remodeling of repolarizing K(+) currents in the αMHC(403/+) mouse model of familial hypertrophic cardiomyopathy. J Mol Cell Cardiol 2017;103:93-101.

106. Huke S, Venkataraman R, Faggioni M et al. Focal energy deprivation underlies arrhythmia susceptibility in mice with calcium-sensitized myofilaments. Circ Res 2013;112:1334-44.

107. Hunter JJ, Tanaka N, Rockman HA, Ross J, Jr., Chien KR. Ventricular expression of a MLC-2v-ras fusion gene induces cardiac hypertrophy and selective diastolic dysfunction in transgenic mice. J Biol Chem 1995;270:23173-8.

108. Iorga B, Blaudeck N, Solzin J et al. Lys184 deletion in troponin I impairs relaxation kinetics and induces hypercontractility in murine cardiac myofibrils. Cardiovasc Res 2008;77:676-86.

109. Ivandic BT, Mastitsky SE, Schönsiegel F et al. Whole-genome analysis of gene expression associates the ubiquitin-proteasome system with the cardiomyopathy phenotype in disease-sensitized congenic mouse strains. Cardiovasc Res 2012;94:87-95.

110. Jagatheesan G, Rajan S, Petrashevskaya N et al. Rescue of tropomyosin-induced familial hypertrophic cardiomyopathy mice by transgenesis. Am J Physiol Heart Circ Physiol 2007;293:H949-58.

111. James J, Zhang Y, Osinska H et al. Transgenic modeling of a cardiac troponin I mutation linked to familial hypertrophic cardiomyopathy. Circ Res 2000;87:805-11.

112. Javadpour MM, Tardiff JC, Pinz I, Ingwall JS. Decreased energetics in murine hearts bearing the R92Q mutation in cardiac troponin T. J Clin Invest 2003;112:768-75.

113. Jiang J, Wakimoto H, Seidman JG, Seidman CE. Allele-specific silencing of mutant Myh6 transcripts in mice suppresses hypertrophic cardiomyopathy. Science 2013;342:111-114.

114. Jimenez J, Tardiff JC. Abnormal heart rate regulation in murine hearts with familial hypertrophic cardiomyopathy-related cardiac troponin T mutations. Am J Physiol Heart Circ Physiol 2011;300:H627-35.

115. Jones WK, Grupp IL, Doetschman T et al. Ablation of the murine alpha myosin heavy chain gene leads to dosage effects and functional deficits in the heart. Journal of Clinical Investigation 1996;98:1906-1917.

116. Kannan S, Muthusamy VR, Whitehead KJ et al. Nrf2 deficiency prevents reductive stress-induced hypertrophic cardiomyopathy. Cardiovasc Res 2013;100:63-73.

117. Kawai M, Johnston JR, Karam T, Wang L, Singh RK, Pinto JR. Myosin Rod Hypophosphorylation and CB Kinetics in Papillary Muscles from a TnC-A8V KI Mouse Model. Biophys J 2017;112:1726-1736.

118. Kazmierczak K, Paulino EC, Huang W et al. Discrete effects of A57G-myosin essential light chain mutation associated with familial hypertrophic cardiomyopathy. Am J Physiol Heart Circ Physiol 2013;305:H575-89.

119. Kazmierczak K, Yuan CC, Liang J, Huang W, Rojas AI, Szczesna-Cordary D. Remodeling of the heart in hypertrophy in animal models with myosin essential light chain mutations. Front Physiol 2014;5:353.

120. Kazmierczak K, Liang J, Yuan CC et al. Slow-twitch skeletal muscle defects accompany cardiac dysfunction in transgenic mice with a mutation in the myosin regulatory light chain. Faseb j 2019;33:3152-3166.

121. Kazmierczak K, Liang J, Gomez-Guevara M, Szczesna-Cordary D. Functional comparison of phosphomimetic S15D and T160D mutants of myosin regulatory light chain exchanged in cardiac muscle preparations of HCM and WT mice. Front Cardiovasc Med 2022;9:988066.

122. Kerrick WG, Kazmierczak K, Xu Y, Wang Y, Szczesna-Cordary D. Malignant familial hypertrophic cardiomyopathy D166V mutation in the ventricular myosin regulatory light chain causes profound effects in skinned and intact papillary muscle fibers from transgenic mice. Faseb j 2009;23:855-65.

123. Kim SJ, Iizuka K, Kelly RA et al. An alpha-cardiac myosin heavy chain gene mutation impairs contraction and relaxation function of cardiac myocytes. Am J Physiol 1999;276:H1780-7.

124. Kimura H, Eguchi S, Sasaki J et al. Vps34 regulates myofibril proteostasis to prevent hypertrophic cardiomyopathy. JCI Insight 2017;2:e89462.

125. Knöll R, Kostin S, Klede S et al. A common MLP (muscle LIM protein) variant is associated with cardiomyopathy. Circ Res 2010;106:695-704.

126. Knollmann BC, Blatt SA, Horton K et al. Inotropic stimulation induces cardiac dysfunction in transgenic mice expressing a troponin T (I79N) mutation linked to familial hypertrophic cardiomyopathy. J Biol Chem 2001;276:10039-48.

127. Knollmann BC, Kirchhof P, Sirenko SG et al. Familial hypertrophic cardiomyopathy-linked mutant troponin T causes stress-induced ventricular tachycardia and Ca2+-dependent action potential remodeling. Circulation Research 2003;92:428-436.

128. Konhilas JP, Watson PA, Maass A et al. Exercise can prevent and reverse the severity of hypertrophic cardiomyopathy. Circ Res 2006;98:540-8.

129. Konhilas JP, Boucek DM, Horn TR, Johnson GL, Leinwand LA. The role of MEKK1 in hypertrophic cardiomyopathy. Int Heart J 2010;51:277-84.

130. Konno T, Chen D, Wang L et al. Heterogeneous myocyte enhancer factor-2 (Mef2) activation in myocytes predicts focal scarring in hypertrophic cardiomyopathy. Proceedings of the National Academy of Sciences of the United States of America 2010;107:18097-18102.

131. Kruger M, Zittrich S, Redwood C et al. Effects of the mutation R145G in human cardiac troponin I on the kinetics of the contraction-relaxation cycle in isolated cardiac myofibrils. J Physiol 2005;564:347-57.

132. Ku MC, Kober F, Lai YC et al. Cardiovascular magnetic resonance detects microvascular dysfunction in a mouse model of hypertrophic cardiomyopathy. J Cardiovasc Magn Reson 2021;23:63.

133. Kuster DWD, Lynch TL, Barefield DY et al. Altered C10 domain in cardiac myosin binding protein-C results in hypertrophic cardiomyopathy. Cardiovasc Res 2019;115:1986-1997.

134. Lam L, Tsoutsman T, Arthur J, Semsarian C. Differential protein expression profiling of myocardial tissue in a mouse model of hypertrophic cardiomyopathy. J Mol Cell Cardiol 2010;48:1014-22.

135. Leatherbury L, Yu Q, Chatterjee B et al. A novel mouse model of X-linked cardiac hypertrophy. Am J Physiol Heart Circ Physiol 2008;294:H2701-11.

136. Lee HG, Chen Q, Wolfram JA et al. Cell cycle re-entry and mitochondrial defects in myc-mediated hypertrophic cardiomyopathy and heart failure. PLoS One 2009;4:e7172.

137. Lehman SJ, Tal-Grinspan L, Lynn ML et al. Chronic Calmodulin-Kinase II Activation Drives Disease Progression in Mutation-Specific Hypertrophic Cardiomyopathy. Circulation 2019;139:1517-1529.

138. Li J, Mamidi R, Doh CY et al. AAV9 gene transfer of cMyBPC N-terminal domains ameliorates cardiomyopathy in cMyBPC-deficient mice. JCI Insight 2020;5.

139. Liang J, Kazmierczak K, Rojas AI, Wang Y, Szczesna-Cordary D. The R21C mutation in cardiac troponin i imposes differences in contractile force generation between the left and right ventricles of knock-in mice. BioMed Research International 2015;2015.

140. Lim HW, De Windt LJ, Mante J et al. Reversal of cardiac hypertrophy in transgenic disease models by calcineurin inhibition. J Mol Cell Cardiol 2000;32:697-709.

141. Lim DS, Oberst L, McCluggage M et al. Decreased left ventricular ejection fraction in transgenic mice expressing mutant cardiac troponin T-Q(92), responsible for human hypertrophic cardiomyopathy. J Mol Cell Cardiol 2000;32:365-74.

142. Lim DS, Lutucuta S, Bachireddy P et al. Angiotensin II blockade reverses myocardial fibrosis in a transgenic mouse model of human hypertrophic cardiomyopathy. Circulation 2001;103:789-91.

143. Lim WW, Baumert M, Neo M et al. Slowed atrial and atrioventricular conduction and depressed HRV in a murine model of hypertrophic cardiomyopathy. Clin Exp Pharmacol Physiol 2016;43:95-101.

144. Lim WW, Neo M, Thanigaimani S et al. Electrophysiological and Structural Remodeling of the Atria in a Mouse Model of Troponin-I Mutation Linked Hypertrophic Cardiomyopathy: Implications for Atrial Fibrillation. Int J Mol Sci 2021;22.

145. Liu S, Geng B, Zou L et al. Development of hypertrophic cardiomyopathy in perilipin-1 null mice with adipose tissue dysfunction. Cardiovasc Res 2015;105:20-30.

146. Liu Y, Afzal J, Vakrou S et al. Differences in microRNA-29 and Pro-fibrotic Gene Expression in Mouse and Human Hypertrophic Cardiomyopathy. Front Cardiovasc Med 2019;6:170.

147. Lombardi R, Bell A, Senthil V et al. Differential interactions of thin filament proteins in two cardiac troponin T mouse models of hypertrophic and dilated cardiomyopathies. Cardiovasc Res 2008;79:109-17.

148. Lowey S, Lesko LM, Rovner AS et al. Functional effects of the hypertrophic cardiomyopathy R403Q mutation are different in an alpha- or beta-myosin heavy chain backbone. J Biol Chem 2008;283:20579-89.

149. Lu D, Dong W, Zhang X et al. WIF1 causes dysfunction of heart in transgenic mice. Transgenic Res 2013;22:1179-89.

150. Lu D, Wang J, Li J et al. Meox1 accelerates myocardial hypertrophic decompensation through Gata4. Cardiovasc Res 2018;114:300-311.

151. Lucas DT, Aryal P, Szweda LI, Koch WJ, Leinwand LA. Alterations in mitochondrial function in a mouse model of hypertrophic cardiomyopathy. Am J Physiol Heart Circ Physiol 2003;284:H575-83.

152. Luckey SW, Mansoori J, Fair K, Antos CL, Olson EN, Leinwand LA. Blocking cardiac growth in hypertrophic cardiomyopathy induces cardiac dysfunction and decreased survival only in males. Am J Physiol Heart Circ Physiol 2007;292:H838-45.

153. Luckey SW, Walker LA, Smyth T et al. The role of Akt/GSK-3beta signaling in familial hypertrophic cardiomyopathy. J Mol Cell Cardiol 2009;46:739-47.

154. Luczak ED, Barthel KK, Stauffer BL, Konhilas JP, Cheung TH, Leinwand LA. Remodeling the cardiac transcriptional landscape with diet. Physiol Genomics 2011;43:772-80.

155. Luo HC, Pozios I, Vakrou S, Sorensen L, Abraham RM, Abraham T. Age-related changes in familial hypertrophic cardiomyopathy phenotype in transgenic mice and humans. J Huazhong Univ Sci Technolog Med Sci 2014;34:634-639.

156. Lutucuta S, Tsybouleva N, Ishiyama M et al. Induction and reversal of cardiac phenotype of human hypertrophic cardiomyopathy mutation cardiac troponin T-Q92 in switch on-switch off bigenic mice. Journal of the American College of Cardiology 2004;44:2221-2230.

157. Ma S, Jiang W, Liu X et al. Efficient Correction of a Hypertrophic Cardiomyopathy Mutation by ABEmax-NG. Circ Res 2021;129:895-908.

158. Maass AH, Ikeda K, Oberdorf-Maass S, Maier SK, Leinwand LA. Hypertrophy, fibrosis, and sudden cardiac death in response to pathological stimuli in mice with mutations in cardiac troponin T. Circulation 2004;110:2102-9.

159. Magida JA, Leinwand LA. Metabolic crosstalk between the heart and liver impacts familial hypertrophic cardiomyopathy. EMBO Mol Med 2014;6:482-95.

160. Mamidi R, Gresham KS, Li A, dos Remedios CG, Stelzer JE. Molecular effects of the myosin activator omecamtiv mecarbil on contractile properties of skinned myocardium lacking cardiac myosin binding protein-C. J Mol Cell Cardiol 2015;85:262-72.

161. Mamidi R, Li J, Doh CY, Verma S, Stelzer JE. Impact of the Myosin Modulator Mavacamten on Force Generation and Cross-Bridge Behavior in a Murine Model of Hypercontractility. J Am Heart Assoc 2018;7:e009627.

162. Marian AJ, Senthil V, Chen SN, Lombardi R. Antifibrotic effects of antioxidant N-acetylcysteine in a mouse model of human hypertrophic cardiomyopathy mutation. J Am Coll Cardiol 2006;47:827-34.

163. Martins AS, Parvatiyar MS, Feng HZ et al. In Vivo Analysis of Troponin C Knock-In (A8V) Mice: Evidence that TNNC1 Is a Hypertrophic Cardiomyopathy Susceptibility Gene. Circ Cardiovasc Genet 2015;8:653-664.

164. McConnell BK, Fatkin D, Semsarian C et al. Comparison of two murine models of familial hypertrophic cardiomyopathy. Circ Res 2001;88:383-9.

165. McKee LAK, Chen H, Regan JA et al. Sexually dimorphic myofilament function and cardiac troponin i phosphospecies distribution in hypertrophic cardiomyopathy mice. Archives of Biochemistry and Biophysics 2013;535:39-48.

166. Mearini G, Stimpel D, Krämer E et al. Repair of Mybpc3 mRNA by 5′-trans-splicing in a mouse model of hypertrophic cardiomyopathy. Molecular Therapy - Nucleic Acids 2013;2.

167. Mearini G, Stimpel D, Geertz B et al. Mybpc3 gene therapy for neonatal cardiomyopathy enables long-term disease prevention in mice. Nature Communications 2014;5.

168. Meng Q, Bhandary B, Bhuiyan MS et al. Myofibroblast-Specific TGFβ Receptor II Signaling in the Fibrotic Response to Cardiac Myosin Binding Protein C-Induced Cardiomyopathy. Circ Res 2018;123:1285-1297.

169. Merkulov S, Chen X, Chandler MP, Stelzer JE. In vivo cardiac myosin binding protein C gene transfer rescues myofilament contractile dysfunction in cardiac myosin binding protein C null mice. Circ Heart Fail 2012;5:635-44.

170. Messer AE, Bayliss CR, El-Mezgueldi M et al. Mutations in troponin T associated with Hypertrophic Cardiomyopathy increase Ca(2+)-sensitivity and suppress the modulation of Ca(2+)-sensitivity by troponin I phosphorylation. Arch Biochem Biophys 2016;601:113-20.

171. Michele DE, Gomez CA, Hong KE, Westfall MV, Metzger JM. Cardiac dysfunction in hypertrophic cardiomyopathy mutant tropomyosin mice is transgene-dependent, hypertrophy-independent, and improved by beta-blockade. Circ Res 2002;91:255-62.

172. Mijailovich SM, Prodanovic M, Poggesi C et al. The effect of variable troponin C mutation thin filament incorporation on cardiac muscle twitch contractions. J Mol Cell Cardiol 2021;155:112-124.

173. Miller T, Szczesna D, Housmans PR et al. Abnormal contractile function in transgenic mice expressing a familial hypertrophic cardiomyopathy-linked troponin T (I79N) mutation. J Biol Chem 2001;276:3743-55.

174. Montgomery DE, Tardiff JC, Chandra M. Cardiac troponin T mutations: Correlation between the type of mutation and the nature of myofilament dysfunction in transgenic mice. Journal of Physiology 2001;536:583-592.

175. Moore RK, Grinspan LT, Jimenez J, Guinto PJ, Ertz-Berger B, Tardiff JC. HCM-linked Delta 160E cardiac troponin T mutation causes unique progressive structural and molecular ventricular remodeling in transgenic mice. Journal of Molecular and Cellular Cardiology 2013;58:188-198.

176. Moore RK, Abdullah S, Tardiff JC. Allosteric effects of cardiac troponin TNT1 mutations on actomyosin binding: a novel pathogenic mechanism for hypertrophic cardiomyopathy. Arch Biochem Biophys 2014;552:21-8.

177. Musicante M, Kim HH, Chen Y et al. Regulation of endothelial nitric oxide synthase in cardiac remodeling. Int J Cardiol 2022;364:96-101.

178. Muthu P, Mettikolla P, Calander N et al. Single molecule kinetics in the familial hypertrophic cardiomyopathy D166V mutant mouse heart. Journal of Molecular and Cellular Cardiology 2010;48:989-998.

179. Muthu P, Wang L, Yuan CC et al. Structural and functional aspects of the myosin essential light chain in cardiac muscle contraction. Faseb j 2011;25:4394-405.

180. Muthu P, Kazmierczak K, Jones M, Szczesna-Cordary D. The effect of myosin RLC phosphorylation in normal and cardiomyopathic mouse hearts. Journal of cellular and molecular medicine 2012;16:911-919.

181. Muthuchamy M, Pieples K, Rethinasamy P et al. Mouse model of a familial hypertrophic cardiomyopathy mutation in alpha-tropomyosin manifests cardiac dysfunction. Circ Res 1999;85:47-56.

182. Najafi A, Schlossarek S, van Deel ED et al. Sexual dimorphic response to exercise in hypertrophic cardiomyopathy-associated MYBPC3-targeted knock-in mice. Pflugers Arch 2015;467:1303-17.

183. Najafi A, Sequeira V, Helmes M et al. Selective phosphorylation of PKA targets after β-adrenergic receptor stimulation impairs myofilament function in Mybpc3-targeted HCM mouse model. Cardiovasc Res 2016;110:200-14.

184. Nebigil CG, Jaffré F, Messaddeq N et al. Overexpression of the serotonin 5-HT2B receptor in heart leads to abnormal mitochondrial function and cardiac hypertrophy. Circulation 2003;107:3223-9.

185. Nguyen L, Chung J, Lam L, Tsoutsman T, Semsarian C. Abnormal cardiac response to exercise in a murine model of familial hypertrophic cardiomyopathy. Int J Cardiol 2007;119:245-8.

186. Oberst L, Zhao G, Park JT et al. Dominant-negative effect of a mutant cardiac troponin T on cardiac structure and function in transgenic mice. Journal of Clinical Investigation 1998;102:1498-1505.

187. Ohsawa Y, Toko H, Katsura M et al. Overexpression of P104L mutant caveolin-3 in mice develops hypertrophic cardiomyopathy with enhanced contractility in association with increased endothelial nitric oxide synthase activity. Hum Mol Genet 2004;13:151-7.

188. Okuda S, Sufu-Shimizu Y, Kato T et al. CaMKII-mediated phosphorylation of RyR2 plays a crucial role in aberrant Ca(2+) release as an arrhythmogenic substrate in cardiac troponin T-related familial hypertrophic cardiomyopathy. Biochem Biophys Res Commun 2018;496:1250-1256.

189. Olsson MC, Palmer BM, Leinwand LA, Moore RL. Gender and aging in a transgenic mouse model of hypertrophic cardiomyopathy. Am J Physiol Heart Circ Physiol 2001;280:H1136-44.

190. Olsson MC, Palmer BM, Stauffer BL, Leinwand LA, Moore RL. Morphological and functional alterations in ventricular myocytes from male transgenic mice with hypertrophic cardiomyopathy. Circ Res 2004;94:201-7.

191. Pal S, Nixon BR, Glennon MS et al. Replication Stress Response Modifies Sarcomeric Cardiomyopathy Remodeling. J Am Heart Assoc 2021;10:e021768.

192. Palmer BM, Fishbaugher DE, Schmitt JP et al. Differential cross-bridge kinetics of FHC myosin mutations R403Q and R453C in heterozygous mouse myocardium. Am J Physiol Heart Circ Physiol 2004;287:H91-9.

193. Palmer BM, Wang Y, Teekakirikul P et al. Myofilament mechanical performance is enhanced by R403Q myosin in mouse myocardium independent of sex. American Journal of Physiology - Heart and Circulatory Physiology 2008;294:H1939-H1947.

194. Peña JR, Szkudlarek AC, Warren CM et al. Neonatal gene transfer of Serca2a delays onset of hypertrophic remodeling and improves function in familial hypertrophic cardiomyopathy. J Mol Cell Cardiol 2010;49:993-1002.

195. Petersen HH, Choy J, Stauffer B et al. Coronary artery myogenic response in a genetic model of hypertrophic cardiomyopathy. Am J Physiol Heart Circ Physiol 2002;283:H2244-9.

196. Pioner JM, Vitale G, Gentile F et al. Genotype-Driven Pathogenesis of Atrial Fibrillation in Hypertrophic Cardiomyopathy: The Case of Different TNNT2 Mutations. Front Physiol 2022;13:864547.

197. Pohlmann L, Kröger I, Vignier N et al. Cardiac myosin-binding protein C is required for complete relaxation in intact myocytes. Circulation Research 2007;101:928-938.

198. Prabhakar R, Boivin GP, Grupp IL et al. A familial hypertrophic cardiomyopathy α-tropomyosin mutation causes severe cardiac hypertrophy and death in mice. Journal of Molecular and Cellular Cardiology 2001;33:1815-1828.

199. Prabhakar R, Petrashevskaya N, Schwartz A et al. A mouse model of familial hypertrophic cardiomyopathy caused by a alpha-tropomyosin mutation. Mol Cell Biochem 2003;251:33-42.

200. Prasad V, Lorenz JN, Lasko VM, Nieman ML, Al Moamen NJ, Shull GE. Loss of the AE3 Cl(-)/HCO(-) 3 exchanger in mice affects rate-dependent inotropy and stress-related AKT signaling in heart. Front Physiol 2013;4:399.

201. Prasad V, Lorenz JN, Lasko VM et al. Ablation of plasma membrane Ca(2+)-ATPase isoform 4 prevents development of hypertrophy in a model of hypertrophic cardiomyopathy. J Mol Cell Cardiol 2014;77:53-63.

202. Purevjav E, Arimura T, Augustin S et al. Molecular basis for clinical heterogeneity in inherited cardiomyopathies due to myopalladin mutations. Hum Mol Genet 2012;21:2039-53.

203. Qi L, Yu Y, Chi X et al. Depletion of Kindlin-2 induces cardiac dysfunction in mice. Science China Life sciences 2016;59:1123-1130.

204. Quick AP, Landstrom AP, Wang Q et al. Novel junctophilin-2 mutation A405S is associated with basal septal hypertrophy and diastolic dysfunction. JACC Basic Transl Sci 2017;2:56-67.

205. Raja AA, Wakimoto H, DeLaughter DM et al. Ablation of lysophosphatidic acid receptor 1 attenuates hypertrophic cardiomyopathy in a mouse model. Proc Natl Acad Sci U S A 2022;119:e2204174119.

206. Rajamannan NM, Subramaniam M, Abraham TP et al. TGFβ inducible early gene-1 (TIEG1) and cardiac hypertrophy: Discovery and characterization of a novel signaling pathway. Journal of Cellular Biochemistry 2007;100:315-325.

207. Rajan S, Williams SS, Jagatheesan G et al. Microarray analysis of gene expression during early stages of mild and severe cardiac hypertrophy. Physiol Genomics 2006;27:309-17.

208. Rajan S, Pena JR, Jegga AG, Aronow BJ, Wolska BM, Wieczorek DF. Microarray analysis of active cardiac remodeling genes in a familial hypertrophic cardiomyopathy mouse model rescued by a phospholamban knockout. Physiol Genomics 2013;45:764-73.

209. Ramirez-Correa GA, Frazier AH, Zhu GS et al. Cardiac troponin I Pro82Ser variant induces diastolic dysfunction, blunts beta-adrenergic response, and impairs myofilament cooperativity. Journal of Applied Physiology 2015;118:212-223.

210. Rasicci DV, Kirkland O, Moonschi FH et al. Impact of regulatory light chain mutation K104E on the ATPase and motor properties of cardiac myosin. J Gen Physiol 2021;153.

211. Reichart D, Newby GA, Wakimoto H et al. Efficient in vivo genome editing prevents hypertrophic cardiomyopathy in mice. Nat Med 2023;29:412-421.

212. Rice R, Guinto P, Dowell-Martino C et al. Cardiac myosin heavy chain isoform exchange alters the phenotype of cTnT-related cardiomyopathies in mouse hearts. J Mol Cell Cardiol 2010;48:979-88.

213. Rixon C, Andreassen K, Shen X et al. Lumican accumulates with fibrillar collagen in fibrosis in hypertrophic cardiomyopathy. ESC Heart Fail 2023;10:858-871.

214. Rowlands CT, Owen T, Lawal S et al. Age- and strain-related aberrant Ca(2+) release is associated with sudden cardiac death in the ACTC E99K mouse model of hypertrophic cardiomyopathy. Am J Physiol Heart Circ Physiol 2017;313:H1213-h1226.

215. Ruan H, Mitchell S, Vainoriene M et al. Gi alpha 1-mediated cardiac electrophysiological remodeling and arrhythmia in hypertrophic cardiomyopathy. Circulation 2007;116:596-605.

216. Ruggiero A, Chen SN, Lombardi R, Rodriguez G, Marian AJ. Pathogenesis of hypertrophic cardiomyopathy caused by myozenin 2 mutations is independent of calcineurin activity. Cardiovasc Res 2013;97:44-54.

217. Ruiz M, Khairallah M, Dingar D et al. MK2-Deficient Mice Are Bradycardic and Display Delayed Hypertrophic Remodeling in Response to a Chronic Increase in Afterload. J Am Heart Assoc 2021;10:e017791.

218. Ryba DM, Warren CM, Karam CN et al. Sphingosine-1-Phosphate Receptor Modulator, FTY720, Improves Diastolic Dysfunction and Partially Reverses Atrial Remodeling in a Tm-E180G Mouse Model Linked to Hypertrophic Cardiomyopathy. Circ Heart Fail 2019;12:e005835.

219. Sanbe A, Nelson D, Gulick J et al. In vivo analysis of an essential myosin light chain mutation linked to familial hypertrophic cardiomyopathy. Circ Res 2000;87:296-302.

220. Schlossarek S, Englmann DR, Sultan KR, Sauer M, Eschenhagen T, Carrier L. Defective proteolytic systems in Mybpc3-targeted mice with cardiac hypertrophy. Basic Res Cardiol 2012;107:235.

221. Schlossarek S, Schuermann F, Geertz B, Mearini G, Eschenhagen T, Carrier L. Adrenergic stress reveals septal hypertrophy and proteasome impairment in heterozygous Mybpc3-targeted knock-in mice. J Muscle Res Cell Motil 2012;33:5-15.

222. Schlossarek S, Singh SR, Geertz B et al. Proteasome inhibition slightly improves cardiac function in mice with hypertrophic cardiomyopathy. Front Physiol 2014;5:484.

223. Schmitt JP, Semsarian C, Arad M et al. Consequences of pressure overload on sarcomere protein mutation-induced hypertrophic cardiomyopathy. Circulation 2003;108:1133-8.

224. Schober T, Huke S, Venkataraman R et al. Myofilament Ca sensitization increases cytosolic Ca binding affinity, alters intracellular Ca homeostasis, and causes pause-dependent Ca-triggered arrhythmia. Circ Res 2012;111:170-9.

225. Schuldt M, Pei J, Harakalova M et al. Proteomic and Functional Studies Reveal Detyrosinated Tubulin as Treatment Target in Sarcomere Mutation-Induced Hypertrophic Cardiomyopathy. Circ Heart Fail 2021;14:e007022.

226. Schulz EM, Wilder T, Chowdhury SA et al. Decreasing tropomyosin phosphorylation rescues tropomyosin-induced familial hypertrophic cardiomyopathy. J Biol Chem 2013;288:28925-35.

227. Semsarian C, Healey MJ, Fatkin D et al. A polymorphic modifier gene alters the hypertrophic response in a murine model of familial hypertrophic cardiomyopathy. J Mol Cell Cardiol 2001;33:2055-60.

228. Semsarian C, Ahmad I, Giewat M et al. The L-type calcium channel inhibitor diltiazem prevents cardiomyopathy in a mouse model. J Clin Invest 2002;109:1013-20.

229. Singh SR, Zech ATL, Geertz B et al. Activation of Autophagy Ameliorates Cardiomyopathy in Mybpc3-Targeted Knockin Mice. Circ Heart Fail 2017;10.

230. Sirenko SG, Potter JD, Knollmann BC. Differential effect of troponin T mutations on the inotropic responsiveness of mouse hearts--role of myofilament Ca2+ sensitivity increase. J Physiol 2006;575:201-13.

231. Sitbon YH, Kazmierczak K, Liang J et al. Ablation of the N terminus of cardiac essential light chain promotes the super-relaxed state of myosin and counteracts hypercontractility in hypertrophic cardiomyopathy mutant mice. Febs j 2020.

232. Sitbon YH, Diaz F, Kazmierczak K, Liang J, Wangpaichitr M, Szczesna-Cordary D. Cardiomyopathic mutations in essential light chain reveal mechanisms regulating the super relaxed state of myosin. J Gen Physiol 2021;153.

233. Song Q, Schmidt AG, Hahn HS et al. Rescue of cardiomyocyte dysfunction by phospholamban ablation does not prevent ventricular failure in genetic hypertrophy. J Clin Invest 2003;111:859-67.

234. Song W, Dyer E, Stuckey DJ et al. Molecular mechanism of the E99K mutation in cardiac actin (ACTC Gene) that causes apical hypertrophy in man and mouse. J Biol Chem 2011;286:27582-93.

235. Song W, Vikhorev PG, Kashyap MN et al. Mechanical and energetic properties of papillary muscle from ACTC E99K transgenic mouse models of hypertrophic cardiomyopathy. Am J Physiol Heart Circ Physiol 2013;304:H1513-24.

236. Song B, Dang H, Dong R. The role and the signal pathways of Yes-associated protein 2 in hypertrophic cardiomyopathy. Gen Physiol Biophys 2021;40:419-426.

237. Sørensen LL, Bedja D, Sysa-Shah P et al. Echocardiographic Characterization of a Murine Model of Hypertrophic Obstructive Cardiomyopathy Induced by Cardiac-specific Overexpression of Epidermal Growth Factor Receptor 2. Comp Med 2016;66:268-77.

238. Spindler M, Saupe KW, Christe ME et al. Diastolic dysfunction and altered energetics in the alphaMHC403/+ mouse model of familial hypertrophic cardiomyopathy. J Clin Invest 1998;101:1775-83.

239. Stachowski-Doll MJ, Papadaki M, Martin TG et al. GSK-3beta Localizes to the Cardiac Z-Disc to Maintain Length Dependent Activation. Circ Res 2022;130:871-886.

240. Stathopoulou K, Schnittger J, Raabe J et al. CMYA5 is a novel interaction partner of FHL2 in cardiac myocytes. FEBS J 2022;289:4622-4645.

241. Stauffer BL, Konhilas JP, Luczak ED, Leinwand LA. Soy diet worsens heart disease in mice. J Clin Invest 2006;116:209-16.

242. Stelzer JE, Dunning SB, Moss RL. Ablation of cardiac myosin-binding protein-C accelerates stretch activation in murine skinned myocardium. Circulation Research 2006;98:1212-1218.

243. Stöhr A, Friedrich FW, Flenner F et al. Contractile abnormalities and altered drug response in engineered heart tissue from Mybpc3-targeted knock-in mice. J Mol Cell Cardiol 2013;63:189-98.

244. Stücker S, Kresin N, Carrier L, Friedrich FW. Nebivolol Desensitizes Myofilaments of a Hypertrophic Cardiomyopathy Mouse Model. Front Physiol 2017;8:558.

245. Sucharov CC, Mariner P, Long C, Bristow M, Leinwand L. Yin Yang 1 is increased in human heart failure and represses the activity of the human alpha-myosin heavy chain promoter. J Biol Chem 2003;278:31233-9.

246. Sussman MA, Lim HW, Gude N et al. Prevention of cardiac hypertrophy in mice by calcineurin inhibition. Science 1998;281:1690-3.

247. Sysa-Shah P, Xu Y, Guo X et al. Cardiac-specific over-expression of epidermal growth factor receptor 2 (ErbB2) induces pro-survival pathways and hypertrophic cardiomyopathy in mice. PLoS One 2012;7:e42805.

248. Szczesna-Cordary D, Guzman G, Zhao J, Hernandez O, Wei J, Diaz-Perez Z. The E22K mutation of myosin RLC that causes familial hypertrophic cardiomyopathy increases calcium sensitivity of force and ATPase in transgenic mice. J Cell Sci 2005;118:3675-83.

249. Szczesna-Cordary D, Jones M, Moore JR et al. Myosin regulatory light chain E22K mutation results in decreased cardiac intracellular calcium and force transients. Faseb j 2007;21:3974-85.

250. Tanaka A, Yuasa S, Mearini G et al. Endothelin-1 induces myofibrillar disarray and contractile vector variability in hypertrophic cardiomyopathy-induced pluripotent stem cell-derived cardiomyocytes. J Am Heart Assoc 2014;3:e001263.

251. Tardiff JC, Factor SM, Tompkins BD et al. A truncated cardiac troponin T molecule in transgenic mice suggests multiple cellular mechanisms for familial hypertrophic cardiomyopathy. Journal of Clinical Investigation 1998;101:2800-2811.

252. Tardiff JC, Hewett TE, Palmer BM et al. Cardiac troponin T mutations result in allele-specific phenotypes in a mouse model for hypertrophic cardiomyopathy. J Clin Invest 1999;104:469-81.

253. Teekakirikul P, Eminaga S, Toka O et al. Cardiac fibrosis in mice with hypertrophic cardiomyopathy is mediated by non-myocyte proliferation and requires Tgf-β. J Clin Invest 2010;120:3520-9.

254. Thottakara T, Friedrich FW, Reischmann S et al. The E3 ubiquitin ligase Asb2β is downregulated in a mouse model of hypertrophic cardiomyopathy and targets desmin for proteasomal degradation. J Mol Cell Cardiol 2015;87:214-24.

255. Toepfer CN, Garfinkel AC, Venturini G et al. Myosin Sequestration Regulates Sarcomere Function, Cardiomyocyte Energetics, and Metabolism, Informing the Pathogenesis of Hypertrophic Cardiomyopathy. Circulation 2020;141:828-842.

256. Toib A, Zhang C, Borghetti G et al. Remodeling of repolarization and arrhythmia susceptibility in a myosin-binding protein C knockout mouse model. Am J Physiol Heart Circ Physiol 2017;313:H620-h630.

257. Tong CW, Stelzer JE, Greaser ML, Powers PA, Moss RL. Acceleration of Crossbridge Kinetics by Protein Kinase A Phosphorylation of Cardiac Myosin Binding Protein C Modulates Cardiac Function. Circulation Research 2008;103:974-U166.

258. Tsoutsman T, Chung J, Doolan A et al. Molecular insights from a novel cardiac troponin I mouse model of familial hypertrophic cardiomyopathy. J Mol Cell Cardiol 2006;41:623-32.

259. Tsoutsman T, Kelly M, Ng DC et al. Severe heart failure and early mortality in a double-mutation mouse model of familial hypertrophic cardiomyopathy. Circulation 2008;117:1820-31.

260. Tsoutsman T, Wang X, Garchow K, Riser B, Twigg S, Semsarian C. CCN2 plays a key role in extracellular matrix gene expression in severe hypertrophic cardiomyopathy and heart failure. J Mol Cell Cardiol 2013;62:164-78.

261. Tsybouleva N, Zhang L, Chen S et al. Aldosterone, through novel signaling proteins, is a fundamental molecular bridge between the genetic defect and the cardiac phenotype of hypertrophic cardiomyopathy. Circulation 2004;109:1284-91.

262. Tucci S, Flögel U, Hermann S, Sturm M, Schäfers M, Spiekerkoetter U. Development and pathomechanisms of cardiomyopathy in very long-chain acyl-CoA dehydrogenase deficient (VLCAD(-/-)) mice. Biochim Biophys Acta 2014;1842:677-85.

263. Tyska MJ, Hayes E, Giewat M, Seidman CE, Seidman JG, Warshaw DM. Single-molecule mechanics of R403Q cardiac myosin isolated from the mouse model of familial hypertrophic cardiomyopathy. Circ Res 2000;86:737-44.

264. Vakrou S, Fukunaga R, Foster DB et al. Allele-specific differences in transcriptome, miRNome, and mitochondrial function in two hypertrophic cardiomyopathy mouse models. JCI Insight 2018;3.

265. Vakrou S, Liu Y, Zhu L et al. Differences in molecular phenotype in mouse and human hypertrophic cardiomyopathy. Sci Rep 2021;11:13163.

266. Van Sligtenhorst I, Ding ZM, Shi ZZ, Read RW, Hansen G, Vogel P. Cardiomyopathy in alpha-Kinase 3 (ALPK3)-Deficient Mice. Veterinary Pathology 2012;49:131-141.

267. Vignier N, Schlossarek S, Fraysse B et al. Nonsense-mediated mRNA decay and ubiquitin-proteasome system regulate cardiac myosin-binding protein C mutant levels in cardiomyopathic mice. Circ Res 2009;105:239-48.

268. Vignier N, Le Corvoisier P, Blard C et al. AT1 blockade abolishes left ventricular hypertrophy in heterozygous cMyBP-C null mice: role of FHL1. Fundam Clin Pharmacol 2014;28:249-56.

269. Vikstrom KL, Factor SM, Leinwand LA. Mice expressing mutant myosin heavy chains are a model for familial hypertrophic cardiomyopathy. Mol Med 1996;2:556-67.

270. Vikstrom KL, Bohlmeyer T, Factor SM, Leinwand LA. Hypertrophy, pathology, and molecular markers of cardiac pathogenesis. Circ Res 1998;82:773-8.

271. Viola H, Johnstone V, Cserne Szappanos H et al. The L-type Ca(2+) channel facilitates abnormal metabolic activity in the cTnI-G203S mouse model of hypertrophic cardiomyopathy. J Physiol 2016;594:4051-70.

272. Viola HM, Johnstone VPA, Cserne Szappanos H et al. The Role of the L-Type Ca(2+) Channel in Altered Metabolic Activity in a Murine Model of Hypertrophic Cardiomyopathy. JACC Basic Transl Sci 2016;1:61-72.

273. Viola HM, Shah AA, Johnstone VPA, Cserne Szappanos H, Hodson MP, Hool LC. Characterization and validation of a preventative therapy for hypertrophic cardiomyopathy in a murine model of the disease. Proc Natl Acad Sci U S A 2020;117:23113-23124.

274. Viola HM, Richworth C, Solomon T et al. A maladaptive feedback mechanism between the extracellular matrix and cytoskeleton contributes to hypertrophic cardiomyopathy pathophysiology. Commun Biol 2023;6:4.

275. Wang Y, Xu Y, Kerrick WGL et al. Prolonged Ca2+ and Force Transients in Myosin RLC Transgenic Mouse Fibers Expressing Malignant and Benign FHC Mutations. Journal of Molecular Biology 2006;361:286-299.

276. Wang TT, Kwon HS, Dai G et al. Resolving myoarchitectural disarray in the mouse ventricular wall with diffusion spectrum magnetic resonance imaging. Ann Biomed Eng 2010;38:2841-50.

277. Wang Y, Pinto JR, Solis RS et al. Generation and functional characterization of knock-in mice harboring the cardiac troponin I-R21C mutation associated with hypertrophic cardiomyopathy. J Biol Chem 2012;287:2156-67.

278. Wang L, Muthu P, Szczesna-Cordary D, Kawai M. Diversity and similarity of motor function and cross-bridge kinetics in papillary muscles of transgenic mice carrying myosin regulatory light chain mutations D166V and R58Q. Journal of Molecular and Cellular Cardiology 2013;62:153-163.

279. Wang L, Bai F, Zhang Q, Song W, Messer A, Kawai M. Development of apical hypertrophic cardiomyopathy with age in a transgenic mouse model carrying the cardiac actin E99K mutation. J Muscle Res Cell Motil 2017;38:421-435.

280. Wang L, Kazmierczak K, Yuan CC, Yadav S, Kawai M, Szczesna-Cordary D. Cardiac contractility, motor function, and cross-bridge kinetics in N47K-RLC mutant mice. Febs j 2017;284:1897-1913.

281. Wang Y, Yuan CC, Kazmierczak K, Szczesna-Cordary D, Burghardt TP. Single cardiac ventricular myosins are autonomous motors. Open Biol 2018;8.

282. Wang H, Lin Y, Zhang R et al. Programmed Exercise Attenuates Familial Hypertrophic Cardiomyopathy in Transgenic E22K Mice via Inhibition of PKC-alpha/NFAT Pathway. Front Cardiovasc Med 2022;9:808163.

283. Watson PA, Reusch JE, McCune SA et al. Restoration of CREB function is linked to completion and stabilization of adaptive cardiac hypertrophy in response to exercise. Am J Physiol Heart Circ Physiol 2007;293:H246-59.

284. Wei BR, Simpson RM, Johann DJ et al. Proteomic profiling of H-Ras-G12V induced hypertrophic cardiomyopathy in transgenic mice using comparative LC-MS analysis of thin fresh-frozen tissue sections. J Proteome Res 2012;11:1561-70.

285. Welikson RE, Buck SH, Patel JR et al. Cardiac myosin heavy chains lacking the light chain binding domain cause hypertrophic cardiomyopathy in mice. American Journal of Physiology - Heart and Circulatory Physiology 1999;276:H2148-H2158.

286. Wen Y, Pinto JR, Gomes AV et al. Functional consequences of the human cardiac troponin I hypertrophic cardiomyopathy mutation R145G in transgenic mice. Journal of Biological Chemistry 2008;283:20484-20494.

287. Westermann D, Knollmann BC, Steendijk P et al. Diltiazem treatment prevents diastolic heart failure in mice with familial hypertrophic cardiomyopathy. Eur J Heart Fail 2006;8:115-21.

288. Wilder T, Ryba DM, Wieczorek DF, Wolska BM, Solaro RJ. N-acetylcysteine reverses diastolic dysfunction and hypertrophy in familial hypertrophic cardiomyopathy. Am J Physiol Heart Circ Physiol 2015;309:H1720-30.

289. Willis MS, Wadosky KM, Rodríguez JE et al. Muscle ring finger 1 and muscle ring finger 2 are necessary but functionally redundant during developmental cardiac growth and regulate E2F1-mediated gene expression in vivo. Cell Biochem Funct 2014;32:39-50.

290. Witt CC, Gerull B, Davies MJ, Centner T, Linke WA, Thierfelder L. Hypercontractile properties of cardiac muscle fibers in a knock-in mouse model of cardiac myosin-binding protein-C. J Biol Chem 2001;276:5353-9.

291. Wolf CM, Moskowitz IP, Arno S et al. Somatic events modify hypertrophic cardiomyopathy pathology and link hypertrophy to arrhythmia. Proc Natl Acad Sci U S A 2005;102:18123-8.

292. Xu X, Roe ND, Weiser-Evans MC, Ren J. Inhibition of mammalian target of rapamycin with rapamycin reverses hypertrophic cardiomyopathy in mice with cardiomyocyte-specific knockout of PTEN. Hypertension 2014;63:729-39.

293. Xu M, Bermea KC, Ayati M et al. Alteration in tyrosine phosphorylation of cardiac proteome and EGFR pathway contribute to hypertrophic cardiomyopathy. Commun Biol 2022;5:1251.

294. Yadav S, Kazmierczak K, Liang J, Sitbon YH, Szczesna-Cordary D. Phosphomimetic-mediated in vitro rescue of hypertrophic cardiomyopathy linked to R58Q mutation in myosin regulatory light chain. Febs j 2019;286:151-168.

295. Yadav S, Yuan CC, Kazmierczak K et al. Therapeutic potential of AAV9-S15D-RLC gene delivery in humanized MYL2 mouse model of HCM. J Mol Med (Berl) 2019;97:1033-1047.

296. Yang Q, Sanbe A, Osinska H, Hewett TE, Klevitsky R, Robbins J. A mouse model of myosin binding protein C human familial hypertrophic cardiomyopathy. J Clin Invest 1998;102:1292-300.

297. Yang Q, Sanbe A, Osinska H, Hewett TE, Klevitsky R, Robbins J. In vivo modeling of myosin binding protein C familial hypertrophic cardiomyopathy. Circ Res 1999;85:841-7.

298. Yang Q, Osinska H, Klevitsky R, Robbins J. Phenotypic deficits in mice expressing a myosin binding protein C lacking the titin and myosin binding domains. J Mol Cell Cardiol 2001;33:1649-58.

299. Yuan CC, Muthu P, Kazmierczak K et al. Constitutive phosphorylation of cardiac myosin regulatory light chain prevents development of hypertrophic cardiomyopathy in mice. Proceedings of the National Academy of Sciences of the United States of America 2015;112:E4138-E4146.

300. Yuan CC, Kazmierczak K, Liang J, Ma W, Irving TC, Szczesna-Cordary D. Molecular basis of force-pCa relation in MYL2 cardiomyopathy mice: Role of the super-relaxed state of myosin. Proc Natl Acad Sci U S A 2022;119.

301. Zhang XM, Azhar G, Chai JY et al. Cardiomyopathy in transgenic mice with cardiacspecific overexpression of serum response factor. American Journal of Physiology-Heart and Circulatory Physiology 2001;280:H1782-H1792.

302. Zhang J, Wang L, Kazmierczak K, Yun H, Szczesna-Cordary D, Kawai M. Hypertrophic cardiomyopathy associated E22K mutation in myosin regulatory light chain decreases calcium-activated tension and stiffness and reduces myofilament Ca(2+) sensitivity. FEBS J 2021;288:4596-4613.

303. Zhao W, Zhao T, Chen Y et al. A Murine Hypertrophic Cardiomyopathy Model: The DBA/2J Strain. PLoS One 2015;10:e0133132.

304. Zheng M, Dilly K, Dos Santos Cruz J et al. Sarcoplasmic reticulum calcium defect in Ras-induced hypertrophic cardiomyopathy heart. Am J Physiol Heart Circ Physiol 2004;286:H424-33.

Supplemental References Cat models

1. Adin DB, Diley-Poston L. Papillary muscle measurements in cats with normal echocardiograms and cats with concentric left ventricular hypertrophy. Journal of Veterinary Internal Medicine 2007;21:737-741.

2. Argenta FF, Mello LS, Cony FG, Pavarini SP, Driemeier D, Sonne L. Epidemiological and pathological aspects of cardiomyopathies in cats in southern Brazil. Pesquisa Veterinaria Brasileira 2020;40:389-398.

3. Belerenian G, Donati PA, Rodriguez CD et al. Findings suggestive of coronary microvascular dysfunction in cats with myocardial ischemia. Open Vet J 2021;11:468-470.

4. Biasato I, Francescone L, La Rosa G, Tursi M. Anatomopathological staging of feline hypertrophic cardiomyopathy through quantitative evaluation based on morphometric and histopathological data. Research in Veterinary Science 2015;102:136-141.

5. Biondo AW, Ehrhart EJ, Sisson DD, Bulmer BJ, De Morais HS, Solter PF. Immunohistochemistry of atrial and brain natriuretic peptides in control cats and cats with hypertrophic cardiomyopathy. Vet Pathol 2003;40:501-6.

6. Borgeat K, Sherwood K, Payne JR, Luis Fuentes V, Connolly DJ. Plasma cardiac troponin I concentration and cardiac death in cats with hypertrophic cardiomyopathy. J Vet Intern Med 2014;28:1731-7.

7. Carlos Sampedrano C, Chetboul V, Mary J et al. Prospective Echocardiographic and Tissue Doppler Imaging Screening of a Population of Maine Coon Cats Tested for the A31P Mutation in the Myosin-Binding Protein C Gene: A Specific Analysis of the Heterozygous Status. Journal of Veterinary Internal Medicine 2009;23:91-99.

8. Cesta MF, Baty CJ, Keene BW, Smoak IW, Malarkey DE. Pathology of end-stage remodeling in a family of cats with hypertrophic cardiomyopathy. Veterinary Pathology 2005;42:458-467.

9. Cheng WC, Wilkie L, Kurosawa TA et al. Immunohistological Evaluation of Von Willebrand Factor in the Left Atrial Endocardium and Atrial Thrombi from Cats with Cardiomyopathy. Animals (Basel) 2021;11.

10. Chetboul V, Sampedrano CC, Gouni V, Nicolle AP, Pouchelon JL. Two-dimensional color tissue Doppler imaging detects myocardial dysfunction before occurrence of hypertrophy in a young Maine Coon cat. Veterinary Radiology & Ultrasound 2006;47:295-300.

11. Chetboul V, Petit A, Gouni V et al. Prospective echocardiographic and tissue Doppler screening of a large Sphynx cat population: Reference ranges, heart disease prevalence and genetic aspects. Journal of Veterinary Cardiology 2012;14:497-509.

12. Christiansen LB, Dela F, Koch J, Hansen CN, Leifsson PS, Yokota T. Impaired cardiac mitochondrial oxidative phosphorylation and enhanced mitochondrial oxidative stress in feline hypertrophic cardiomyopathy. American Journal of Physiology-Heart and Circulatory Physiology 2015;308:H1237-H1247.

13. Christiansen LB, Prats C, Hyttel P, Koch J. Ultrastructural myocardial changes in seven cats with spontaneous hypertrophic cardiomyopathy. Journal of Veterinary Cardiology 2015;17:S220-S232.

14. Connolly DJ, Cannata J, Boswood A, Archer J, Groves EA, Neiger R. Cardiac troponin I in cats with hypertrophic cardiomyopathy. Journal of Feline Medicine and Surgery 2003;5:209-216.

15. den Toom ML, van Leeuwen MW, Szatmári V, Teske E. Effects of clopidogrel therapy on whole blood platelet aggregation, the Plateletworks® assay and coagulation parameters in cats with asymptomatic hypertrophic cardiomyopathy: a pilot study. Vet Q 2017;37:8-15.

16. Ferasin L, Sturgess CP, Cannon MJ, Caney SMA, Gruffydd-Jones TJ, Wotton PR. Feline idiopathic cardiomyopathy: a retrospective study of 106 cats (1994-2001). Journal of Feline Medicine and Surgery 2003;5:151-159.

17. Fonfara S, Kitz S, Hetzel U, Kipar A. Myocardial leptin transcription in feline hypertrophic cardiomyopathy. Res Vet Sci 2017;112:105-108.

18. Fonfara S, Kitz S, Monteith G, Hahn S, Kipar A. Myocardial transcription of inflammatory and remodeling markers in cats with hypertrophic cardiomyopathy and

systemic diseases associated with an inflammatory phenotype. Res Vet Sci 2021;136:484-494.

19. Fox PR, Liu SK, Maron BJ. Echocardiographic assessment of spontaneously occurring feline hypertrophic cardiomyopathy. An animal model of human disease. Circulation 1995;92:2645-51.

20. Fox PR, Keene BW, Lamb K et al. International collaborative study to assess cardiovascular risk and evaluate long-term health in cats with preclinical hypertrophic cardiomyopathy and apparently healthy cats: The REVEAL Study. Journal of Veterinary Internal Medicine 2018;32:930-943.

21. Franchini A, Abbott JA, Lahmers S, Eriksson A. Clinical characteristics of cats referred for evaluation of subclinical cardiac murmurs. J Feline Med Surg 2021;23:708-714.

22. Freeman LM, Rush JE, Meurs KM, Bulmer BJ, Cunningham SM. Body size and metabolic differences in Maine Coon cats with and without hypertrophic cardiomyopathy. J Feline Med Surg 2013;15:74-80.

23. Freeman LM, Rush JE, Cunningham SM, Bulmer BJ. A Randomized Study Assessing the Effect of Diet in Cats with Hypertrophic Cardiomyopathy. Journal of Veterinary Internal Medicine 2014;28:847-856.

24. Fries RC, Kadotani S, Keating SCJ, Stack JP. Cardiac extracellular volume fraction in cats with preclinical hypertrophic cardiomyopathy. J Vet Intern Med 2021;35:812-822.

25. Fries RC, Kadotani S, Stack JP, Kruckman L, Wallace G. Prognostic Value of Neutrophil-to-Lymphocyte Ratio in Cats With Hypertrophic Cardiomyopathy. Front Vet Sci 2022;9:813524.

26. Fujii Y, Masuda Y, Takashima K et al. Hypertrophic cardiomyopathy in two kittens. Journal of Veterinary Medical Science 2001;63:583-585.

27. Gavaghan BJ, Kittleson MD, Fisher KJ, Kass PH, Gavaghan MA. Quantification of left ventricular diastolic wall motion by Doppler tissue imaging in healthy cats and cats with cardiomyopathy. American Journal of Veterinary Research 1999;60:1478-1486.

28. Godiksen MTN, Granstrom S, Koch J, Christiansen M. Hypertrophic cardiomyopathy in young Maine Coon cats caused by the p.A31P cMyBP-C mutation - the clinical significance of having the mutation. Acta Veterinaria Scandinavica 2011;53.

29. Granstrom S, Godiksen MTN, Christiansen M, Pipper CB, Willesen JT, Koch J. Prevalence of Hypertrophic Cardiomyopathy in a Cohort of British Shorthair Cats in Denmark. Journal of Veterinary Internal Medicine 2011;25:866-871.

30. Granstrom S, Godiksen MTN, Christiansen M et al. Genotype-phenotype correlation between the cardiac myosin binding protein C mutation A31P and hypertrophic cardiomyopathy in a cohort of Maine Coon cats: a longitudinal study. Journal of Veterinary Cardiology 2015;17:S268-S281.

31. Gundler S, Tidholm A, Haggstrom J. Prevalence of myocardial hypertrophy in a population of asymptomatic Swedish Maine coon cats. Acta Veterinaria Scandinavica 2008;50.

32. Hanås S, Holst BS, Höglund K, Häggström J, Tidholm A, Ljungvall I. Effect of feline characteristics on plasma N-terminal-prohormone B-type natriuretic peptide concentration and comparison of a point-of-care test and an ELISA test. Journal of Veterinary Internal Medicine 2020;34:1187-1197.

33. Herndon WE, Kittleson MD, Sanderson K et al. Cardiac troponin I in feline hypertrophic cardiomyopathy. J Vet Intern Med 2002;16:558-64.

34. Hertzsch S, Roos A, Wess G. Evaluation of a sensitive cardiac troponin I assay as a screening test for the diagnosis of hypertrophic cardiomyopathy in cats. Journal of Veterinary Internal Medicine 2019;33:1242-1250.

35. Hori Y, Iguchi M, Heishima Y et al. Diagnostic utility of cardiac troponin I in cats with hypertrophic cardiomyopathy. Journal of Veterinary Internal Medicine 2018;32:922-929.

36. Jackson BL, Adin DB, Lehmkuhl LB. Effect of atenolol on heart rate, arrhythmias, blood pressure, and dynamic left ventricular outflow tract obstruction in cats with subclinical hypertrophic cardiomyopathy. Journal of Veterinary Cardiology 2015;17:S296-S305.

37. Kershaw O, Heblinski N, Lotz F, Dirsch O, Gruber AD. Diagnostic value of morphometry in feline hypertrophic cardiomyopathy. J Comp Pathol 2012;147:73-83.

38. Khor KH, Campbell FE, Owen H, Shiels IA, Mills PC. Myocardial collagen deposition and inflammatory cell infiltration in cats with pre-clinical hypertrophic cardiomyopathy. Vet J 2015;203:161-8.

39. Kittleson MD, Meurs KM, Munro MJ et al. Familial hypertrophic cardiomyopathy in maine coon cats: an animal model of human disease. Circulation 1999;99:3172-80.

40. Kitz S, Fonfara S, Hahn S, Hetzel U, Kipar A. Feline Hypertrophic Cardiomyopathy: The Consequence of Cardiomyocyte-Initiated and Macrophage-Driven Remodeling Processes? Vet Pathol 2019;56:565-575.

41. Kochie SL, Schober KE, Rhinehart J et al. Effects of pimobendan on left atrial transport function in cats. J Vet Intern Med 2021;35:10-21.

42. Koffas H, Dukes-McEwan J, Corcoran BM et al. Pulsed tissue Doppler imaging in normal cats and cats with hypertrophic cardiomyopathy. Journal of Veterinary Internal Medicine 2006;20:65-77.

43. Koffas H, Dukes-McEwan J, Corcoran BM et al. Colour M-mode tissue Doppler imaging in healthy cats and cats with hypertrophic cardiomyopathy. Journal of Small Animal Practice 2008;49:330-338.

44. Kostiuk O, Tsvilihovsky M, Enciu V, Melnyk O. Prevalence of hypertrophic cardiomyopathy in a population of cats in Ukraine. Revista Romana De Medicina Veterinara 2020;30:66-70.

45. Langhorn R, Tarnow I, Willesen JL, Kjelgaard-Hansen M, Skovgaard IM, Koch J. Cardiac troponin I and T as prognostic markers in cats with hypertrophic cardiomyopathy. J Vet Intern Med 2014;28:1485-91.

46. Lean FZX, Priestnall SL, Vitores AG, Suarez-Bonnet A, Brookes SM, Nunez A. Elevated angiotensin-converting enzyme 2 (ACE2) expression in cats with hypertrophic cardiomyopathy. Res Vet Sci 2022;152:564-568.

47. Liu SK, Roberts WC, Maron BJ. Comparison of morphologic findings in spontaneously occurring hypertrophic cardiomyopathy in humans, cats and dogs. Am J Cardiol 1993;72:944-51.

48. Liu MM, Eckersall PD, Mrljak V et al. Novel biomarkers in cats with congestive heart failure due to primary cardiomyopathy. Journal of Proteomics 2020;226.

49. MacDonald KA, Kittleson MD, Garcia-Nolen T, Larson RF, Wisner ER. Tissue Doppler imaging and gradient echo cardiac magnetic resonance imaging in normal cats and cats with hypertrophic cardiomyopathy. Journal of Veterinary Internal Medicine 2006;20:627-634.

50. MacDonald KA, Kittleson MD, Kass PH, Meurs KM. Tissue Doppler imaging in Maine Coon cats with a mutation of myosin binding protein C with or without hypertrophy. Journal of Veterinary Internal Medicine 2007;21:232-237.

51. MacDonald KA, Kittleson MD, Kass PH, White SD. Effect of spironolactone on diastolic function and left ventricular mass in Maine Coon cats with familial hypertrophic cardiomyopathy. J Vet Intern Med 2008;22:335-41.

52. MacLean HN, Abbott JA, Ward DL, Huckle WR, Sisson DD, Pyle RL. N-terminal atrial natriuretic peptide immunoreactivity in plasma of cats with hypertrophic cardiomyopathy. J Vet Intern Med 2006;20:284-9.
53. Mary J, Chetboul V, Sampedrano CC et al. Prevalence of the MYBPC3-A31P mutation in a large European feline population and association with hypertrophic cardiomyopathy in the Maine Coon breed. Journal of Veterinary Cardiology 2010;12:155-161.
54. Marz I, Wilkie LJ, Harrington N et al. Familial cardiomyopathy in Norwegian Forest cats. Journal of Feline Medicine and Surgery 2015;17:681-691.
55. McNamara JW, Schuckman M, Becker RC, Sadayappan S. A Novel Homozygous Intronic Variant in TNNT2 Associates With Feline Cardiomyopathy. Front Physiol 2020;11:608473.
56. Messer AE, Chan J, Daley A, Copeland O, Marston SB, Connolly DJ. Investigations into the Sarcomeric Protein and Ca(2+)-Regulation Abnormalities Underlying Hypertrophic Cardiomyopathy in Cats (Felix catus). Front Physiol 2017;8:348.
57. Meurs KM, Sanchez X, David RM et al. A cardiac myosin binding protein C mutation in the Maine Coon cat with familial hypertrophic cardiomyopathy. Hum Mol Genet 2005;14:3587-93.
58. Meurs KM, Norgard MM, Ederer MM, Hendrix KP, Kittleson MD. A substitution mutation in the myosin binding protein C gene in ragdoll hypertrophic cardiomyopathy. Genomics 2007;90:261-4.
59. Meurs KM, Williams BG, DeProspero D et al. A deleterious mutation in the ALMS1 gene in a naturally occurring model of hypertrophic cardiomyopathy in the Sphynx cat. Orphanet J Rare Dis 2021;16:108.

60. Novo Matos J, Garcia-Canadilla P, Simcock IC et al. Micro-computed tomography (micro-CT) for the assessment of myocardial disarray, fibrosis and ventricular mass in a feline model of hypertrophic cardiomyopathy. Sci Rep 2020;10:20169.
61. Ontiveros ES, Ueda Y, Harris SP, Stern JA. Precision medicine validation: identifying the MYBPC3 A31P variant with whole-genome sequencing in two Maine Coon cats with hypertrophic cardiomyopathy. Journal of Feline Medicine and Surgery 2018.
62. Payne JR, Borgeat K, Connolly DJ et al. Prognostic Indicators in Cats with Hypertrophic Cardiomyopathy. Journal of Veterinary Internal Medicine 2013;27:1427-1436.
63. Payne JR, Brodbelt DC, Luis Fuentes V. Cardiomyopathy prevalence in 780 apparently healthy cats in rehoming centres (the CatScan study). J Vet Cardiol 2015;17:S244-57.
64. Payne JR, Borgeat K, Brodbelt DC, Connolly DJ, Luis Fuentes V. Risk factors associated with sudden death vs. congestive heart failure or arterial thromboembolism in cats with hypertrophic cardiomyopathy. J Vet Cardiol 2015;17:S318-28.
65. Pellegrino A, Daniel AGT, Pereira GG, Itikawa PH, Larsson MHMA. Assessment of regional left ventricular systolic function by strain imaging echocardiography in phenotypically normal and abnormal Maine coon cats tested for the A31p mutation in the MYBPC3 gene. Canadian Journal of Veterinary Research 2017;81:137-146.
66. Ramirez-Hernandez C, Barbosa-Quintana A, Ramirez-Romero R. Left Ventricular Apical Aneurysm in a Cat With Primary Cardiomyopathy. Veterinary Pathology 2017;54:254-257.
67. Riesen SC, Kovacevicz A, Lombard CW, Amberger C. Prevalence of heart disease in symptomatic cats: an overview from 1998 to 2005. Schweizer Archiv Fur Tierheilkunde 2007;149:65-71.

68. Rodriguez JMM, Fonfara S, Hetzel U, Kipar A. Feline hypertrophic cardiomyopathy: reduced microvascular density and involvement of CD34+ interstitial cells. Vet Pathol 2022;59:269-283.

69. Sampedrano CC, Chetboul V, Gouni V, Nicolle AP, Pouchelon JL, Tissier R. Systolic and diastolic myocardial dysfunction in cats with hypertrophic cardiomyopathy or systemic hypertension. Journal of Veterinary Internal Medicine 2006;20:1106-1115.

70. Schipper T, Van Poucke M, Sonck L et al. A feline orthologue of the human MYH7 c.5647G > A (p.(Glu1883Lys)) variant causes hypertrophic cardiomyopathy in a Domestic Shorthair cat. European Journal of Human Genetics 2019;27:1724-1730.

71. Schober KE, Zientek J, Li XB, Fuentes VL, Bonagura JD. Effect of treatment with atenolol on 5-year survival in cats with preclinical (asymptomatic) hypertrophic cardiomyopathy. Journal of Veterinary Cardiology 2013;15:93-104.

72. Schober KE, Savino SI, Yildiz V. Right ventricular involvement in feline hypertrophic cardiomyopathy. J Vet Cardiol 2016;18:297-309.

73. Seo J, Payne JR, Novo Matos J, Fong WW, Connolly DJ, Luis Fuentes V. Biomarker changes with systolic anterior motion of the mitral valve in cats with hypertrophic cardiomyopathy. J Vet Intern Med 2020;34:1718-27.

74. Sharpe AN, Oldach MS, Kaplan JL et al. Pharmacokinetics of a single dose of Aficamten (CK-274) on cardiac contractility in a A31P MYBPC3 hypertrophic cardiomyopathy cat model. J Vet Pharmacol Ther 2023;46:52-61.

75. Sharpe AN, Oldach MS, Rivas VN et al. Effects of Aficamten on cardiac contractility in a feline translational model of hypertrophic cardiomyopathy. Sci Rep 2023;13:32.

76. Silverman SJ, Stern JA, Meurs KM. Hypertrophic cardiomyopathy in the Sphynx cat: A retrospective evaluation of clinical presentation and heritable etiology. Journal of Feline Medicine and Surgery 2012;14:246-249.

77. Steele MM, Borgeat K, Payne JR et al. Increased insulin-like growth factor 1 concentrations in a retrospective population of non-diabetic cats diagnosed with hypertrophic cardiomyopathy. J Feline Med Surg 2021;23:952-958.
78. Stern JA, Markova S, Ueda Y et al. A Small Molecule Inhibitor of Sarcomere Contractility Acutely Relieves Left Ventricular Outflow Tract Obstruction in Feline Hypertrophic Cardiomyopathy. PLoS One 2016;11:e0168407.
79. Sugimoto K, Fujii Y, Sunahara H, Aoki T. Assessment of left ventricular longitudinal function in cats with subclinical hypertrophic cardiomyopathy using tissue Doppler imaging and speckle tracking echocardiography. Journal of Veterinary Medical Science 2015;77:1101-1108.
80. Sugimoto K, Aoki T, Fujii Y. Effects of atenolol on left atrial and left ventricular function in healthy cats and in cats with hypertrophic cardiomyopathy. Journal of Veterinary Medical Science 2020;82:546-552.
81. Sukumolanan P, Petchdee S. Prevalence of cardiac myosin-binding protein C3 mutations in Maine Coon cats with hypertrophic cardiomyopathy. Vet World 2022;15:502-508.
82. Suzuki R, Mochizuki Y, Yoshimatsu H, Teshima T, Matsumoto H, Koyama H. Determination of multidirectional myocardial deformations in cats with hypertrophic cardiomyopathy by using two-dimensional speckle-tracking echocardiography. Journal of Feline Medicine and Surgery 2017;19:1283-1289.
83. Tablin F, Schumacher T, Pombo M et al. Platelet Activation in Cats with Hypertrophic Cardiomyopathy. Journal of Veterinary Internal Medicine 2014;28:411-418.
84. Taillefer M, Di Fruscia R. Benazepril and subclinical feline hypertrophic cardiomyopathy: A prospective, blinded, controlled study. Canadian Veterinary Journal-Revue Veterinaire Canadienne 2006;47:437-445.

85. Taugner FM. Stimulation of the renin-angiotensin system in cats with hypertrophic cardiomyopathy. Journal of Comparative Pathology 2001;125:122-129.

86. Tilley LP, Liu SK, Gilbertson SR, Wagner BM, Lord PF. Primary myocardial disease in the cat. A model for human cardiomyopathy. Am J Pathol 1977;86:493-522.

87. Trehiou-Sechi E, Tissier R, Gouni V et al. Comparative Echocardiographic and Clinical Features of Hypertrophic Cardiomyopathy in 5 Breeds of Cats: A Retrospective Analysis of 344 Cases (2001-2011). Journal of Veterinary Internal Medicine 2012;26:532-541.

88. van Dijk SJ, Bezold Kooiker K, Mazzalupo S et al. The A31P missense mutation in cardiac myosin binding protein C alters protein structure but does not cause haploinsufficiency. Arch Biochem Biophys 2016;601:133-40.

89. van Hoek I, Hodgkiss-Geere H, Bode EF et al. Associations among echocardiography, cardiac biomarkers, insulin metabolism, morphology, and inflammation in cats with asymptomatic hypertrophic cardiomyopathy. Journal of Veterinary Internal Medicine 2020;34:591-599.

90. Visser LC, Sloan CQ, Stern JA. Echocardiographic Assessment of Right Ventricular Size and Function in Cats With Hypertrophic Cardiomyopathy. Journal of Veterinary Internal Medicine 2017;31:668-677.

91. Wagner T, Fuentes VL, Payne JR, McDermott N, Brodbelt D. Comparison of auscultatory and echocardiographic findings in healthy adult cats. Journal of Veterinary Cardiology 2010;12:171-182.

92. Weber K, Rostert N, Bauersachs S, Wess G. Serum microRNA profiles in cats with hypertrophic cardiomyopathy. Mol Cell Biochem 2015;402:171-80.

93. Wess G, Daisenberger P, Mahling M, Hirschberger J, Hartmann K. Utility of measuring plasma N-terminal pro-brain natriuretic peptide in detecting hypertrophic

cardiomyopathy and differentiating grades of severity in cats. Veterinary Clinical Pathology 2011;40:237-244.

94. Wilkie LJ, Smith K, Fuentes VL. Cardiac pathology findings in 252 cats presented for necropsy; a comparison of cats with unexpected death versus other deaths. Journal of Veterinary Cardiology 2015;17:S329-S340.

95. Yang VK, Freeman LM, Rush JE. Comparisons of morphometric measurements and serum insulin-like growth factor concentration in healthy cats and cats with hypertrophic cardiomyopathy. American Journal of Veterinary Research 2008;69:1061-1066.

Supplemental References iPSC models

1. Agarwal R, Paulo JA, Toepfer CN et al. Filamin C Cardiomyopathy Variants Cause Protein and Lysosome Accumulation. Circ Res 2021;129:751-766.

2. Ben Jehuda R, Eisen B, Shemer Y et al. CRISPR correction of the PRKAG2 gene mutation in the patient's induced pluripotent stem cell-derived cardiomyocytes eliminates electrophysiological and structural abnormalities. Heart Rhythm 2018;15:267-276.

3. Bhagwan JR, Mosqueira D, Chairez-Cantu K et al. Isogenic models of hypertrophic cardiomyopathy unveil differential phenotypes and mechanism-driven therapeutics. J Mol Cell Cardiol 2020;145:43-53.

4. Birket MJ, Ribeiro MC, Kosmidis G et al. Contractile Defect Caused by Mutation in MYBPC3 Revealed under Conditions Optimized for Human PSC-Cardiomyocyte Function. Cell Rep 2015;13:733-745.

5. Cao X, Jahng JWS, Lee C et al. Generation of three induced pluripotent stem cell lines from hypertrophic cardiomyopathy patients carrying MYH7 mutations. Stem Cell Res 2021;55:102455.
6. Chai AC, Cui M, Chemello F et al. Base editing correction of hypertrophic cardiomyopathy in human cardiomyocytes and humanized mice. Nat Med 2023;29:401-411.
7. Chang ACY, Chang ACH, Kirillova A et al. Telomere shortening is a hallmark of genetic cardiomyopathies. Proc Natl Acad Sci U S A 2018;115:9276-9281.
8. Cheng D, Zhang S, Li X, Wang L, Dong J, Sang H. An integration-free iPSC line ZZUNEUi028-A derived from a patient with hypertrophic cardiomyopathy carrying a heterozygous mutation (c. 1504 C > T) in MYBPC3 gene. Stem Cell Res 2022;63:102848.
9. Clippinger SR, Cloonan PE, Wang W et al. Mechanical dysfunction of the sarcomere induced by a pathogenic mutation in troponin T drives cellular adaptation. J Gen Physiol 2021;153.
10. Cohn R, Thakar K, Lowe A et al. A Contraction Stress Model of Hypertrophic Cardiomyopathy due to Sarcomere Mutations. Stem Cell Reports 2019;12:71-83.
11. Dainis A, Zaleta-Rivera K, Ribeiro A et al. Silencing of MYH7 ameliorates disease phenotypes in human iPSC-cardiomyocytes. Physiol Genomics 2020;52:293-303.
12. Dambrot C, Braam SR, Tertoolen LG, Birket M, Atsma DE, Mummery CL. Serum supplemented culture medium masks hypertrophic phenotypes in human pluripotent stem cell derived cardiomyocytes. J Cell Mol Med 2014;18:1509-18.
13. De Jong HN, Dewey FE, Cordero P et al. Wnt Signaling Interactor WTIP (Wilms Tumor Interacting Protein) Underlies Novel Mechanism for Cardiac Hypertrophy. Circ Genom Precis Med 2022;15:e003563.

14. Dementyeva EV, Medvedev SP, Kovalenko VR et al. Applying Patient-Specific Induced Pluripotent Stem Cells to Create a Model of Hypertrophic Cardiomyopathy. Biochemistry (Mosc) 2019;84:291-298.
15. Dementyeva EV, Kovalenko VR, Zhiven MK et al. Generation of two clonal iPSC lines, ICGi019-A and ICGi019-B, by reprogramming peripheral blood mononuclear cells of a patient suffering from hypertrophic cardiomyopathy and carrying a heterozygous p.M659I mutation in MYH7. Stem Cell Res 2020;46:101840.
16. Dong Y, Li X, Fu W et al. Generation of an iPSC line (ZZUNEUi021-A) from a hypertrophic cardiomyopathy patient with TNNT2 mutation. Stem Cell Res 2022;58:102622.
17. Flenner F, Jungen C, Kupker N et al. Translational investigation of electrophysiology in hypertrophic cardiomyopathy. J Mol Cell Cardiol 2021;157:77-89.
18. Fontaine V, Duboscq-Bidot L, Jouve C et al. Generation of iPSC line from MYH7 R403L mutation carrier with severe hypertrophic cardiomyopathy and isogenic CRISPR/Cas9 corrected control. Stem Cell Res 2021;52:102245.
19. Guo G, Fu W, Li X, Dong J, Zhao X, Zhang Y. Generation of an iPSC line (ZZUNEUi016-A) derived from a hypertrophic cardiomyopathy patient with the heterozygote mutation in MYH7 gene. Stem Cell Res 2021;53:102262.
20. Guo J, Jiang H, Oguntuyo K, Rios B, Boodram Z, Huebsch N. Interplay of Genotype and Substrate Stiffness in Driving the Hypertrophic Cardiomyopathy Phenotype in iPSC-Micro-Heart Muscle Arrays. Cell Mol Bioeng 2021;14:409-425.
21. Guo T, Jiang Y, Song Y et al. Generation of a homozygous MYH7 gene knockout human embryonic stem cell line (WAe009-A-69) using an episomal vector-based CRISPR/Cas9 system. Stem Cell Res 2021;57:102566.

22. Han L, Li Y, Tchao J et al. Study familial hypertrophic cardiomyopathy using patient-specific induced pluripotent stem cells. Cardiovasc Res 2014;104:258-69.
23. Helms AS, Tang VT, O'Leary TS et al. Effects of MYBPC3 loss-of-function mutations preceding hypertrophic cardiomyopathy. JCI Insight 2020;5.
24. Holliday M, Ross SB, Lim S et al. Development of induced pluripotent stem cells from a patient with hypertrophic cardiomyopathy who carries the pathogenic myosin heavy chain 7 mutation p.Arg403Gln. Stem Cell Res 2018;33:269-273.
25. Holliday M, Ross SB, Lim S, Semsarian C. Generation of an induced pluripotent stem cell line from a hypertrophic cardiomyopathy patient with a pathogenic myosin binding protein C (MYBPC3) p.Arg502Trp mutation. Stem Cell Res 2018;33:56-59.
26. Hsieh J, Becklin KL, Givens S et al. Myosin Heavy Chain Converter Domain Mutations Drive Early-Stage Changes in Extracellular Matrix Dynamics in Hypertrophic Cardiomyopathy. Front Cell Dev Biol 2022;10:894635.
27. James V, Nizamudeen ZA, Lea D et al. Transcriptomic Analysis of Cardiomyocyte Extracellular Vesicles in Hypertrophic Cardiomyopathy Reveals Differential snoRNA Cargo. Stem Cells Dev 2021;30:1215-1227.
28. Javor J, Ewoldt JK, Cloonan PE et al. Probing the subcellular nanostructure of engineered human cardiomyocytes in 3D tissue. Microsyst Nanoeng 2021;7:10.
29. Jia WW, Lu JZ, Zhang L et al. An induced pluripotent stem cell line (EHTJUi003-A) generated from a neonate with c.1377delC mutation in the gene MYBPC3 causing hypertrophic cardiomyopathy. Stem Cell Res 2021;53:102328.
30. Jin J, Lu L, Chen J et al. Generation of an induced pluripotential stem cell (iPSC) line from a patient with hypertrophic cardiomyopathy carrying myosin binding protein C (MYBPC3) c.3369-3370 insC mutation. Stem Cell Res 2020;50:102144.

31. Juhola M, Joutsijoki H, Penttinen K, Aalto-Setälä K. Detection of genetic cardiac diseases by Ca(2+) transient profiles using machine learning methods. Sci Rep 2018;8:9355.

32. Kang JY, Mun D, Chun Y et al. Generation of a heterozygous TPM1-E192K knock-in human induced pluripotent stem cell line using CRISPR/Cas9 system. Stem Cell Res 2022;63:102878.

33. Kang JY, Mun D, Chun Y et al. Generation of three TTN knock-out human induced pluripotent stem cell lines using CRISPR/Cas9 system. Stem Cell Res 2022;64:102901.

34. Kargaran PK, Evans JM, Bodbin SE et al. Mitochondrial DNA: Hotspot for Potential Gene Modifiers Regulating Hypertrophic Cardiomyopathy. J Clin Med 2020;9.

35. Khalilimeybodi A, Riaz M, Campbell SG et al. Signaling network model of cardiomyocyte morphological changes in familial cardiomyopathy. J Mol Cell Cardiol 2023;174:1-14.

36. Kim H, Kim HJ, Oh J et al. An induced pluripotent stem cell line (YCMi006-A) generated from a patient with hypertrophic cardiomyopathy who carries the ACTA1 mutation p.Ile343Met. Stem Cell Res 2022;63:102874.

37. Kondo T, Higo S, Shiba M et al. Human-Induced Pluripotent Stem Cell-Derived Cardiomyocyte Model for TNNT2 Delta160E-Induced Cardiomyopathy. Circ Genom Precis Med 2022;15:e003522.

38. Lan F, Lee AS, Liang P et al. Abnormal calcium handling properties underlie familial hypertrophic cardiomyopathy pathology in patient-specific induced pluripotent stem cells. Cell Stem Cell 2013;12:101-13.

39. Li S, Pan H, Tan C et al. Mitochondrial Dysfunctions Contribute to Hypertrophic Cardiomyopathy in Patient iPSC-Derived Cardiomyocytes with MT-RNR2 Mutation. Stem Cell Reports 2018;10:808-821.

40. Li X, Liu Y, Liu F et al. Generation of a hiPSC line ZZUNEUi007-A from a patient with hypertrophic cardiomyopathy caused by mutation in MYH7. Stem Cell Res 2020;43:101699.

41. Li X, Fu W, Guo G et al. A heterozygous MYH7 (c. 2156G > A) mutant human induced pluripotent stem cell line (ZZUNEUi020-A) generated from a patient with hypertrophic cardiomyopathy. Stem Cell Res 2021;51:102158.

42. Liang P, Lan F, Lee AS et al. Drug screening using a library of human induced pluripotent stem cell-derived cardiomyocytes reveals disease-specific patterns of cardiotoxicity. Circulation 2013;127:1677-91.

43. Lindholm ME, Jimenez-Morales D, Zhu H et al. Mono- and Biallelic Protein-Truncating Variants in Alpha-Actinin 2 Cause Cardiomyopathy Through Distinct Mechanisms. Circ Genom Precis Med 2021;14:e003419.

44. Liu L, Shenoy SP, Jahng JWS et al. Generation of two heterozygous MYBPC3 mutation-carrying human iPSC lines, SCVIi001-A and SCVIi002-A, for modeling hypertrophic cardiomyopathy. Stem Cell Res 2021;53:102279.

45. Loiben AM, Chien WM, Friedman CE et al. Cardiomyocyte Apoptosis Is Associated with Contractile Dysfunction in Stem Cell Model of MYH7 E848G Hypertrophic Cardiomyopathy. Int J Mol Sci 2023;24.

46. Ma N, Zhang JZ, Itzhaki I et al. Determining the Pathogenicity of a Genomic Variant of Uncertain Significance Using CRISPR/Cas9 and Human-Induced Pluripotent Stem Cells. Circulation 2018;138:2666-2681.

47. Ma Z, Huebsch N, Koo S et al. Contractile deficits in engineered cardiac microtissues as a result of MYBPC3 deficiency and mechanical overload. Nature Biomedical Engineering 2018;2:955-967.

48. Manhas A, Jahng JWS, Vera CD, Shenoy SP, Knowles JW, Wu JC. Generation of two iPSC lines from hypertrophic cardiomyopathy patients carrying MYBPC3 and PRKAG2 variants. Stem Cell Res 2022;61:102774.

49. Margara F, Psaras Y, Wang ZJ et al. Mechanism based therapies enable personalised treatment of hypertrophic cardiomyopathy. Sci Rep 2022;12:22501.

50. Merkert S, Wunderlich S, Beier J et al. Generation of two iPSC clones (MHHi021-A and MHHi021-B) from a patient with hypertrophic cardiomyopathy with p.Arg723Gly mutation in the MYH7 gene. Stem Cell Res 2021;52:102208.

51. Mosqueira D, Mannhardt I, Bhagwan JR et al. CRISPR/Cas9 editing in human pluripotent stem cell-cardiomyocytes highlights arrhythmias, hypocontractility, and energy depletion as potential therapeutic targets for hypertrophic cardiomyopathy. Eur Heart J 2018;39:3879-3892.

52. Ojala M, Prajapati C, Pölönen RP et al. Mutation-Specific Phenotypes in hiPSC-Derived Cardiomyocytes Carrying Either Myosin-Binding Protein C Or α-Tropomyosin Mutation for Hypertrophic Cardiomyopathy. Stem Cells Int 2016;2016:1684792.

53. Okamoto R, Goto I, Nishimura Y et al. Gap junction protein beta 4 plays an important role in cardiac function in humans, rodents, and zebrafish. PLoS One 2020;15:e0240129.

54. Pettinato AM, Ladha FA, Mellert DJ et al. Development of a Cardiac Sarcomere Functional Genomics Platform to Enable Scalable Interrogation of Human TNNT2 Variants. Circulation 2020.

55. Phelan DG, Anderson DJ, Howden SE et al. ALPK3-deficient cardiomyocytes generated from patient-derived induced pluripotent stem cells and mutant human embryonic stem cells display abnormal calcium handling and establish that ALPK3

deficiency underlies familial cardiomyopathy(aEuro). European Heart Journal 2016;37:2586-2590.

56. Pioner JM, Racca AW, Klaiman JM et al. Isolation and Mechanical Measurements of Myofibrils from Human Induced Pluripotent Stem Cell-Derived Cardiomyocytes. Stem Cell Reports 2016;6:885-896.

57. Pioner JM, Vitale G, Steczina S et al. Slower Calcium Handling Balances Faster Cross-Bridge Cycling in Human MYBPC3 HCM. Circ Res 2023;132:628-644.

58. Prajapati C, Ojala M, Aalto-Setälä K. Divergent effects of adrenaline in human induced pluripotent stem cell-derived cardiomyocytes obtained from hypertrophic cardiomyopathy. Dis Model Mech 2018;11.

59. Prondzynski M, Krämer E, Laufer SD et al. Evaluation of MYBPC3 trans-Splicing and Gene Replacement as Therapeutic Options in Human iPSC-Derived Cardiomyocytes. Mol Ther Nucleic Acids 2017;7:475-486.

60. Prondzynski M, Lemoine MD, Zech AT et al. Disease modeling of a mutation in α-actinin 2 guides clinical therapy in hypertrophic cardiomyopathy. EMBO Mol Med 2019;11:e11115.

61. Pua CJ, Tham N, Chin CWL et al. Genetic Studies of Hypertrophic Cardiomyopathy in Singaporeans Identify Variants in TNNI3 and TNNT2 That Are Common in Chinese Patients. Circ Genom Precis Med 2020;13:424-434.

62. Qiu H, Sun Y, Pan Z et al. Inhibition of HSC70 alleviates hypertrophic cardiomyopathy pathology in human induced pluripotent stem cell-derived cardiomyocytes with a MYBPC3 mutation. Clin Transl Med 2021;11:e647.

63. Ramachandra CJA, Kp MMJ, Chua J et al. Inhibiting cardiac myeloperoxidase alleviates the relaxation defect in hypertrophic cardiomyocytes. Cardiovasc Res 2022;118:517-530.

64. Riaz M, Park J, Sewanan LR et al. Muscle LIM Protein Force-Sensing Mediates Sarcomeric Biomechanical Signaling in Human Familial Hypertrophic Cardiomyopathy. Circulation 2022;145:1238-1253.
65. van der Roest AS, Liu C, Morck MM et al. Hypertrophic cardiomyopathy beta-cardiac myosin mutation (P710R) leads to hypercontractility by disrupting super relaxed state. Proc Natl Acad Sci U S A 2021;118.
66. Ross SB, Fraser ST, Nowak N, Semsarian C. Generation of induced pluripotent stem cells (iPSCs) from a hypertrophic cardiomyopathy patient with the pathogenic variant p.Val698Ala in beta-myosin heavy chain (MYH7) gene. Stem Cell Res 2017;20:88-90.
67. Sakai T, Naito AT, Kuramoto Y et al. Phenotypic Screening Using Patient-Derived Induced Pluripotent Stem Cells Identified Pyr3 as a Candidate Compound for the Treatment of Infantile Hypertrophic Cardiomyopathy. Int Heart J 2018;59:1096-1105.
68. Seeger T, Shrestha R, Lam CK et al. A Premature Termination Codon Mutation in MYBPC3 Causes Hypertrophic Cardiomyopathy via Chronic Activation of Nonsense-Mediated Decay. Circulation 2019;139:799-811.
69. Shafaattalab S, Li AY, Gunawan MG et al. Mechanisms of Arrhythmogenicity of Hypertrophic Cardiomyopathy-Associated Troponin T (TNNT2) Variant I79N. Front Cell Dev Biol 2021;9:787581.
70. Smith JGW, Owen T, Bhagwan JR et al. Isogenic Pairs of hiPSC-CMs with Hypertrophic Cardiomyopathy/LVNC-Associated ACTC1 E99K Mutation Unveil Differential Functional Deficits. Stem Cell Reports 2018;11:1226-1243.
71. Strimaityte D, Tu C, Yanez A et al. Contractility and Calcium Transient Maturation in the Human iPSC-Derived Cardiac Microfibers. ACS Appl Mater Interfaces 2022;14:35376-35388.

72. Sun Y, Zhou J, Wang H et al. Establishment of an induced pluripotent stem cell line (ZJULLi004-A) from a hypertrophic cardiomyopathy patient carrying MYBPC3/c.3764C>A mutation. Stem Cell Res 2022;64:102898.

73. Tanaka A, Yuasa S, Mearini G et al. Endothelin-1 induces myofibrillar disarray and contractile vector variability in hypertrophic cardiomyopathy-induced pluripotent stem cell-derived cardiomyocytes. J Am Heart Assoc 2014;3:e001263.

74. Tian X, Fu W, Guo G et al. Generation of a human iPSC line ZZUNEUi015-A from a patient with hypertrophic cardiomyopathy caused by mutation in ALPK3. Stem Cell Res 2021;52:102247.

75. Toepfer CN, Garfinkel AC, Venturini G et al. Myosin Sequestration Regulates Sarcomere Function, Cardiomyocyte Energetics, and Metabolism, Informing the Pathogenesis of Hypertrophic Cardiomyopathy. Circulation 2020;141:828-842.

76. van Helden RWJ, Birket MJ, Freund C et al. Generation of three human induced pluripotent stem cell lines, LUMCi024-A, LUMCi025-A, and LUMCi026-A, from two patients with combined oxidative phosphorylation deficiency 8 and a related control. Stem Cell Res 2021;53:102374.

77. Wang L, Li X, Fu W et al. Generation of an IPSC line from a patient with hypertrophic cardiomyopathy carrying a mutation in MYH6 gene. Stem Cell Res 2020;50:102138.

78. Wang Y, Xiong W, Zhao S, Li B, Chang ACY. Generation of two induced pluripotent stem cell lines, SHIPMi001-A from a patient with hypertrophic cardiomyopathy caused by MYBPC3 gene mutation and SHIPMi002-A from a healthy male individual. Stem Cell Res 2021;57:102594.

79. Warnecke N, Ulmer BM, Laufer SD et al. Generation of bi-allelic MYBPC3 truncating mutant and isogenic control from an iPSC line of a patient with hypertrophic cardiomyopathy. Stem Cell Res 2021;55:102489.

80. Wu H, Yang H, Rhee JW et al. Modelling diastolic dysfunction in induced pluripotent stem cell-derived cardiomyocytes from hypertrophic cardiomyopathy patients. Eur Heart J 2019;40:3685-3695.
81. Yang KC, Breitbart A, De Lange WJ et al. Novel Adult-Onset Systolic Cardiomyopathy Due to MYH7 E848G Mutation in Patient-Derived Induced Pluripotent Stem Cells. JACC Basic Transl Sci 2018;3:728-740.
82. Zech ATL, Prondzynski M, Singh SR et al. ACTN2 Mutant Causes Proteopathy in Human iPSC-Derived Cardiomyocytes. Cells 2022;11.
83. Zhang C, Shi J, Zhang Z et al. Generation of an induced pluripotent stem cell line (SYSUi005-A) from a patient with hypertrophic cardiomyopathy. Stem Cell Res 2022;58:102626.
84. Zhao SR, Shen M, Lee C et al. Generation of three induced pluripotent stem cell lines from hypertrophic cardiomyopathy patients carrying TNNI3 mutations. Stem Cell Res 2021;57:102597.
85. Zhao X, Cao J, Li X et al. A heterozygous MYBPC3 (c. 772+1G > A) mutant human induced pluripotent stem cell line (ZZUNEUi025-A) generated from a male patient with hypertrophic cardiomyopathy. Stem Cell Res 2022;60:102722.
86. Zhou W, Bos JM, Ye D et al. Induced Pluripotent Stem Cell-Derived Cardiomyocytes from a Patient with MYL2-R58Q-Mediated Apical Hypertrophic Cardiomyopathy Show Hypertrophy, Myofibrillar Disarray, and Calcium Perturbations. J Cardiovasc Transl Res 2019;12:394-403.
87. Zhou J, Sun Y, Wang H et al. Generation of an induced pluripotent stem cell line (ZJULLi003-A) from a hypertrophic cardiomyopathy patient carrying MYH7/c.4384G > A mutation. Stem Cell Res 2022;64:102883.
